# A unique peptide deformylase platform to rationally design and challenge novel active compounds

**DOI:** 10.1038/srep35429

**Published:** 2016-10-20

**Authors:** Sonia Fieulaine, Rodolphe Alves de Sousa, Laure Maigre, Karim Hamiche, Mickael Alimi, Jean-Michel Bolla, Abbass Taleb, Alexis Denis, Jean-Marie Pagès, Isabelle Artaud, Thierry Meinnel, Carmela Giglione

**Affiliations:** 1 Institute for Integrative Biology of the Cell (I2BC), CEA, CNRS, Univ. Paris-Sud, Université Paris-Saclay, 91198 Gif-sur-Yvette cedex, France; 2 UMR8601, LCBPT, CNRS Université Paris Descartes, PRES Paris Cité, 45 rue des Sts Pères 75270 Paris Cedex 06, France; 3 UMR_MD1, Aix-Marseille Univ, IRBA, Facultés de Médecine et de Pharmacie, 27 Bd Jean Moulin, 13385 Marseille Cedex 05, France; 4 GlaxoSmithKline, ZA Courtaboeuf, 25 Avenue du Québec, 91140 Villebon-sur-Yvette, France

## Abstract

Peptide deformylase (PDF) is considered an excellent target to develop antibiotics. We have performed an extensive characterization of a new PDF from the pathogen *Streptococcus agalactiae*, showing properties similar to other known PDFs. *S. agalactiae* PDF could be used as PDF prototype as it allowed to get complete sets of 3-dimensional, biophysical and kinetic data with virtually any inhibitor compound. Structure-activity relationship analysis with this single reference system allowed us to reveal distinct binding modes for different PDF inhibitors and the key role of a hydrogen bond in potentiating the interaction between ligand and target. We propose this protein as an irreplaceable tool, allowing easy and relevant fine comparisons between series, to design, challenge and validate novel series of inhibitors. As proof-of-concept, we report here the design and synthesis of effective specific bacterial PDF inhibitors of an oxadiazole series with potent antimicrobial activity against a multidrug resistant clinical isolate.

There is consensus that we urgently need new antibiotics and there are at least two major explanations for this^1^,^2^,^3^,^4^. First, the emerging bacterial multidrug resistances to existing antibiotic drugs and second the inefficiency of the available therapeutic arsenal to kill multiresistant pathogenic strains causing hospital-acquired infectious diseases such as those induced by infections with *Staphylococcus aureus*^5^. Despite extensive efforts to identify novel lead compounds from molecular targets, only few compounds, including peptide deformylase inhibitors (PDFIs) have shown real potential^6^,^7^.

PDFs are ubiquitous essential metalloenzymes which co-translationally remove the formyl group carried by the initiator methionine in bacteria, mitochondria and chloroplast^8^,^9^,^10^. Since they are essential for cell growth, believed to be exclusive of bacteria and not yet the target of any drug in-use, PDFs were initially thought to be very promising for the development of new antibacterial drugs^11^,^12^,^13^,^14^. Identification and characterization of functional PDF homologs in eukaryotes^15^,^16^,^17^,^18^, especially a mitochondrial isoform in human^19^,^20^,^21^,^22^,^23^,^24^, have challenged the use of this enzyme as a relevant antibiotic target. However, enzymatic and structural studies have evidenced several significant differences between prokaryotic and organellar PDF ligand binding sites^16^,^19^,^25^,^26^,^27^, validating this enzyme as a very interesting and relevant therapeutic target. Specifically, the narrower S1’ binding pocket of mitochondrial PDFs compared to bacterial PDFs was exploited to design several compounds highly effective against bacterial PDFs and with no inhibition effect against human PDF^28^,^29^,^30^.

All PDFs are very efficiently inhibited by the natural compound actinonin (Fig. 1a), the first potent PDF inhibitor characterized, showing a dissociation constant in the low nanomolar range^31^. Actinonin cannot be used in therapeutics for several reasons: (i) it targets also several metalloenzymes in addition to PDFs^32^,^33^, (ii) it induces apoptosis and other cytotoxic effects^21^,^24^,^28^,^34^,^35^, (iii) it is rapidly exported by bacterial efflux pumps and therefore exhibits moderate *in vivo* activity^36^,^37^,^38^ and (iv) it induces resistance with a relatively high frequency through various mechanisms most often leading to a by-pass of the formylation pathway^39^,^40^,^41^,^42^,^43^. Nevertheless, actinonin remains the reference compound for the rational design of new anti-PDF compounds because of its high potency attained through a two-step, time-dependent inhibition mechanism called slow tight-binding. In this context, actinonin (Inhibitor, I) initially and rapidly binds the enzyme (E) with a rather low inhibitory constant (*K*_I_). The formed encounter complex (EI) thus evolves towards a second more tightly held complex (EI*), characterized by a high final affinity *K*_I_* (*K*_I_* ≪ *K*_I_) and a very slow off-rate *k*_6_^44^. It has been shown that the tightening of the complex is due to an induced-fit process, in which binding of actinonin drives the reshaping of the ligand binding site strengthening as a result the enzyme-inhibitor interactions network^45^. Slow tight-binding inhibitors are very appealing in therapeutics since they closely mimic the substrate recognition process and lead to very long-lasting inhibitory effects^46^.

A number of new PDFIs have been proven to display specificity towards bacterial PDFs, and to efficiently inhibit the enzymes *in vitro* with IC_50_ or *K*_I_ values in the low nanomolar range^47^,^48^,^49^. Interestingly, most of the potent anti-PDF molecules exhibit slow tight-binding behavior^28^,^44^,^50^,^51^. In addition many PDF inhibitors induce moderate cytotoxic effects^48^ and display antimicrobial activity, particularly when used together with membrane permeabilizers^37^,^38^. Moreover, synergistic effects have been observed when two drugs were applied at the same time^38^,^52^.

After almost 20 years of intensive searching of potent effective PDF inhibitors (PDFIs) only three compounds have entered clinical trials. Unlike compounds BB-83698 and LBM-415 which were discontinued after Phase I, compound GSK1322322 entered Phase II as it is believed to have a suitable activity spectrum for therapeutic use against respiratory tract, skin and soft tissue infections^49^. One reason to explain the failure in designing a higher number of effective PDF inhibitors, which could have reached a successful clinical trial, is the lack of a suitable platform to compare and better design these new PDFIs. Indeed, the critical conception of new efficient drugs relies on the needs to fully elucidate the way they act on their targets and to correlate rationally their structure to activity. Unfortunately, the available data on the identified PDFIs are scattered, preventing the proper improvement of existing molecules.

Here, we present and characterize a PDF enzyme issued from the bacterium *Streptococcus agalactiae (Sa*PDF) which behaves as an excellent structural platform to describe PDF-inhibitor interactions. We have used *Sa*PDF to fully characterize and compare several series of new non-peptidic and pseudo-peptidic PDF inhibitors^28^,^29^, including the resolution of the crystal structure of different complexes between *Sa*PDF and most of these inhibitors. The synergic application of a variety of complementary biochemical approaches along with the X-ray crystallography data analysis allowed to: (i) measure dissociation and inhibition constants for each PDFI (ii) describe their binding site at atomic resolution and (iii) visualize a conformational change induced by one ligand. Together, these results revealed distinct binding modes for the different PDFIs and provide additional criteria defining the basic chemical structure that any PDF inhibitor should display in order to reach high efficiency. Among the analyzed compounds, we identified promising inhibitors derived from an oxadiazole core displaying higher activity against clinical resistant strains compared to the antibiotics currently in use.

## Results and Discussion

### Characterization of *Streptococcus agalactiae* PDF (*Sa*PDF)

In order to evaluate the inhibitory properties of new putative anti-PDF molecules, we characterized a new type 2 PDF (PDF types are described in ref. 53). To get a relevant PDF originating from pathogenic bacteria, we chose the *defB*-encoded PDF2 from the Gram positive bacterium *Streptococcus agalactiae (Sa*PDF), whose infections cause neonatal sepsis and meningitis in newborn infants^54^. In addition, unlike this isoform, the other PDF homolog of *S. agalactiae (defA*-encoded) proved not to be functional in a PDF complementation assay (see ref. 55). This situation is similar of that found in *Streptococcus pneumonia* and *Staphylococcus aureus* where only *defB* is functional^40^. In other words, PDF2s appear the only active PDFs in *Streptoccus* and *Staphyloccus* spp. Recombinant *Sa*PDF was cloned, expressed and purified using classic protocols taking advantage of adding nickel in lysis and purification buffers to avoid oxidation of the essential metal-bound Cys of motif II^56^. The recombinant protein displayed high deformylase activity (*k*_cat_/*K*_m_ = 41,667 M^−1^s^−1^), similar to that of all other bacterial PDFs (Supplementary Table 1).

**Figure 1:**
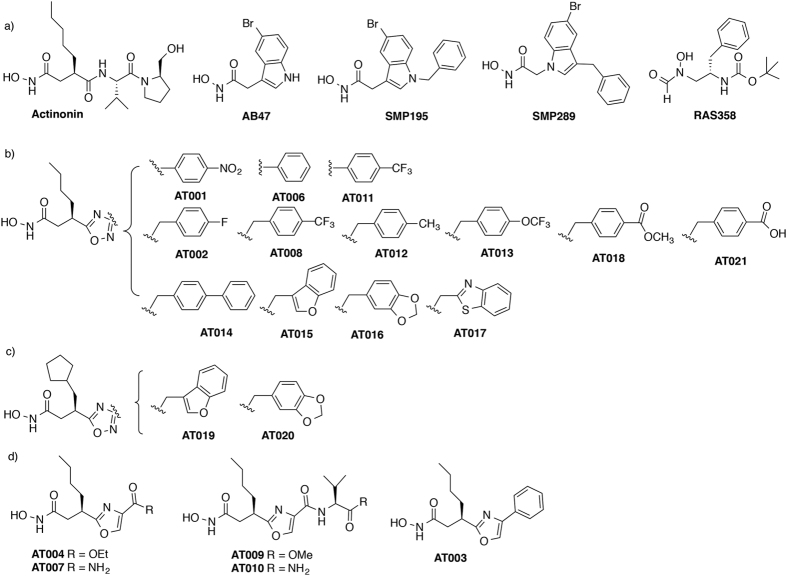
Chemical structures of PDF inhibitors. (**a**) compounds synthesized in previous studies. (**b,c**) new compounds of the oxadiazole series with either an n-butyl (**b)** or a cyclopentyl (**c**) at P1’. (**d**) new compounds of the oxazole series.

As expected, actinonin proved to be a potent inhibitor of *Sa*PDF. In addition, actinonin inhibition of *Sa*PDF involved slow-tight binding (*K*_I*app_ = 1.0 ± 0.1 nM, *K*_I_ = 64 ± 5 nM) (Table 1). The enhancement potency (*K*_I_/*K*_I*app_ = 64) was of the same order of magnitude as those observed with *Ec*PDF and *At*PDF1B (Table 1). Compound AB47 (Fig. 1a), a potent inhibitor specific of bacterial PDFs with IC_50_ values in the low nanomolar range, was previously shown to (i) inhibit *Bacillus stearothermophilus* PDF2 (*Bs*PDF) through slow tight-binding mechanism, and (ii) display antibacterial activity againts *B. subtilis*^28^. Variants of these compounds have been synthesized and two of them have proved very efficient: SMP195 (Fig. 1a) is very potent against purified *Ec*PDF and displays high antibacterial activity towards *B. subtilis*^29^ whereas SMP289 (Fig. 1a), a morphomimetic of SMP195 in which substituents at positions 1 and 3 of the indole are reversed, is specific of *Bs*PDF with however weak potency against *Ec*PDF^29^. In this study, AB47 and SMP195 were found poorly effective against *Sa*PDF, although it is of the same type as *Bs*PDF (Table 2). This indicated that this series had an inhibition potency much depending on the PDF active site content. How the series bound to a PDF was of strong interest in this respect.

### Structural investigation of *Sa*PDF without and with PDFI

**Figure 2:**
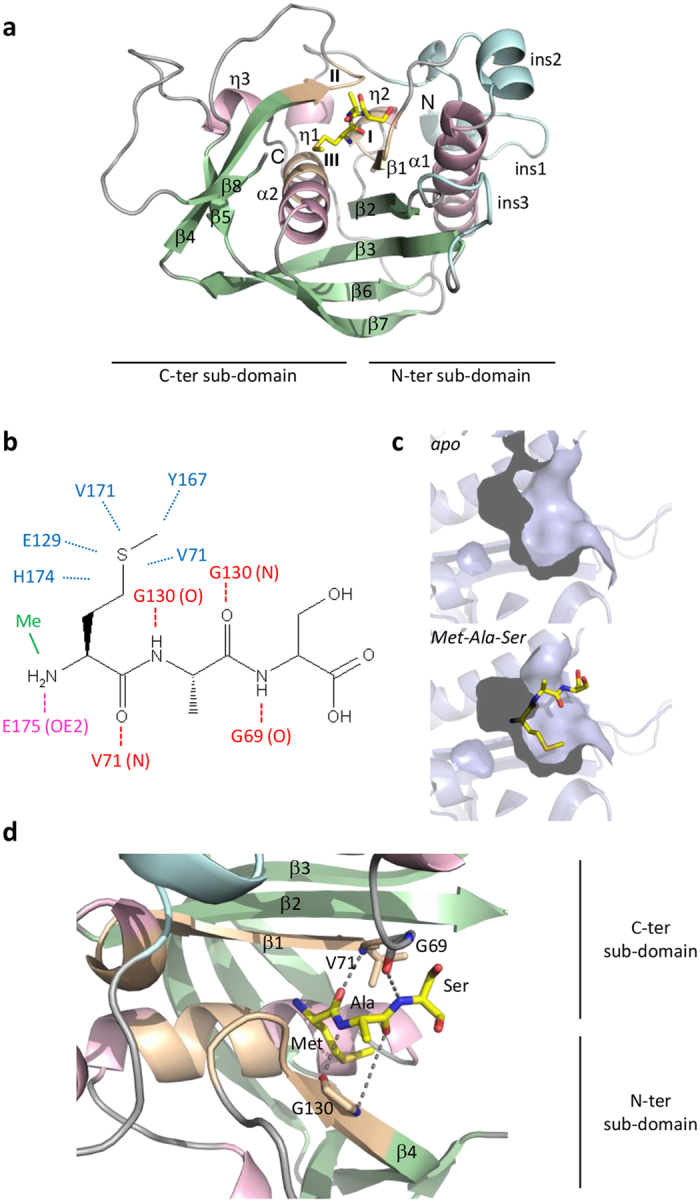
*Sa*PDF substrate binding site. (**a**) Tripeptide Met-Ala-Ser bound to *Sa*PDF between N- and C-terminal sub-domains is shown in sticks format and colored in yellow, with O in red, N in blue, and S in yellow. α and 3_10_ helices of protein are in pink, β strands in green and insertions in light blue. The three consensus motifs I, II and III are colored in light orange. (**b**) The network of interactions of the ligand binding site of Met-Ala-Ser in *Sa*PDF is represented: the free amine group coordinates the catalytic metal (Me) and is hydrogen bonded to Glu175; the side chain of Met fits into a hydrophobic pocket called S1’, made of residues Val71, Leu125, Glu129, Tyr167, Val171, His174; the backbone of peptide is hydrogen bonded to Val71, Gly69 and Gly130 of *Sa*PDF. Dotted lines represent the hydrogen bonds. (**c**) The solvent-accessible surface of S1’ pocket is represented, in apo *Sa*PDF (top) and in the complex with Met-Ala-Ser (bottom). (**d**) Close-up view of hydrogen bonds that link the backbone of Met-Ala-Ser with residues Val71, Gly69 and Gly130 of *Sa*PDF, linking strands β1 and β4. The color code of panel a is used.

Like most other PDFs, *Sa*PDF behaves as a monomer (Supplementary Fig. 1a) and its crystal structure reveals a classical PDF fold (Supplementary Fig. 1b). Like other PDFs^10^, the core of *Sa*PDF is composed of two α-helices, three 3_10_-helices and eight β-strands organized in two β-sheets (Supplementary Fig. 1b,c). In addition, *Sa*PDF contains the typical insertions of type 2 PDFs^12^, including an eight-amino acids loop folded as an α-helix (insertion 2) and a long loop connecting strands β2 and β3 usually referred as the CD-loop (insertion 3). It also displays the classical C-terminal extended fold of type 2 PDFs. The structure is organized into two sub-domains around the active site, called N- and C-terminal sub-domains (Supplementary Fig. 1b,c). The apo-protein crystallized in two different crystallization conditions, which gave highly similar structures (Supplementary Fig. 1d; root mean square deviation between all Cα is 0.307 Å). In a previous work, we have evidenced the flexibility of PDF enzymes around the ligand binding site and defined the aperture angle (δ_ap_) which allows quantifying the extent of protein aperture/closure (see definition of δap and Fig. 2 in ref. 45). According to this measure, both free *Sa*PDF crystallized in a slightly open conformation (δ_ap_ measured between C131, H178 and V71 is respectively 48.0° and 47.5°). The ligand binding site is localized at the interface between the N and the C sub-domains, as evidenced by the structures of the complexes between *Sa*PDF and synthetic tripeptides (Fig. 2a).

Crystal-soaking experiments were conducted with formylated peptides (Fo-Met-Ala-Ser and Fo-Met-Ala-Arg) but led to deformylated molecules in the crystal structures as previously reported for other PDFs^26^,^27^. This is due to the ability of the enzyme to deformylate formylated substrates in the crystal packing. Peptides, which therefore represent deformylation reaction products, were found bonded to *Sa*PDF through a high number of non-covalent interactions, which are similar to those found previously with *Escherichia coli* PDF (*Ec*PDF)^57^. Indeed, the free N-terminal amine group coordinated catalytic ion and was hydrogen bonded to the catalytic residue Glu175 (Fig. 2b). The hydrophobic methionine side chain fitted into a deep hydrophobic S1’ pocket (Fig. 2c,d) composed of several conserved residues (Val71, Leu125, Glu129, Tyr167, Val171, His174). Finally, peptides were in a conformation mimicking a β-strand which was hydrogen-bonded with the main backbone peptide bond of Gly130 from conserved motif II (E_129_GCLS_133_), Val71 from conserved motif I (G_70_VGLAAPQ_77_), and Gly69 which preceds motif I (Fig. 2b), linking the two β-sheets from N- and C-terminal sub-domains (Fig. 2d).

**Figure 3:**
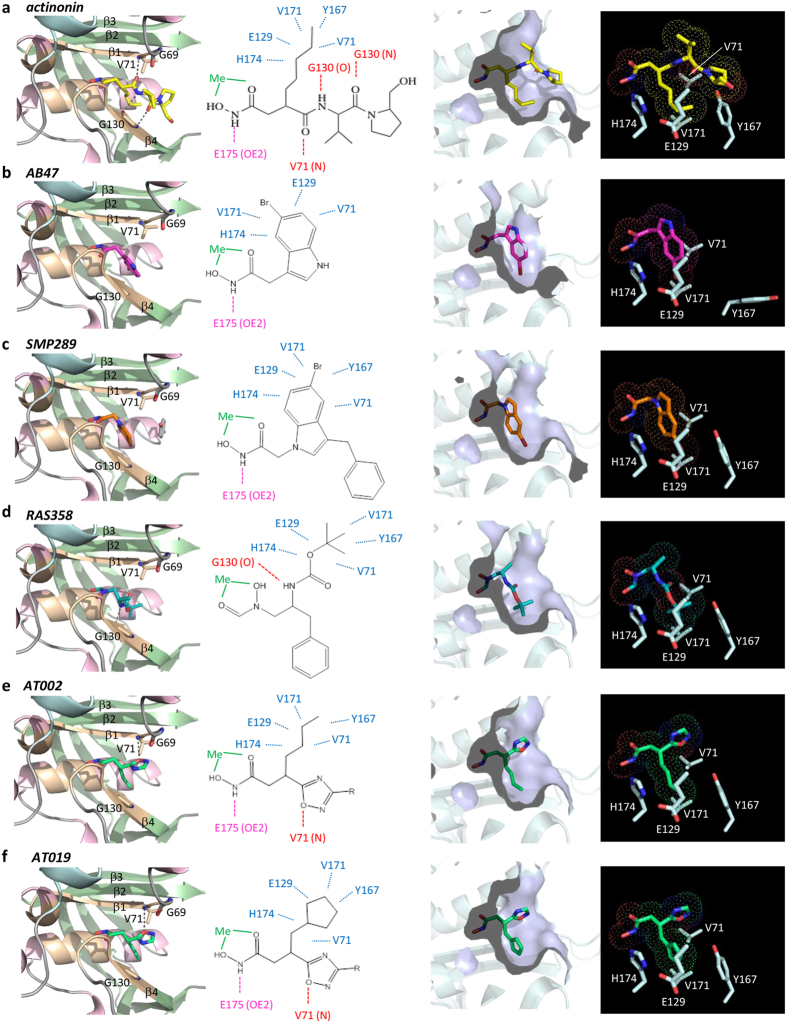
Binding mode of various PDFIs, with various scaffolds. Binding mode of PDFIs in *Sa*PDF is represented. From the left to the right: close-up view of each PDFI within the ligand binding site, with the color code used in Fig.1a for protein; network of interactions of each PDFI; solvent-accessible surface of S1’ pocket in presence of each PDFI; volume occupied by each PDFI in the vicinity of some key residues coming from S1’ pocket. (**a**) actinonin. (**b**) AB47. **(c)** SMP289. (**d**) RAS358. (**e,f**) AT002 and AT019; P3’ group of oxadiazoles being flexible, only P1’ and P2’ are visible (see Supplementary Fig. 2c). Consequently, modeled part of AT002 and AT018 on the one hand and AT019 and AT020 on the other hand are identical (see Fig. 2e,f) and were found fully superimposable in both structures (see Supplementary Fig. 2c). Only AT002 and AT019 are represented here.

We could solve high resolution structures (1.6 to 2.4 Å, see Supplementary Table 2) of *Sa*PDF bound to all of our selection of inhibitors which included actinonin, AB47, SMP289 and RAS358 (Fig. 3a–d). Compared to other bacterial and eukaryotic PDF systems, previously assessed and crystallized^26^,^27^,^45^,^58^, *Sa*PDF proved to be an exceptional structural platform for the comparison of interactions and mode of action of different molecules at atomic level. All compounds could be observed into the electron density maps (Supplementary Fig. 2), bound in the substrate binding site of the protein at the peptides binding site observed in crystal structures described in the previous paragraph (Fig. 3 left panels).

**Figure 4:**
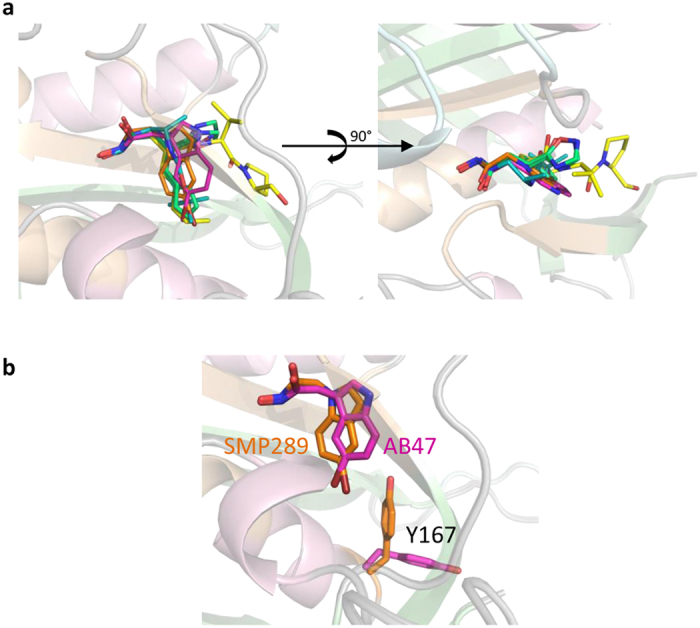
3D comparison of the binding between various PDFIs and actinonin. (**a**) Overall superimposition of actinonin, AT002, AT019, AT020, AB47, SMP289 and RAS358. The color code used is that of Fig. 1a for *Sa*PDF and Fig. 3 for PDFIs. Two distinct orientations are displayed. (**b**) Comparison of the Tyr167 side chain position of *Sa*PDF in complex with AB47 (pink) or SMP289 (orange).

We have previously shown that AB47 binds to the conventional ligand binding site of *A. thaliana* PDF1B (*At*PDF1B)^45^. When bound to *Sa*PDF, AB47 reproduced the interactions observed with *At*PDF1B through only its hydroxamate and bromoindole P1’ group (Fig. 3b). No hydrogen bond mimicking additional β-strand for protein β-sheets was observed due to the absence of a carbonyl group. An interesting feature arises from the binding of AB47 and SMP289. Both molecules are morphomimetic with the hydroxamate group branched on an opposite direction relative to the bromine on the indole group (Fig. 1a). As a result, the bromine atoms are not superimposable if one considers the hydroxamate as the reference of the orientation either in the plan or in the binding pocket (Fig. 1a). Due to the bulkiness and hydrophobic character of the bromine, our data showed that this atom actually occupied the same position in the S1’ pocket of either 3D complex (Fig. 3b,c). As a result the indole heterocycle of AB47 was less buried than the indole ring of SMP289 or the n-pentyl of actinonin in the S1’ pocket and more exposed to the outer side of the enzyme (Fig. 4). To accommodate the indole ring of AB47, a local conformational change of the protein was induced, involving the flip of Tyr167 side chain, and opening up of S1’ pocket (Fig. 4b). Tyr167 is the homolog of Leu125 in the amino acid sequence of *Ec*PDF and of Trp146 in *At*PDF1A. The bulkiness of Trp146 narrows and rigidifies the S1’ pocket of mitochondrial PDFs^26^, a feature used as a unique opportunity for the design of PDFIs specific of bacterial PDFs such as the indole series^28^. Interestingly, SMP289 interacted with *Sa*PDF as AB47 does (Figs 3b,c and 4), but the more pronounced burying of the indole group (Fig. 4b) circumvented the flip of Tyr167 and the induced structural modification of the S1’ pocket. The consequence of AB47-induced S1’ pocket reshaping could explain the better affinity of this compound compared to SMP289 (Tables 1 and 2). Nonetheless, this local structural rearrangement did not increase the overall affinity of the PDF:AB47 complex, since *K*_I*app_ is equal to *K*_I_ (Table 1). Thus, this conformational change was probably small and quick enough to prevent any slow-tight binding effect. Together, the effects observed with this series indicated that hydrogen bonds network beyond P1’ should occur to obtain an inhibitor with strong binding potency.

With a reverse hydroxamate and reverse peptide bond, compound RAS358 (Fig. 1a) was initially designed to probe the hydrogen bond network made with PDF enzymes. This molecule, with an *S* configuration, did not allow the benzyl group to fit the S1’ pocket, placing instead the Boc moiety, whereas the hydroxamate interacted with the catalytic ion (Fig. 3d). Consequently, it did not exhibit good *in vitro* inhibition or antimicrobial activity (Table 1 and ref. 28) and could occupy different binding sites according to the PDF structure. Indeed, RAS358 was previously found to bind at the surface of *At*PDF1B in an unusual binding pocket^45^. In the present work, we showed instead that RAS358 bound *Sa*PDF at the classical ligand binding site with very modest potency and weak slow-tight binding (Fig. 3d; Supplementary Fig. 2d) and with no global or local conformational change (Supplementary Fig. 3). Interestingly, RAS358 made a hydrogen bond with the carbonyl of Gly130, mimicking one interaction observed between tripeptides Fo-Met-Ala-Ser/Arg or actinonin with *Sa*PDF (Figs 3d and 4). We concluded that this bond was not major for a compound to drive strong potency against a PDF. This conclusion is in agreement with previous data obtained with minimal substrates showing that Fo-Met-NH_2_ is the smallest efficient substrate of PDF^59^. Together, biochemical and structural data collected with the *Sa*PDF model suggested that the first hydrogen bond made with the amide of Val71 - or its equivalent in motif I in other PDFs - was crucial for driving both strong potency and slow-tight binding to a PDF inhibitor. In addition, the data obtained with various P1’ side-chains indicated that too bulky groups such as the bromoindole ring should be avoided to allow this hydrogen bond to be made. Based on this analysis, we designed and validated a new series of PDF inhibitors as proof of concept.

### Rational design of new oxazole and oxadiazole PDF inhibitors

The search for new highly potent PDF inhibitors is based on rational drug design aimed at reproducing as much as possible the interaction network which connects peptides to PDFs (Fig. 2b). The reference molecule to date is actinonin (Fig. 3a). Indeed, with a pseudo-peptidic structure, actinonin perfectly mimics a natural substrate, by interacting with PDFs in a manner similar to that observed with peptides, with the exception of the hydroxamate group which replaces the formylated N-terminal methionine^25^,^26^,^45^,^58^,^60^,^61^,^62^,^63^. The high number of interactions between actinonin and PDFs leads to a very high affinity for all tested PDFs, in the low nanomolar range^28^,^31^,^44^,^45^. Moreover, we have shown in a previous work the fundamental role of the hydrogen bond between the first carbonyl of actinonin and the backbone nitrogen of the first hydrophobic residue of motif II (*i.e.*, Val71 in *Sa*PDF, see Fig. 3a) in PDF:ligand interaction, which therefore contributes to high affinity^45^.

Diverse compounds have been synthesized in our laboratory in the past few years including hydroxamate indole derivatives and a reverse hydroxamate peptide (Fig. 3b–d). In our search for new PDFIs, we decided to design new compounds (Fig. 1b–d) containing an oxazole (Fig. 1d) or an oxadiazole^38^ (Fig. 1b,c) as isostere of amide functions. The choice of these two types of molecules was done according to three main criteria aiming to reproduce the main interactions observed in PDF:actinonin complexes as described above: (i) a hydroxamate function coordinating the metal cation (ii) an n-butyl or a cyclopentylmethyl group mimicking the side chain of methionine at P1’, and (iii) a heterocyclic skeleton-link, comprising at least one heteroatom acceptor of hydrogen bond (nitrogen or oxygen) to favor the crucial interaction with the main chain amino group of the first hydrophobic residue of motif I, corresponding to Val 71 in *Sa*PDF (Fig. 3a), Ile 44 in EcPDF and Ile42 in *At*PDF1B. Of note, all potent PDFIs display the actinonin peptide backbone with the one exception of sulfonyl derivatives functionally mimicking the carbonyl at P1’^36^,^64^. To bring novelty to the field while challenging the main missing oxygen donor of AB47, RAS358 and SMP289 for instance, new chemical groups different from carbonyl were worth of being tested.

A rapid overview of the literature reveals that while some heterocycles have been introduced in PDFIs^49^ to disrupt the peptide chain, none of them have been described with an oxazole or an oxadiazole as bioisosteres of the amide bond at P2’ position^65^. This would likely favor the formation of a hydrogen bond with the protein backbone. For instance, pyrrolidine^66^,^67^, 2,5-dihydropyrrole^68^, oxazolidine^69^ or cyclic azaaminoacids^70^, were placed at P2’ position and isoxazole at P2’^71^ or P3’^72^ position. In our approach, in addition, 1,3-oxazole and 1,2,4-oxadiazole heterocycles serve as platform to graft two elements i) the first containing P1’ group of different size to fit the S1’ subsite, and the hydroxamate metal-binding group, and ii) the second, P3’ group more or less flexible supposed to modulate the antibacterial activities. Syntheses, as well as characteristics of all compounds are detailed in the supporting informations.

### *In vitro* assessment of the newly synthesized compounds

In order to assess and compare the inhibition properties of the newly synthesized compounds (Fig. 1), we firstly performed an *in vitro* inhibition assay using *Ec*PDF, *At*PDF1B and *Sa*PDF. We determined for each molecule the concentration required to inhibit 50% of deformylase activity (IC_50_). The values were compared to the reference inhibitor actinonin which showed, as described above, very high inhibition potency against *Sa*PDF, with IC_50_ value of 3 nM (Table 2). This is fully comparable to values obtained against *Ec*PDF and *At*PDF1B (5.3 nM and 7.5 nM respectively, see Table 2), further validating the use of *Sa*PDF to screen new compounds. From this preliminary screening, IC_50_ values obtained with the oxazole series, AT003, AT004, AT007, AT009 and AT010 were similar to those previously observed with RAS358^28^ (Fig. 3a), which displayed moderate inhibition properties against all tested PDFs (Table 2).

The oxadiazole series revealed more interesting properties (Table 2), with a majority of the compounds strongly inhibiting both *Sa*PDF and *Ec*PDF, with IC_50_ values below 30 nM (Table 2). The exceptions corresponded to the compounds showing no “spacer” with aromatic groups grafted at P3’ on the oxadiazole cycle at P2’ (AT001; AT006, AT011, Fig. 1b; Tables 1 and 2), indicating that free rotation of this part is crucial for optimal potency and fit to the active site. These molecules showed weak inhibition against *At*PDF1B (IC_50_ values ranging from 0.077 to 8.2 μM) (Table 2). The very low IC_50_ values obtained with the oxadiazole series suggested that these molecules might form a highly stable complex with PDF and that their mode of binding could fit the slow-onset, tight binding mode exerted by actinonin^28^,^44^,^45^. This process corresponds to a two-step mechanism where the initial complex (EI) is formed and then slowly converted to a more stable state (EI*) (Supplementary Fig. 4a). We measured *K*_I_ and *K*_I*app_ values of the most interesting new synthesized molecules to determine their binding mode. The overall inhibition constant *K*_I*app_ of *Sa*PDF obtained with AT002, AT018, AT019 and AT020 was significantly lower compared to the initial value *K*_I_, *i.e.*, *K*_I_/*K*_I*app_ > 1 (Table 1), which was indicative of a two-step tight-binding process. In contrast, for all other molecules of the oxadiazole series *K*_I_ was equal to *K*_I*app_ (Table 1), indicating a one-step inhibitory process. The oxadiazole compounds showed the same behavior with type 1 PDFs such as *Ec*PDF, with the exception of AT008 and AT015 which behaved as tight-inhibitors only for *Ec*PDF (Table 1). Moreover, AT019 and AT020 were of particular interest because *K*_I*app_ value for *Ec*PDF was especially very low, of the order of 1 nM, and *K*_I_/*K*_I*app_ was equal to 69 and 42 respectively, approaching the value obtained with actinonin (Table 1 and Supplementary Fig. 4). The stronger potency of AT019 compared to AT015, or AT020 compared to AT016, appeared to be the only consequence of a bulkier and more hydrophobic group in P1’ position (a cyclopentylmethyl instead of an aliphatic chain, see Fig. 1b,c). Interestingly, PDFI which have reached clinical trials including BB-83698^73^ and GSK1322322^74^,^75^ also contain a cyclopentyl group at P1’.

These data showed that some compounds of the diazole series are almost as potent as actinonin and that the conclusions drawn from both the biochemical information obtained with the *Sa*PDF model can be used to design *de novo* new potent PDFIs. In this respect, it was important to check whether the interaction network was as expected from the initial design and validate that this hydrogen bond is crucial for strong potency.

### Structural basis of PDF inhibition by the oxadiazole series

As stated above, the new compounds were rationally designed with the aim to mimic the crucial interactions between ligands and PDF that confer a very high inhibitory potency. In order to describe interactions at atomic level between these new molecules and *Sa*PDF we conducted soaking experiments with crystals of *Sa*PDF apo-protein with the most promising oxadiazoles. We were able to solve high resolution structures (Supplementary Table 3) of *Sa*PDF bound to AT002, AT018, AT019 and AT020. The P3’ group of these oxadiazoles could not be entirely modeled (Supplementary Fig. 2c) since it was partially flexible and, in agreement with the aforementioned data showing that free rotation around this axis was required to get optimal potency. Nonetheless, the hydroxamate, P1’ and P2’ groups of these compounds were well defined and could be built satisfactorily (Supplementary Fig. 2c). Binding of compounds to the protein did not induce any strong conformational changes of protein (Supplementary Fig. 4).

Similarly to actinonin, the hydroxamate group in the oxadiazole series was linked to the protein through hydrogen bonds, especially with Glu175, and coordinated the catalytic metal cation (Fig. 3e,f). In addition, the aliphatic P1’ group fitted perfectly into the hydrophobic S1’ pocket, which in turn did not undergo any conformational change (Fig. 3e,f). Finally, since all oxadiazole molecules do not display the pseudo-peptidic structure of actinonin, AT002, AT018, AT019 and AT020 did not make any hydrogen bonds with the two flanking β-sheets of *Sa*PDF accordingly (Fig. 3e,f). However, the oxygen of the oxadiazole cycle which was designed to mimic the carbonyl of the first peptide bond of actinonin was hydrogen bonded with the backbone nitrogen of Val71 as expected (Fig. 3e,f). Compared to actinonin or AT002 and AT018, the cyclopentylmethyl P1’ group of AT019 and AT020 apparently filled into the S1’ pocket in a similar fashion, roughly mediated by hydrophobic interactions with residues that build the S1’ pocket (see Fig. 3e,f). However, this cyclic P1’ group presumably occupies a significantly higher volume than a simple aliphatic chain (Fig. 3e,f, right panels), increasing the attractive van der Waals interactions as a result. This explains why AT019 and AT020 are more potent than AT015 and AT016, respectively (Tables 1 and 2).

### Validating the antibacterial activity of the oxadiazole series *in cellulo*

Several studies have shown that, depending on the nature of the compounds, two important factors, which sustain intracellular accumulation, contribute to ensure the effectiveness of a drug *in vivo*: cell permeability and drug efflux outside the cell^76^,^77^,^78^. For instance, actinonin is known to easily penetrate into Gram-positive bacteria thanks to its high hydrophylic nature but in many bacteria it is efficiently detoxified by AcrAB-TolC efflux pumps^31^,^37^. In contrast, hydrophobic PDF inhibitors were reported to display low cellular uptake due to low permeability of the outer membrane in Gram-negative bacteria^37^.

The antibacterial potency of all synthesized compounds was tested *in vivo,* by measuring the respective minimum inhibitory concentrations (MIC). Several pathogen strains representative of Gram positive and Gram negative bacteria were used, including two *E. coli* strains *i.e*., K37 TolC- and AG100A in which *tolC* or *acrAB* efflux pump genes were deleted respectively (see Table 3 and Supplementary Table 3). Moreover, the most *in vitro* effective oxadiazole inhibitors were tested on an *Enterobacter aerogenes* multi-drug resistant (MDR) clinical isolate overexpressing AcrAB-TolC efflux pumps and its *acrAB*^-^ and *tol*C^-^ derivatives strains (Table 4). All analyses were performed in the absence or the presence of polymyxin B nonapeptide (PMBN). This molecule has been previously described to increase the outer membrane permeability of Gram negative bacteria and facilitate the permeation of various drugs^37^,^79^,^80^. Compared to actinonin and the indole series, the tested new compounds alone showed no activity towards the *E. coli* AG100 wild type strain (Table 3). Moreover, most of the new compounds, unlike actinonin and the indole series, did not display a better antibacterial activity against *E. coli tolC-* and *acrAB-* strains (Table 3 and Supplementary Table 3). In contrast, when PMBN was present, a noticeable increase of antibacterial activity was observed indicating that the limiting step for these compounds could be their low permeation rate through the outer membrane (Table 3). Interestingly, in the case of the two best inhibitors AT019 and AT020, an increase of the antibacterial activity was obtained against *E. coli acrAB-.* When the best compounds were assayed on the *E. aerogenes* strains, the same results were obtained with the wild type strain and its *AcrAB-* or *tolC-* derivatives (Tables 3 and 4). When comparing the effect of efflux expression on the antibacterial activity of these compounds (in the presence of PMBN to avoid any permeation limitation) the ratio “MIC in AcrAB+/MIC in AcrAB-“ were quite similar for *E. coli* and *E. aerogenes*. Moreover, the AcrAB pump seemed to be unable to discriminate between AT015 and AT019, which contain both a benzofuran moiety and differ only by the P2’ group (Fig. 1b,c), since the ratio was similar. Interestingly, these activity levels were similar to that obtained with actinonin (Table 3 and Supplementary Table 3).

It is noteworthy that under the same conditions, usual antibiotics such as ceftazidime or ciprofloxacin displayed a limited antibacterial activity (64 μg/mL for ceftazidime and 4 μg/mL for ciprofloxacin, respectively) whereas AT019 and AT020 exhibited significant low MICs (1–2 μg/mL) against the efflux derivative MDR strains (Table 4). Moreover, the antibacterial activity measured was quite similar to chloramphenicol, a drug that is banished for human use due to its toxicity, underlining the potentiality of these oxadiazole compounds.

## Conclusions

Altogether, the comparison of the different structures of *Sa*PDF:PDFI complexes allow to highlight the significance of the oxadiazole group for efficient PDF binding and inhibition. In this context, there is very strong correlation between slow tight-binding potency - as assessed by *K*_I_/*K*_I*app_ values higher than one - and the creation of a hydrogen bond between the backbone NH of Val71 and an oxygen of the ligand as found in actinonin and in oxadiazole series, and unlike the indole series (Fig. 1 and 3, Table 1). Optimization of this hydrogen bonding by narrowing and aligning the amide and oxygen moieties needs a proper chemical context as witnessed by the variation of the values in the oxadiazole series. It is also interesting that with its reverse peptide structure RAS358 does make a hydrogen bond but with the peptide carbonyl of Gly130 similarly to the best compound, actinonin. However, this is not enough to cause any significant increase of the binding constant. In this context, it is likely that the occurrence of two consecutive H-bonds ensured by actinonin and derivatives, allow them to act synergistically to lock the interaction of the inhibitor with the protein backbone and induce tighter binding as a result.

By using *Sa*PDF as model platform, we could get several take-home lessons. First of all, the hydrogen bonding network of the first peptide bond is crucial to get potent PDFIs exhibiting slow-tight binding. A strong *K*_I_/*K*_I_^*^value *i.e.*, strong potency of a PDFI actually witnesses the creation at least of the hydrogen bond between NH of Ile44 (*Ec*PDF), Ile42 (*At*PDF1B) or Val71 (*Sa*PDF) and a suitable oxygen acceptor of the compound. The perfect alignment usually needs an additional hydrogen bond between the amide of the PDFI and the carbonyl of Gly130 (*Sa*PDF) or Gly 89 (*Ec*PDF) or Gly90 (*At*PDF1B). Second, it is feasible to get out of the peptide backbone while still maintaining the minimal hydrogen bond network as shown here with the case of the oxadiazole series. Third, the cyclopentyl at P1’ appears to somewhat potentiate this effect compared to an n-butyl group. Fourth, the nature of the P3’ chain drives the final pharmacological properties of the compound. In this respect, an important concern is the drug permeation across the bacterial membrane: the increase of antibacterial activity obtained with PMBN paves the way for the development of new side chains at P3’ or “cargo” peptides ensuring the *in vivo* translocation inside bacterial cells.

## Materials and Methods

### Materials

All solvents and chemicals were purchased from SDS and Aldrich, respectively. DMF, MeOH and CH_3_CN were dried using standard. ^1^H NMR and ^13^C NMR spectra were recorded on Bruker ARX-250 and Bruker Avance-500 spectrometers, respectively, and chemical shifts were reported in ppm downfield from TMS. IR spectra were obtained with a Perkin-Elmer Spectrum One FT-IR spectrometer equivuipped with a MIRacle^TM^ single reflection horizontal ATR unit (germanium crystal). Electrospray ionization (ESI) mass spectrometry analyses and HRMS were obtained using Thermo Finnigan LCQ Advantage spectrometer. The elemental analyses were carried out by the mass spectrometry and microanalysis services in Gif-sur-Yvette (CNRS).

### Cloning, expression and purification of *S. agalactiae* PDF

The nucleotides encoding PDF were amplified from *Streptococcus agalactiae* serotype III NEM16 genomic DNA (UniProtKB accession number Q8E378) and cloned into pET16b vector (Novagen) between NcoI and XhoI restriction sites such that the recombinant protein contains all residues of the 204 amino acids mature protein and an Ala in second position instead of Ser (S2A) as a cloning artifact. *E. coli* strain Rosetta 2(DE3)pLysS (Novagen) was transformed with the resultant plasmid for protein production. Culture in 1L-2YT medium supplemented by 50 μg/mL ampicillin and 34 μg/mL chloramphenicol was grown shaking at 18 °C to an OD_600_ of approximately 0.8 and induced overnight at 18 °C with 0.5 mM IPTG. Cells were harvested by centrifugation at 8000 rpm and 4 °C for 20 min and then resuspended in 40 mL of lysis buffer (50 mM HEPES-NaOH pH7.5, 3 mM NiCl_2_). Lysis was performed by sonication and resulting lysate was cleared by centrifugation at 14,000 rpm and 4 °C for 30 min. Supernatant containing approximately 50% of total expressed protein was applied on a Q-Sepharose column (GE Healthcare Life Sciences) pre-equilibrated in buffer A (50 mM Hepes-NaOH pH7.5, 100 μM NiCl_2_). Elution was performed using a 20 column volumes linear gradient from 0 to 100% of buffer B (50 mM Hepes-NaOH pH7.5, 1 M NaCl, 100 μM NiCl_2_). Protein was further purified by gel filtration, using a Superdex 75 column (GE Healthcare Life Sciences). Elution was performed with buffer C (50 mM Hepes-NaOH pH7.5, 0.1 M NaCl, 100 μM NiCl_2_). All purification steps were performed at 4 °C and analyzed by SDS-PAGE on 12.5% acrylamide gels. The purified protein was concentrated on Amicon Ultra centrifugal filters and protein concentration was estimated from the calculated extinction coefficient at 280 nm (8,940 M^−1^cm^−1^). The resulting *Sa*PDF preparation was frozen at −80 °C or diluted 2-fold in 100% glycerol and stored at −20 °C, for crystallization or enzymatic purposes respectively.

*Escherichia coli* and *Arabidopsis thaliana* PDF (*Ec*PDF and *At*PDF1B, respectively) were expressed and purified as previously described^16^,^45^,^81^.

### Analytical size exclusion chromatography

Estimation of *Sa*PDF molecular weight was performed by analyzing protein by gel filtration on an analytical Superdex 75 HR 10/30 column (GE Healthcare Life Sciences) equilibrated in buffer C and calibrated with molecular weight markers (Blue Dextran 2000, 2000 kDa; bovine serum albumin, 67 kDa; ovalbumin, 43 kDa; chymotrypsinogen A, 25 kDa; ribonuclease A, 13.7 kDa; GE Healthcare Life Sciences). Sample with a protein concentration of 2 mg/mL was centrifuged (10 min at 14000 rpm and 4 °C) and 100 μL was loaded onto the column. Elution was performed in buffer C at a constant flow rate (0.5 mL/min). Molecular weight of *Sa*PDF was estimated by integration of the chromatogram performed using Unicorm software (GE Healthcare Life Sciences).

### Crystallization and structure solution

Crystallization conditions of *Sa*PDF in its apo form were screened by a robot by using commercial kits. Two slightly different crystallization conditions were obtained and manually optimized at 18–20 °C. Hanging drops were formed by mixing 1 μL of 14–17 mg/mL protein preparation with 1 μL of reservoir solution containing 5–15% PEG-8000, 0.2–0.5 M zinc acetate and 0.1 M imidazole pH7.5 or 0.1 M sodium cacodylate pH6.5. Stable crystals of apo-*Sa*PDF were soaked by adding ligands to the drop to a final concentration of 16–32 mM. Prior to data collection, crystals were flash cooled in liquid nitrogen following a stepwise treatment with the crystallization solution containing increasing amounts of glycerol up to a final concentration of 20% (v/v).

X-ray diffraction data were collected at 100 K at the European Synchrotron Radiation Facility (Grenoble, France) on station ID14-1, ID14-4 and ID23-2, and at SOLEIL (Gif-sur-Yvette, France) on station PROXIMA1 beamline. In each case, a single crystal was used to collect a complete dataset. All crystals belong to the space group P2_1_2_1_2_1_ with equivalent cell parameters and one molecule per asymmetric unit. Data were processed and scaled with XDS software^82^ (Supplementary Table 2). Initial phases were obtained by molecular replacement with Phaser^83^ followed by a rigid-body refinement by CNS^84^ using the *Streptococcus pneumoniae* structure (PDB code 2AIE)^85^ as the starting model. Structures of ligand-bound proteins were solved by rigid-body refinement in REFMAC^86^ using the apo-*Sa*PDF structure. Multiple cycles of refinement and model building were performed with the programs REFMAC and TURBO-FRODO^87^ (Supplementary Table 2). In each case, 5% of the data was excluded for calculation of R_free_. Quality control of the 12 final models was performed with PROCHECK^88^.

### Enzymology

Enzymatic activity was measured by a spectrophotometric assay where peptide deformylase activity is coupled to that of formate dehydrogenase^89^. The formate released by the deformylation reaction is used by FDH to convert NAD in NADH, the production of which being measured over time at 340 nm and controlled temperature (37 °C). The 200 μL reaction mixture contained 50 mM HEPES-NaOH pH7.5, 12 mM NAD^+^, 4.5 U/mL FDH, 1 mM NiCl_2_ and PDF enzyme (either 40 nM *Sa*PDF or 10 nM *Ec*PDF or 100 nM *At*PDF1B) previously diluted in presence of 0.1 mg/mL BSA. The reaction was started by addition of 1–6 mM Fo-Met-Ala-Ser. Kinetics parameters (*k*_cat_, *K*_m_) were derived from iterative non-linear least square fits using the Michaelis-menten equation based on the experimental data (Sigma-Plot, Kinetics module).

Determination of IC_50_ and *K*_I*app_ values was performed by incubating 10 min at 37 °C the reaction mixture containing increasing concentrations of each compound, and reaction was then started by addition of 2 mM Fo-Met-Ala-Ser. IC_50_ values were obtained from the concentration-responses plots (*i.e.*, [compound] against PDF residual activity). 1/*K*_I*app_ is the slope of the v_0_/v_i_ curve in function of compound concentration, where v_0_ and v_i_ are the initial reaction rates in absence and presence of compound, respectively.

Determination of *K*_I_ values was performed with a slightly modified protocol: reaction was started by addition of enzyme instead of Fo-Met-Ala-Ser, the latter being directly added to the mixture containing increasing concentrations of each compound. No pre-incubation of enzyme in presence of compound was done. 1/*K*_I_ is the slope of the v_0_/v_i_ curve in function of compound concentration, where v_0_ and v_i_ are the initial reaction rates in absence and presence of compound respectively.

### Bacterial strains and growth

Two *E. coli* strains, one *B. subtilis* strain, one *S. aureus* strain, one *E. faecalis* strain, one *S. agalactiae* strain and three *Enterobacter aerogenes* strains previously described were used. Briefly, AG100 was an *E. coli* wild type strain and AG100A its *acr*AB^-^ derivative^37^. EA289 was a Kan^s^ derivative of an *E. aerogenes* multi-drug resistant (MDR) clinical isolate overexpressing AcrAB-TolC efflux pumps, and EA294 and EA298 its *acr*AB^-^ and *tol*C^-^ derivatives respectively^38^. Strains were routinely grown at 37 °C on Luria-Bertani (LB) agar or in Mueller-Hinton II broth (MH), supplemented with kanamycine (50 μg.mL^−1^) for AG100A, EA294 and EA298.

### Susceptibility determinations

MIC values of antibiotics were determined by the microdilution method in liquid MH media in microplates in the absence or presence of chemosensitizers/modulators (*e.g.* PMBN, PAβN), as previously described^90^. Permeabilization, blocking of efflux pumps, and isolates with different levels of porin or efflux expression were used to assess the involvement of the membrane barrier in antibiotic activity^37^.

Susceptibilities were determined in 96-wells microplates with an inoculum of 2 × 10^5^ cfu in 200 μL of MH broth containing two-fold serial dilutions of each antibiotics or compounds. Molecules were tested alone or in combination with other molecules in equimolar ratio. MICs were realized in the absence and in the presence of a membrane permeabilizer, the polymyxine B nonapeptide (PMBN) used at 51.2 mg/L (1/5 or 1/10 of its direct MIC previously determined). Actinonin and ciprofloxacin were used as internal antibiotic reference. The MIC was defined as the lowest concentration of each compound for which no visible growth was observed after 18 h of incubation at 37 °C. Each test was systematically performed in triplicate. The average of three independent assays is presented in μg/mL in the respective tables.

### Synthesis of PDFI

Synthesis of all new compounds is reported in Supporting information.

## Additional Information

**How to cite this article**: Fieulaine, S. *et al.* A unique peptide deformylase platform to rationally design and challenge novel active compounds. *Sci. Rep.* **6**, 35429; doi: 10.1038/srep35429 (2016).

## Supplementary Material

Supplementary Information

## Figures and Tables

**Table 1:** *In vitro* inhibition constants of PDFIs against PDFs from *S. agalactiae*, *E. coli* and *A. thaliana*.

PDF-In	*Sa*PDF			*Ec*PDF			*At*PDF1B		
	*K*_I_ (nM)	*K*_I*app_ (nM)	*K*_I_/*K*_I*app_	*K*_I_ (nM)	*K*_I*app_ (nM)	*K*_I_/*K*_I*app_	*K*_I_ (nM)	*K*_I*app_ (nM)	*K*_I_/*K*_I*app_
Actinonin	64 ± 5	1.0 ± 0.1	**64**	185 ± 15	1.7 ± 0.5	**109**	82 ± 10	3.5 ± 0.3	**23**
*Indole serie*									
AB47	62 ± 3	73 ± 5	1	47 ± 2	39 ± 2	1	455 ± 89	400 ± 35	1
SMP195	285 ± 18	250 ± 17	1	29 ± 4	20 ± 3	1	909 ± 118	833 ± 53	1
SMP289	909 ± 64	714 ± 72	1	769 ± 30	625 ± 75	1	1000 ± 78	1,428 ± 126	1
*Peptide serie*									
RAS358	1428 ± 125	454 ± 29	**3**	833 ± 30	164 ± 40	**5**	2000 ± 150	400 ± 37	**5**
*Oxadiazole serie*									
AT002	13 ± 1	4.0 ± 0.5	**3**	31 ± 7	5 ± 2	**6**	58 ± 4	53 ± 3	1
AT008	75 ± 4	81 ± 5	1	80 ± 10	18 ± 3	**4**	909 ± 78	666 ± 29	1
AT011	400 ± 20	430 ± 39	1	—	333 ± 13	—	—	5,000 ± 500	—
AT012	—	400 ± 35	—	—	70 ± 8	—	—	625 ± 30	—
AT013	70 ± 5	51 ± 3	1	73 ± 12	80 ± 10	1	—	303 ± 12	—
AT014	84 ± 6	65 ± 10	1	—	66 ± 3	—	—	909 ± 32	—
AT015	166 ± 12	122 ± 13	1	35 ± 5	10 ± 2	**3**	—	1,100 ± 115	—
AT016	67 ± 7	55 ± 5	1	25 ± 3	26 ± 5	1	—	—	—
AT017	—	85 ± 8	—	—	41 ± 3	—	—	—	—
AT018	38 ± 6	18 ± 1	**2**	49 ± 3	12 ± 1	**4**	1111 ± 100	833 ± 10	1
AT021	—	200 ± 10	—	—	76 ± 5	—	—	~10 μM	—
AT019	65 ± 6	7.5 ± 2	**9**	76 ± 4	1.1 ± 0.3	**69**	263 ± 26	54 ± 2	**5**
AT020	63 ± 3	8.5 ± 3	**7**	80 ± 3	1.9 ± 0.2	**42**	238 ± 30	123 ± 5	**2**

Prior to kinetic analysis for determination of the *K*_I*app_ value, each PDFI was incubated at the final concentration in the presence of the studied enzyme set during 10 min at 37 °C. The kinetic assay was initiated by the addition of a small volume of the substrate. For determination of *K*_I_, PDFIs were not pre-incubated with the enzyme, the kinetic assay being initiated by the addition of the enzyme.

The enzyme concentrations used in the assay were 40 nM, 10 nM and 100 nM for *Sa*PDF, *Ec*PDF and *At*PDF1B, respectively.

**Table 2:** *In vitro* inhibitory activity of PDFIs against PDFs from *S. agalactiae*, *E. coli* and *A. thaliana*.

PDF-In	IC_50_ (nM)		
	*Sa*PDF	*Ec*PDF	*At*PDF1B
Actinonin	3.0 ± 0.9	5.3 ± 0.5	7.5 ± 1.8
*Oxadiazole serie*			
AT001	—	160	—
AT002	3.5 ± 0.8	4 ± 1	—
AT006	—	155	—
AT008	25 ± 2	14 ± 2	—
AT011	128 ± 32	215 ± 17	6,000 ± 1,500
AT012	67 ± 5	27 ± 3	460 ± 22
AT013	11 ± 1	78 ± 11	500 ± 15
AT014	16 ± 2	25 ± 4	550 ± 25
AT015	20 ± 3	17 ± 2	300 ± 12
AT016	10 ± 2	10 ± 1	—
AT017	23 ± 4	30 ± 8	—
AT018	20 ± 1	20 ± 2	650 ± 20
AT021	211 ± 10	75 ± 2	8200 ± 1000
AT019	7 ± 1	6 ± 1	77 ± 5
AT020	12 ± 3	7 ± 2	119 ± 10
*Oxazole serie*			
AT003	—	190	—
AT004	—	110	—
AT007	—	740	—
AT009	—	330	—
AT010	—	420	—
*Indole serie*			
AB47	63 ± 6	37 ± 5	360 ± 31
SMP195	110 ± 10	17 ± 4	400 ± 33
SMP289	360 ± 21	480 ± 30	703 ± 47
*Peptide serie*			
RAS358	150 ± 12	110 ± 10	251 ± 15

The enzyme concentrations used in the assay were 40 nM, 10 nM and 100 nM for *Sa*PDF, *Ec*PDF and *At*PDF1B, respectively. All measurements were achieved in the presence of 2 mM Fo-Met-Ala-Ser.

**Table 3:** Minimal Inhibitory Concentrations of oxadiazole compounds on *E. coli* strains.

Compounds	MIC (μg/mL)			
	AG100		AG100A	
PMBN	−	+	−	+
Actinonin	128	4	1	0.25
AB47	>64	64	64	32
SMP289	>64	8	>64	4
AT001	>64	>64	>64	32
AT002	>64	64	>64	32
AT003	>64	64	>64	16
AT004	>64	>64	>64	32
AT006	>64	64	>64	32
AT007	>64	>64	>64	>64
AT008	>64	>64	>64	16
AT009	>64	>64	>64	>64
AT010	>64	>64	>64	>64
AT011	>64	8	32	4
AT012	>64	32	64	8
AT013	>64	16	32	4
AT014	>64	64	>64	64
AT015	>64	16	32	**2**
AT016	>64	64	64	8
AT017	>64	64	64	8
AT018	>64	64	>64	16
AT019	>64	**2**	16	**0.5**
AT020	>64	**8**	16	**4**

AG100, parental strain expressing AcrAB efflux pump, AG100A, the AcrAB- derivative.

The values corresponding to the average of three independent assays obtained in the absence or in the presence of PMBN used at 1/5 of its respective MIC for each tested strain are presented.

**Table 4:** Minimal Inhibitory Concentration of the more active compounds on *E. aerogenes* MDR strains: concentrations are indicated in μg/mL.

Compounds	Ea289		Ea294		Ea298	
	—	+PMBN	—	+PMBN	—	+PMBN
AT011	>128	64	128	16	128	16
AT013	>128	64	>128	16	128	16
AT015	>128	128	>128	16	128	8
AT019	>128	16	128	**2**	64	**1**
AT020	>128	32	128	8	128	**2**
Actinonin	>64	32	64	4	32	1
Ceftazidim	>128	64	>128	64	>128	64
Ciprofloxacin	64	64	16	8	8	4
Chloramphenicol	>128	8	32	4	32	4

*E. aerogenes* strains^37^: Ea289, MDR strain; Ea294, Ea289 derivative AcrAB-; Ea298, Ea289 derivative tolC-. The values corresponding to the average of three independent assays obtained in the absence or in the presence of PMBN used at 1/5 of its respective MIC for each tested strain are presented.
