# Corrigendum: A unique peptide deformylase platform to rationally design and challenge novel active compounds

**DOI:** 10.1038/srep39365

**Published:** 2017-01-11

**Authors:** Sonia Fieulaine, Rodolphe Alves de Sousa, Laure Maigre, Karim Hamiche, Mickael Alimi, Jean-Michel Bolla, Abbass Taleb, Alexis Denis, Jean-Marie Pagès, Isabelle Artaud, Thierry Meinnel, Carmela Giglione

Scientific Reports 6: Article number: 35429; 10.1038/srep35429 published online: 10 20 2016 updated: 01 11 2017.

The Supplementary Information file originally published with this Article contained incorrect Figures for the ‘NMR spectra of all new compounds’. This error has now been corrected in the Supplementary Information file that accompanies the Article.

