# Supplementary material for: A unique peptide deformylase platform to rationally design and challenge novel active compounds
Source: Sci Rep. 2016 Oct 20;6:35429. doi: 10.1038/srep35429 (PMC5071857; doi:10.1038/srep35429)

A unique peptide deformylase platform to rationally design and challenge novel active compounds

Sonia Fieulaine[1], Rodolphe Alves de Sousa[2], Laure Maigre[3], Karim Hamiche[1], Mickael Alimi[2], Jean-Michel Bolla[3], Abbass Taleb[2], Alexis Denis[4], Jean-Marie Pagès[3], Isabelle Artaud[2], Thierry Meinnel[1,*] and Carmela Giglione[1,*]

Supplementary Information

Content

Supplementary Figure 1

Supplementary Figure 2

Supplementary Figure 3

Supplementary Figure 4

Supplementary Figure legends

Supplementary Table 1

Supplementary Table 2

Supplementary Table 3

Supplementary Methods

- Chemical synthesis
- NMR spectra

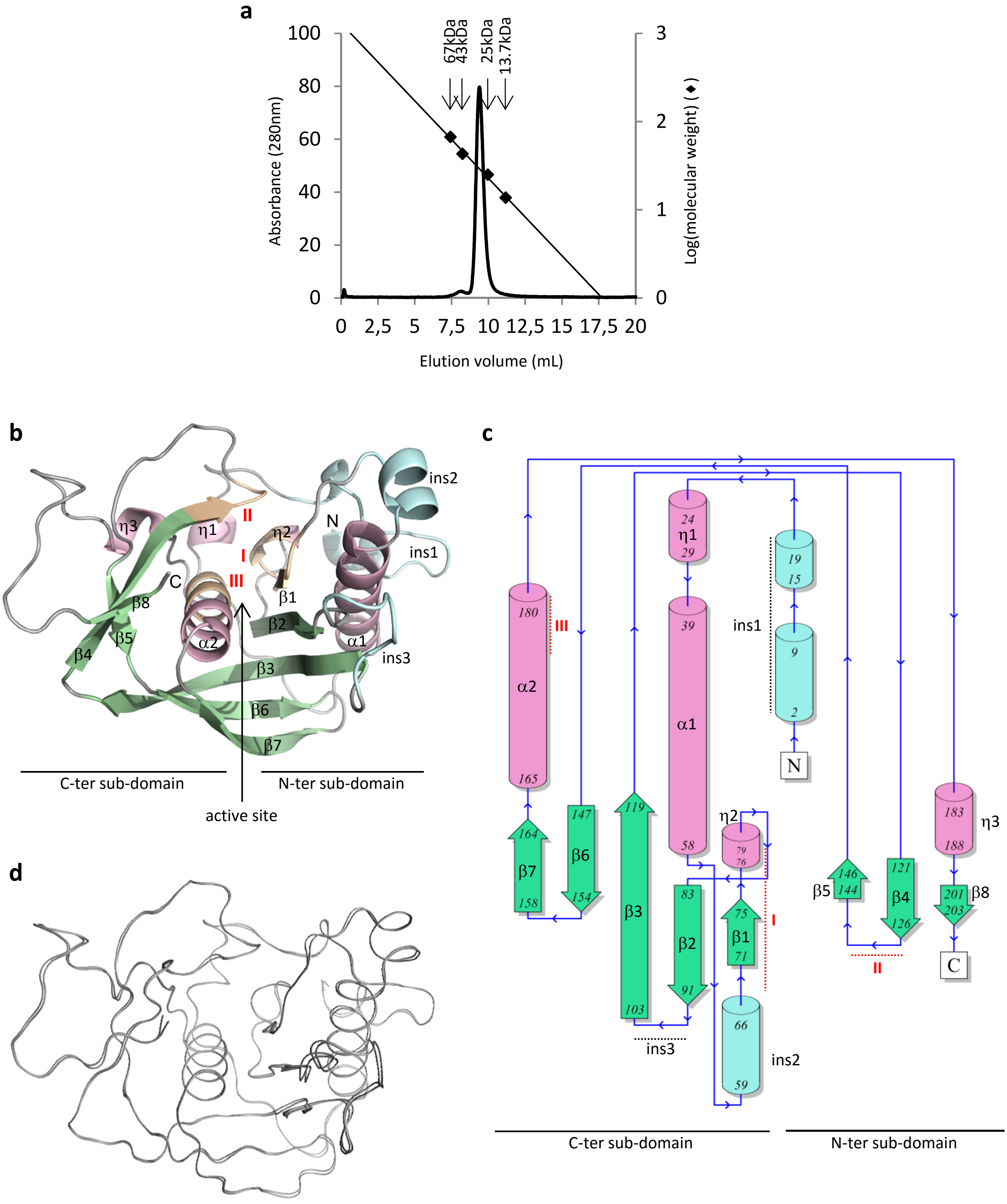

Supplementary Figure 1

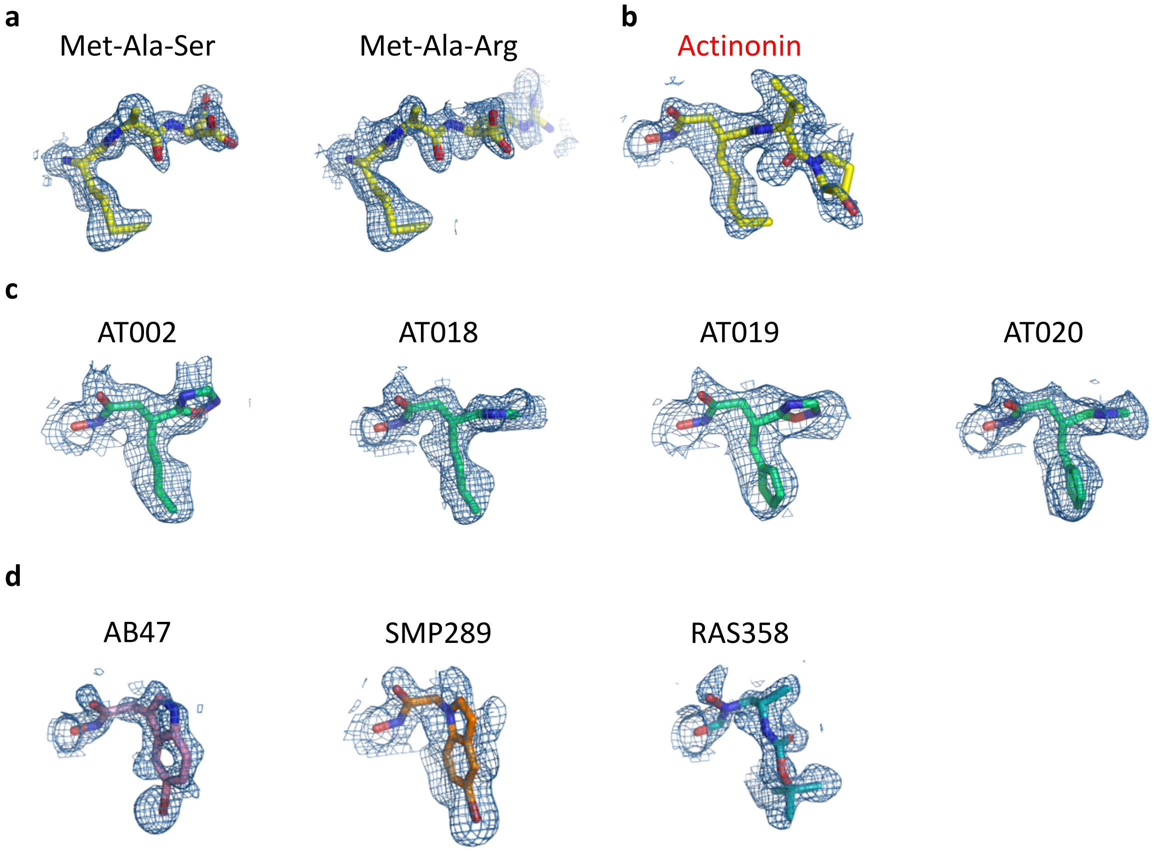

Supplementary Figure 2

a

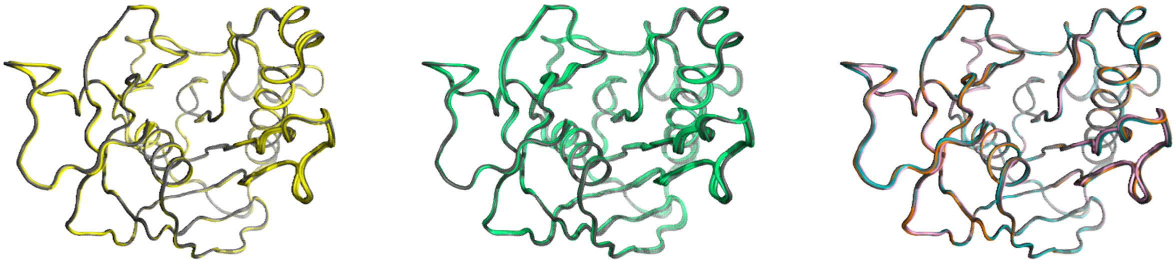

Supplementary Figure 3

a

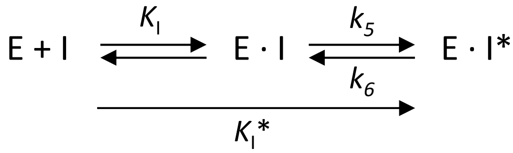

b ***Sa*PDF2a**

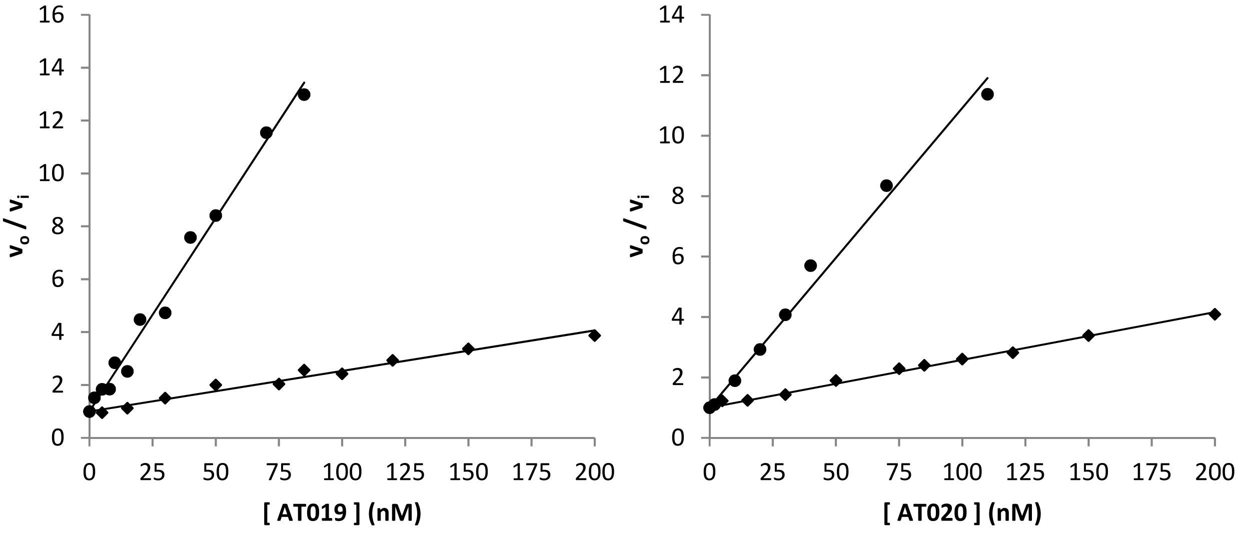

c ***Ec*PDF**

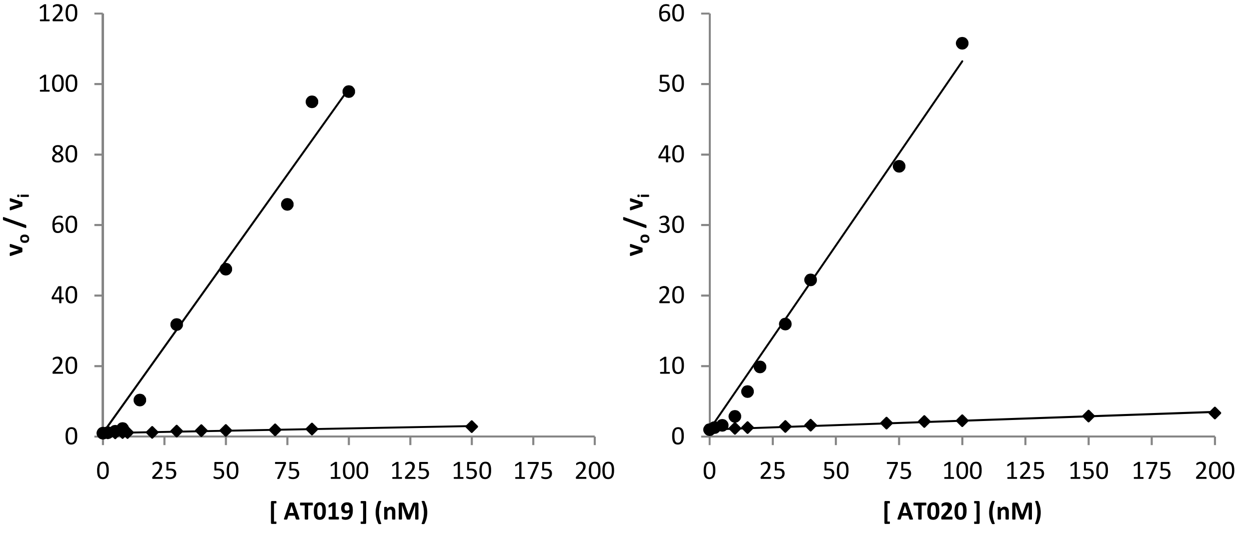

Supplementary Figure 4

Supplementary Figure legends

Supplementary Figure 1. 3D structure of apo *Sa*PDF.

(a) Size-exclusion chromatographic analysis of apo *Sa*PDF. The elution profile on Superdex 75 is shown, as well as the calibration plot obtained by column calibration with standard globular proteins ranging in size from 13.7 to 67 kDa.

(b) *Sa*PDF ribbon diagram with α and 3_{10} helices in pink, β strands in green and insertions in light blue, showing the two sub-domains encircling the active site. The three consensus motifs I, II and III are colored in light orange.

(c) Topology cartoon of *Sa*PDF, with the same color code as panel b. PDB sum (http://www.ebi.ac.uk/thornton-srv/databases/pdbsum/) was used.

(d) Superimposition of the two apo *Sa*PDF structures obtained from the two crystal forms (see Materials and Methods), in the same view than panel b.

Supplementary Figure 2. Identification of PDFIs in the ligand binding site of *Sa*PDF.

Electronic densities of **(a)** tripeptides Met-Ala-Ser and Met-Ala-Arg, **(b)** actinonin, **(c)** AT002, AT018, AT019 and AT020, **(d)** AB47, SMP289 and RAS358. PDFIs are drawn in sticks and are shown in their $F_O - F_C$ electron density omit maps contoured at 2σ.

Supplementary Figure 3. Global conformation of *Sa*PDF in complex with PDFIs.

(a) Superimposition between the two models of apo protein (in gray) with protein in complex with Met-Ala-Ser, Met-Ala-Arg and actinonin (in yellow) (*left*), in complex with AT002, AT018, AT019 and AT020 (*middle*), and AB47, SMP289 and RAS358 (*right*).

Supplementary Figure 4. Slow tight-binding inhibition of PDFs by AT019 and AT020.

(a) Slow tight-binding inhibition is a two-step mechanism, involving a tightening of the initial enzyme-inhibitor complex (E·I) to form a more stable complex (E·I*). PDFIs binding was assessed by measuring inhibition constants K_I*app and K_I through two different protocols. In

brief, determination of K_I*app values was done by incubating the PDFI of interest at the final concentration in the presence of the studied enzyme, during 10 min at 37°C. The kinetic assay was then initiated by the addition of a small volume of the substrate Fo-Met-Ala-Ser. 1/ K_I*app is the slope of the v_0/v_i curve in function of compound concentration (•), where v_0 and v_i are the initial reaction rates in absence and presence of compound, respectively. Determination of K_I values was done without preliminary incubation of PDFI with the enzyme, the kinetic assay being initiated by the addition of the enzyme. In this case, 1/ K_I is the slope of the v_0/v_i curve in function of compound concentration (♦), where v_0 and v_i are the initial reaction rates in absence and presence of compound respectively. See Materials and methods for more details.

(b) Inhibition of *Sa*PDF by AT019 and AT020.

(c) Inhibition of *Ec*PDF by AT019 and AT020.

Supplementary Table 1. Catalytic properties of *Streptococcus agalactiae* PDF (*Sa*PDF) and other bacterial PDFs

Enzyme	k_{cat} (s^{-1})	K_m (mM)	k_{cat} / K_m ($M^{-1}s^{-1}$)
Ni-*Sa*PDF2a	50 ± 3	1.2 ± 0.1	41,667
Ni-*Ec*PDF[a]	210 ± 13	3.9 ± 0.6	53,846
Ni-*Tt*PDF[a]	27 ± 3	2.3 ± 0.5	11,739
Ni-*Bst*PDF2[a]	1007 ± 191	4.1 ± 1.2	245,610

Kinetic constants of *Sa*PDF purified in presence of nickel (Ni-*Sa*PD) were determined using a coupled assay as indicated in Materials and Methods with substrate Fo-Met-Ala-Ser, in the presence of 40nM enzyme and 1mM $NiCl_2$ at 37°C.

[a] Data for nickel-substituted PDFs from *E. coli*, *Thermus thermophilus* and *Bacillus stearothermophilus* (Ni-*Ec*PDF, Ni-*Tt*PDF, and *Bs*PDF2) were from Ref.[54].

Supplementary Table 2. Data collection and refinement statistics

**Ligand**	**no ligand**	**no ligand**	**Met-Ala-Ser**	**Met-Ala-Arg**	**actinonin**	**AT002**	**AT018**	**AT019**	**AT020**	**AB47**	**SMP289**	**RAS358**
**Crystal form**	imidazole	cacodylate	cacodylate	cacodylate	cacodylate	imidazole	imidazole	imidazole	imidazole	imidazole	imidazole	imidazole
**Data collection**												
Space group	P$2_12_12_1$	P$2_12_12_1$	P$2_12_12_1$	P$2_12_12_1$	P$2_12_12_1$	P$2_12_12_1$	P$2_12_12_1$	P$2_12_12_1$	P$2_12_12_1$	P$2_12_12_1$	P$2_12_12_1$	P$2_12_12_1$
Unit cell parameters (Å)	a = 41.3<br>b = 65.5<br>c = 88.7	a = 41.4<br>b = 66.0<br>c = 89.4	a = 41.1<br>b = 65.6<br>c = 88.8	a = 41.2<br>b = 65.4<br>c = 88.5	a = 40.8<br>b = 66.2<br>c = 88.9	a = 41.3<br>b = 65.5<br>c = 88.9	a = 41.1<br>b = 65.4<br>c = 88.8	a = 41.1<br>b = 65.7<br>c = 88.5	a = 41.0<br>b = 65.27<br>c = 88.5	a = 40.9<br>b = 66.3<br>c = 88.3	a = 41.5<br>b = 66.0<br>c = 89.3	a = 41.5<br>b = 65.8<br>c = 88.6
Resolution (Å)	50.0 - 2.0	50.0 - 2.8	50.0 - 1.7	50.0 - 1.6	50.0 - 2.0	50.0 - 2.0	50.0 - 1.6	50.0 - 2.4	50.0 - 1.8	50.0 - 1.7	50.0 - 2.1	50.0 - 1.8
R_{sym} (%) [a]	9.0 (29.5)	15.9 (48.0)	8.7 (47.5)	5.9 (32.8)	11.1 (40.8)	10.9 (45.1)	9.8 (81.5)	18.3 (78.3)	14.8 (92.4)	5.6 (21.4)	9.8 (49.8)	7.6 (40.9)
$I/\sigma I$ [a]	17.0 (6.6)	3.80 (3.43)	15.9 (4.5)	20.1 (5.4)	14.1 (5.6)	13.0 (4.0)	8.82 (1.72)	8.88 (3.15)	8.84 (1.68)	23.5 (8.7)	10.3 (2.6)	9.9 (2.1)
Completeness (%) [a]	99.9 (99.3)	98.0 (93.2)	98.4 (96.9)	97.8 (96.6)	98.8 (93.4)	99.9 (99.5)	99.4 (97.7)	99.9 (99.9)	99.7 (98.6)	98.5 (97.3)	99.5 (98.2)	94.0 (96.2)
Refinement												
R_{work} / R_{free} (%)[b]	15.9 / 20.1	18.2 / 24.8	14.8 / 18.8	17.3 / 20.0	15.1 / 19.8	16.4 / 20.0	18.6 / 21.4	16.2 / 22.5	18.4 / 22.9	14.4 / 17.5	15.4 / 21.1	18.6 / 21.9
Number of ligands molecules [c]	-<br>1 IMD<br>9 Zn^{2+}<br>325 $H_2$0	-<br>1 IMD<br>8 Ni^{2+}<br>124 $H_2$0	1 Met-Ala-Ser<br>1 ACT<br>8 Zn^{2+}<br>367 $H_2$0	1 Met-Ala-Arg<br>1 ACT<br>8 Ni^{2+}<br>351 $H_2$0	1 actinonin<br>1 ACT<br>8 Zn^{2+}<br>251 $H_2$0	1 AT002<br>1 ACT + 1 IMD<br>8 Zn^{2+}<br>246 $H_2$0	1 AT018<br>1 ACT + 1 IMD<br>9 Zn^{2+}<br>263 $H_2$0	1 AT019<br>1 ACT + 1 IMD<br>9 Zn^{2+}<br>193 $H_2$0	1 AT020<br>1 ACT + 1 IMD<br>9 Zn^{2+}<br>240 $H_2$0	1 AB47<br>1 ACT<br>8 Zn^{2+}<br>408 $H_2$0	1 SMP289<br>1 ACT + 1 IMD<br>9 Zn^{2+}<br>233 $H_2$0	1 RAS358<br>1 ACT + 1 IMD<br>9 Zn^{2+}<br>266 $H_2$0
r.ms.d of bond lengths (Å) /	0.014	0.030	0.033	0.009	0.031	0.014	0.012	0.021	0.015	0.034	0.024	0.015
r.ms.d of bond angles (°)	1.557	2.606	2.514	1.165	2.393	1.475	1.357	1.875	1.414	2.613	2.311	1.529
**PDB code**	**5JEX**	**5JEY**	**5JEZ**	**5JF0**	**5JF1**	**5JF2**	**5JF3**	**5JF4**	**5JF5**	**5JF6**	**5JF7**	**5JF8**

One single crystal was used for each data set.
[a] Values in parentheses are for highest-resolution shell.
[b] R_{free} is a test set including 5% of the data.
[c] IMD and ACT are for imidazole and acetate respectively.

Supplementary Table 3

PDF-In	MIC (µg/mL)		
	B. subtilis	***S. aureus***	***E. faecalis***
	**168**	**SA113**	**EFA 103.214**
AB47	8 - 4	32-16	32-16
SMP195	2 - 1	4	16
SMP289	16 - 8	4	16
AT003	16 - 8	>64	>64
AT004	64 - 32	>64	>64
AT007	>64	>64	>64
AT009	>64	>64	>64
AT010	>64	>64	>64
AT002	32 - 16	>64	>64
AT006	64 - 32	>64	>64
AT008	64 - 32	>64	>64
AT011		>64	>64
AT012		>64	>64
AT013		>64	>64
AT014		>64	>64
AT015		>64	>64
AT016		>64	>64
AT017		>64	>64

Chemistry

1. Synthesis of oxazoles and oxadiazoles

Syntheses began with the preparation of the common Evans chiral intermediates (2*R*)-(2-*tert*-butoxy-2-oxoethyl)hexanoic acid **3a** and (2*R*)-4-*tert*-butoxy-2-(cyclopentylmethyl)-4-oxobutanoic acid **3b** as shown in ***Scheme S1***. The hexanoyl chloride or 3-cyclopentylpropanoyl chloride was coupled in THF with the chiral auxiliary, 4(*S*)-benzyloxazolidinone, in the presence of BuLi. Alkylation of the *N*-acyl derivative **1a** or **1b** with *tert*-butyl 2-bromoacetate, followed by hydrolysis with LiOH-H_2O_2[1] provided the key acid intermediates **3a**[2] and **3b**[3] with good enantioselectivity (98%).

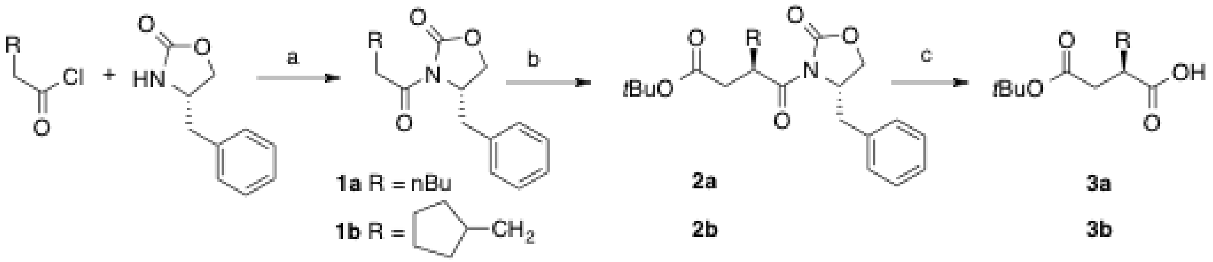

Scheme S1: Synthesis of succinic acid mono tert-butyl ester derivatives.
(a) BuLi, hexanoyl chloride or cylopentylpropanoyl chloride, THF (b) LiHMDS, $BrCH_2CO_2$*t*Bu, THF (c) 1) H_2O_2, LiOH, THF / H_2O) $Na_2S_2O_3$

In the next step, the acid **3a** was condensed wth *D*,*L*-2-phenylglycinol or ethyl-*L*-serinate, after activation with isobutylchloroformate (*i*-BuOCOCl) promoting the formation of the ß-hydroxy amide that was cyclised to 1,3-oxazole **4** and **5**, respectively, in CH_2Cl_2 with Deoxo-Fluor at -15°C and oxidized with bromotrichloromethane ($BrCCl_3$) and 1,8-diazabicyclo[5.4.0]undec-7-ene (DBU) as previously described.[4]

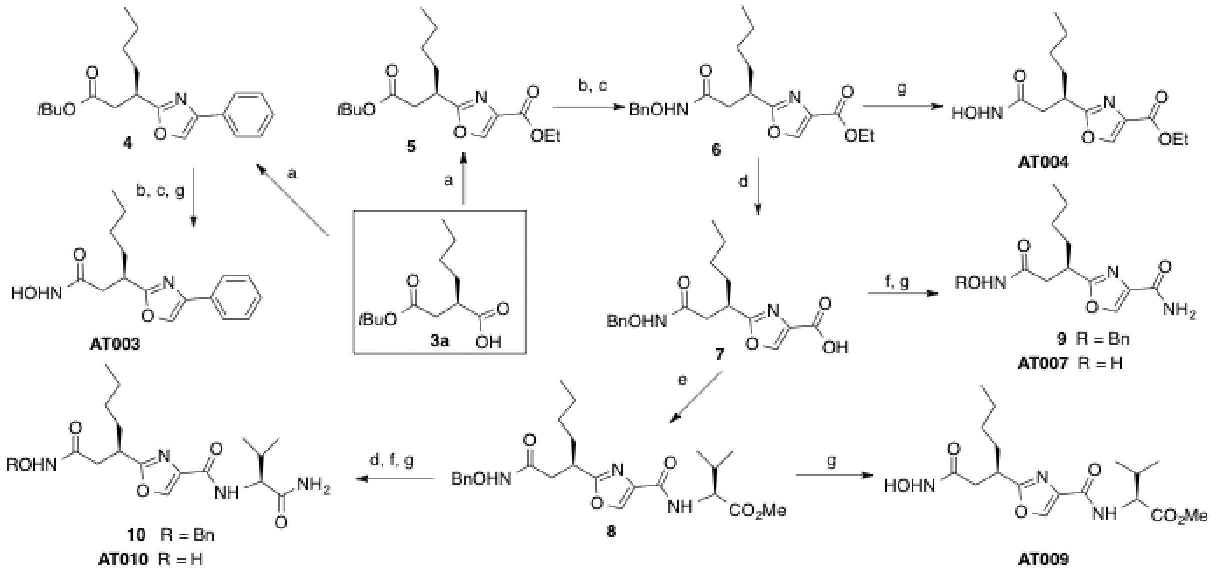

Scheme S2. Synthesis of hydroxamic acid oxazoles.
(a) 1) *i*-BuOCOCl, Et_3N, *D*,*L*-2-phenylglycinol or ethyl-*L*-serinate hydrochloride, THF 2) Deoxo-Fluor, $BrCCl_3$, DBU, CH_2Cl_2 (b) TFA, CH_2Cl_2 (c) NH_2OBn, CDI, Et_3N, THF (d) LiOH, 1,4-dioxane-H_2O (e) HOBt, EDCI, NMM, methyl-*L*-valinate hydrochloride, CH_2Cl_2 (f) *i*-BuOCOCl, Et_3N, NH_3, THF (g) H_2, Pd/$BaSO_4$, EtOH

Then, after acid hydrolysis of the *tert*-butyl ester, the protected OBn hydroxamic acid **6** was built by standard coupling of the acid with NH_2OBn using 1,1-carbonyldiimidazole (CDI) as activator. Saponification under mild conditions with LiOH in 1,4-dioxane / water mixture gave the acid **7**, from which were first prepared the amide **9** after activation of the acid with *i*-BuOCOCl and bubling with NH_3 gas, then the vanilyl adduct **8** by reaction with methyl-*L*-valinate under standard coupling conditions with *N*-hydroxybenzotriazole (HOBt) and 1-ethyl-3-(3-dimethylaminopropyl)carbodiimide (EDCI). Derivative **8** was further converted into the amide **10** after saponification of the methyl ester and amidification with NH_3 as described for **9**. Deprotection of the hydroxamic acids upon hydrogenolysis with H_2 over Pd/$BaSO_4$ in EtOH[5] provided the final hydroxamic oxazole derivatives, AT004, AT007, AT009, and AT010, shown in ***Scheme S2***. Final conversion of **4** into hydroxamic acid after acid hydroysis of the *t*-Bu ester gave AT003.

Hydroxamic oxadiazoles synthesis is outlined in ***Scheme S3***. The 3-aryl or 3-aryl-methyl-1,2,4-oxadiazolyl derivatives **12x** were prepared in two steps[6] from the amidoximes **11a-i** obtained by condensing the corresponding nitriles with hydroxylamines. Thermal dehydration in xylene

at reflux afforded 1,2,4-oxadiazoles.[7] This procedure was not efficient for the synthesis of **12k** bearing a benzothiazolemethyl substituent and another one-pot synthesis under milder conditions was preferred. Condensation of the amidoxime **11k** with **3a** and cyclisation into 1,2,4-oxadiazole were performed with propylphosphonic anhydride T3P® in AcOEt at room temperature[8] yielding **12k** in low yield. This procedure was applied to the syntheses of **12l**, **13i** and **13j**.

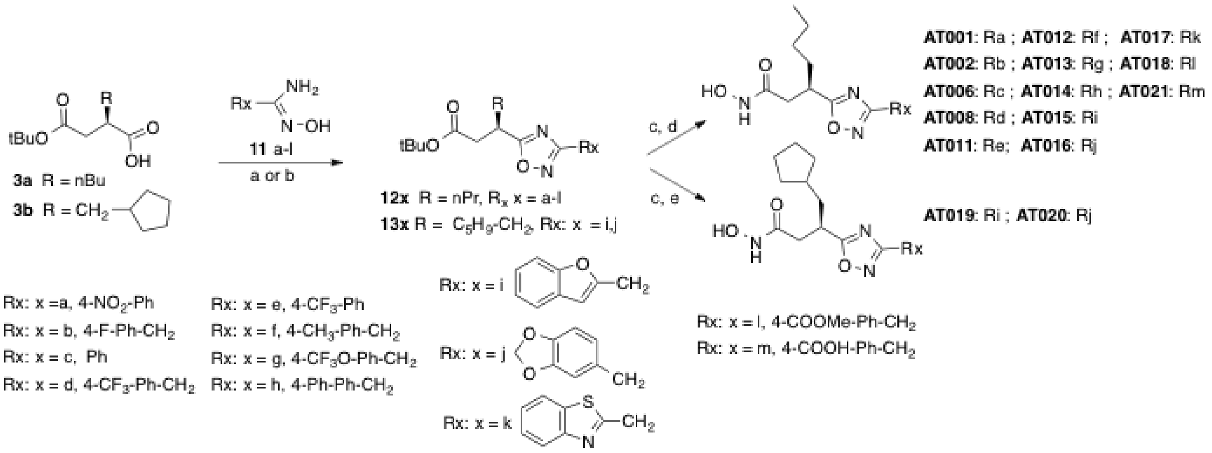

Scheme S3: Synthesis of hydroxamic acid oxadiazoles.
(a) 1) *i*-BuOCOCl, Et_3N, THF 2) xylene, reflux or (b) Et_3N, T3P®, AcOEt (c) TFA:CH_2Cl_2 1:1 (d) 1) *i*-BuOCOCl, Et_3N, THF 2) NH_2OSiMe_2*t*-Bu 3) *n*Bu_4NF (e) 1) NH_2OBn, CDI, Et_3N, THF 2) H_2, Pd/$BaSO_4$,EtOH

Another procedure was also used to prepare the hydroxamic acids.[9] After acid hydrolysis of the *tert*-butyl esters in **12** and **13** and activation of the acids with *i*-BuOCOCl to form mixed anhydrides, condensation with *O*-(*tert*-butyldimethylsilyl)hydroxylamine (NH_2OSiMe_2*t*-Bu) in THF, followed by deprotection with tetrabutylammonium fluoride (*n*-Bu_4NF) afforded cleanly the expected hydroxamic acids. Following this procedure, the ester AT018 was obtained in mixture with the acid AT021 due to some hydrolysis of the methyl ester. The two compounds were separated by chromatography.

2. Synthetic procedures

Materials

All solvents and chemicals were purchased from SDS and Aldrich, respectively. DMF, MeOH and CH_3CN were dried using standard. ^{1}H NMR and ^{13}C NMR spectra were recorded on Bruker ARX-250 and Bruker Avance-500 spectrometers, respectively, and chemical shifts were

reported in ppm downfield from TMS. IR spectra were obtained with a Perkin-Elmer Spectrum One FT-IR spectrometer equivuipped with a MIRacle™ single reflection horizontal ATR unit (germanium crystal). Electrospray ionization (ESI) mass spectrometry analyses were obtained using Thermo Finnigan LCQ Advantage spectrometer. HRMS and elemental analyses were carried out by the mass spectrometry and microanalysis services in Gif (CNRS).

Methods

Synthesis of acids 3

(2*R*)-2-(2-*tert*-butoxy-2-oxoethyl)hexanoic acid (3a) and **(2*R*)-4-*tert*-butoxy-2-(cyclopentylmethyl)-4-oxobutanoic acid (3b)** were synthesized following procedures previously described.[2, 3]

(S)-4-benzyl-3-hexanoyloxazolidin-2-one (1a) was obtained in quantitative yield (15.5g) from (*S*)-4-benzyl-2-oxazolidinone (56.4 mmol, 10 g) and hexanoyl chloride (75 mmol, 10.4 mL). No purification was required, and the isolated product was directly used in the next step. ^{1}H NMR (250 MHz, $CDCl_3$) δ (ppm) : 0.94 (t, *J* = 6.25 Hz, 3H); 1.39 (m, 4H); 1.71 (m, 2H); 2.79 (m, 1H); 2.97 (m, 2H); 3.27 (m, 1H); 4.19 (m, 2H); 4.67 (m, 1H); 7.28 (m, 5H). ^{13}C NMR (125.7 MHz, $CDCl_3$) δ (ppm) : 174.8, 154.8, 136.8, 130.9, 130.7, 130.4, 130.2, 128.8, 67.5, 56.5, 39.3, 36.9, 32.7, 25.4, 23.8, 15.3.

***tert*-butyl 3-(4-benzyl-2-oxooxazolidine-3-carbonyl)heptanoate (2a)** was prepared from **1a (**51.9 mmol, 14.29 g) and isolated as a white solid powder after chromatography (SiO_2, cyclohexane:AcOEt 90:10 to 85:15), 17.2 g (yield 85 %). ^{1}H NMR (250 MHz, $CDCl_3$) δ (ppm) : 0.91 (t, *J* = 6 Hz, 3H); 1.34 (m, 4H); 1.45 (s, 9H); 1.68 (m, 2H); 2.50 (m, 1H); 2.8 (m, 2H); 3.37 (m, 1H); 4.21 (m, 3H); 4.69 (m, 1H); 7.32 (m, 5H). ^{13}C NMR (125.7 MHz, $CDCl_3$) δ (ppm) : 177.4, 172.8, 154.4, 137.2, 130.9, 130.8, 130.3, 128.6, 82, 67.3, 57, 40.7, 39, 38.5, 33.12, 30.4, 29.5, 24, 15.3.

2-(2-*tert*-butoxy-2-oxoethyl)hexanoic acid (3a), was prepared from *N*-acyl-oxazolidinone (34.9 mmol, 13.6 g) upon treatment with H_2O_2 followed by aqueous LiOH and isolated as a

colorless oil which could be used without further purification, 6.84 g (yield 83%). ^{1}H NMR (250 MHz, $CDCl_3$) δ (ppm) : 0.93 (t, *J* = 6.5 Hz, 3H); 1.36 (m, 4H); 1.45 (s, 9H); 1.69 (m, 2H); 2.38 (m, 1H); 2.63 (m, 1H); 2.83 (m, 1H); 11.24 (s, 1H). ^{13}C NMR (250 MHz, acetone-D6) δ (ppm) : 14.6, 23.6, 28.6, 30.2, 32.6, 38.3, 42.2, 81, 172.1, 177.2.

(4*S*)-4-benzyl-3-(3-cyclopentylpropanoyl)-1,3-oxazolidin-2-one (1b) was prepared from (*S*)-4-benzyl-2-oxazolidinone (16.9 mmol, 3g) and cyclopentylpropionylchloride (22 mmol, 3.37 mL) and isolated after purification by chromatography (SiO_2, cyclohexane:AcOEt, 9:1), 4.18 g (yield 82%). ^{1}H NMR (250 MHz, $CDCl_3$) δ (ppm): 1.18 (m, 2H); 0.85-1.85 (m, 9H); 2.8 (dd, *J* = 9.6 and 13.3 Hz, 1H); 3.94 (m, 2H); 3.33 (dd, *J* = 3.4 and 13.3 Hz, 1H); 4.21 (m, 2H); 4.70 (m; 1H); 7.2-7.4 (m, 5H).

***tert*-butyl (3*R*)-4-[(4*S*)-4-benzyl-2-oxo-1,3-oxazolidin-3-yl]-3-(cyclopentylmethyl)-4-oxobutanoate (2b):** was isolated from **1b** (13.7mmol, 4.13g) after precipitation from CH_2Cl_2 solution into pentane, 4.38g (yield 77%). ^{1}H NMR (250 MHz, $CDCl_3$) δ (ppm): 1.19 (m, 2H); 1.46 (s, 9H); 1.47-1.9 (m, 9H); 2.55 (dd, *J* = 4.6 and 16.6 Hz, 1H); 2.78 (m, 2H); 3.38 (dd, *J* = 3.4 and 13.4 Hz, 1H); 4.19 (d, *J* = 4.6 Hz, 2H); 4.25 (m,1H); 4.68 (m,1H); 7.34 (m, 5H).

(2*R*)-4-*tert*-butoxy-2-(cyclopentylmethyl)-4-oxobutanoic acid (3b) was prepared from **2b** (7.2 mmol, 3 g): 1.78 g (yield 96%). ^{1}H NMR(500 MHz, $CDCl_3$) δ (ppm): 1.10 (m, 2H); 1.44 (s, 9H); 1.52 (m, 3H); 1.61 (m, 2H); 1.75(m, 2H); 1.85 (m, 2H); 2.4 (dd, *J* = 5.3 and 16.3 Hz, 1H); 2.62 (dd, *J* = 9.2 and 16.3 Hz, 1H); 2,8 (m, 1H); 11,6(s, 1H). 13C NMR (125.7 MHz, $CDCl_3$) δ (ppm): 26.4, 26.5, 29.4, 34, 34.1, 39.1, 39.2, 39.6, 42.4, 82.4, 172.5, 183.2

Synthesis of oxazoles

Ethyl 2-[1-(2-*tert*-butoxy-2-oxoethyl)pentyl]-1,3-oxazole-4-carboxylate (5) was synthesized in two steps as previously described[4] with some modifications: the first step led to the β-hydroxy amide followed in the second step by cyclization and oxidation to oxazole.

Preparation of β-hydroxy amide

To a solution of 2-(2-*tert*-butoxy-2-oxoethyl)hexanoic acid **(3a)** (2.39 mmol, 550 mg) in THF(10 mL) were added at 0°C under argon Et_3N (2.63 mmol, 370 µL) and isobutylchloroformate (2.5 mmol, 315 µL). The mixture was stirred for 1 h and then filtered. To a solution of ethyl-*L*-serinate hydrochloride (2.63 mmol, 450 mg) in THF (13 mL) was added Et_3N (2.63 mmol, 370 µL) then the mixture was stirred for 1 h at rt. This mixture was added to the previous filtrate and the reaction was allowed to warm to rt and stirred overnight. The solvent was evaporated to dryness and the residue was dissolved in CH_2Cl_2 and washed with saturated aqueous $NaHCO_3$, aqueous HCl 0.1N and brine. The organic layer was then dried over $MgSO_4$, filtered and concentrated. The residue was then purified by chromatography on silica gel (cyclohexane:AcOEt, 1:1) to give the β-hydroxyamide, *tert*-butyl 3-(1-ethoxy-3-hydroxy-1-oxopropan-2-ylcarbamoyl)heptanoate as a colorless oil in 66 % yield (550 mg).

^{1}H NMR (250 MHz, acetone-*d6*) δ (ppm): 0.91 (t, *J* =7 Hz, 3H); 1.26 (t, *J* = 7 Hz, 3H); 1.37 (m, 4H); 1.43 (9H, s); 1.64 (m, 2H); 2.31 (dd, *J* = 16.2 and 6 Hz, 1H); 2.57 (dd, *J* = 16.2 and 9 Hz, 1H); 2.79 (m, 2H); 3.86 (m, 2H); 4.17 (q, *J* = 7 Hz, 2H); 4.55 (m, 1H); 7.29 (d, *J* = 7.5 Hz, 1H). ^{13}C NMR (250 MHz, acetone-*d6*) δ (ppm): 175.6, 172.5, 171.6, 81.0, 63.5, 61.8, 56.1, 43.5, 39.1, 33.4, 30.3, 28.6, 23.7, 19.7, 14.7.

Cyclization and oxidation

Deoxo-Fluor (0.51 mmol, 191 µL) was added dropwise to a solution of *tert*-butyl 3-(1-ethoxy-3-hydroxy-1-oxopropan-2-yl-carbamoyl)heptanoate (0,47 mmol, 162 mg) in freshly distilled CH_2Cl_2 (5 mL) cooled to -15°C under argon. After 30 min, $BrCCl_3$ (1.55 mmol, 145 µL, 3.3 equiv) was added to the reaction mixture, followed by DBU (1.55 mmol, 230 µL; 3.3equiv). The reaction mixture was stirred for 7 h at 0°C, and overnight at rt. Then, $BrCCl_3$ (4,7 mmol, 440 µL, 10 equiv) was added, followed by DBU (4.7 mmol, 69 µL, 10 equiv). The mixture was heated to 50°C for 48 h, cooled at rt then quenched with saturated aqueous bicarbonate (5mL). The mixture was successively extracted with Et_2O and AcOEt and the combined organic layer

was washed with HCl 0,1 N, brine then dried over $MgSO_4$, filtered and concentrated. Purification of the slurry by chromatography (SiO_2, cyclohexane:AcOEt 5:1) gave oxazole **(5)** as a yellow oil, in 42 % yield (65 mg).

IR (neat, ν cm^{-1}) : 2971, 2934, 2868, 1728, 1582. ^{1}H NMR (250 MHz, $CDCl_3$) δ (ppm) : 0.84 (t, *J* = 7.5 Hz, 3H); 1.23 (m, 4H); 1.35 (t, *J* = 7.5 Hz, 3H); 1.37 (s, 9H); 1.70 (m, 2H); 2.56 (dd, *J* = 15.7 and 6.2 Hz, 1H); 2.79 (dd, *J* = 15.7 and 8.5 Hz, 1H); 3.36 (m, 1H); 4.35 (q, *J* = 7.5 Hz, 2H); 8.12 (s, 1H). ^{13}C NMR (250 MHz, acetone-*d6*) δ (ppm): 170.54, 167.68, 161.23, 144.51, 133.72, 80.34, 60.66, 38.87, 36.16, 33.24, 29.25, 27.67, 22.60, 14.1, 13.67.

Ethyl 2-(1-(benzyloxyamino)-1-oxoheptan-3-yl)oxazole-4-carboxylate (6) was obtained by following a procedure previously described for similar products[5] with some modifications.

2-[1-(2-*tert*-butoxy-2-oxoethyl)pentyl]-1,3-oxazole-4-carboxylic ethyl ester **(5)** 347 mg (1.06 mmol) was dissolved in 10 mL CH_2Cl_2 and 10 mL TFA at 0°C. After stirring for 12 h the solution was concentrated to give 3-(4-(ethoxycarbonyl)oxazol-2-yl)heptanoic acid in quantitative yield (288 mg) which was used without further purification. It was diluted in dry THF (10mL) then CDI (172 mg, 1.06 mmol) was added. After stirring for 1 h, it was added NH_2OBn, prealably dechlorhydrated in THF (5 mL) with Et_3N (181 µl, 1.06 mmol). After 24 h stirring, the yellow mixture was concentrated, diluted in AcOEt (20 mL), successively washed with saturated aqueous $NaHCO_3$ (20 mL), water (20mL), HCl 1N (20 mL) and brine (20 mL) before being dried over Na_2SO_4, then filtered and concentrated to give **6** as a yellow oil in 80 % yield (320 mg). ^{1}H NMR (250 MHz, acetone-*d6*) δ (ppm): 0.88 (t, *J* = 7.5 Hz, 3H); 1.29 (m, 4H); 1.32 (t, *J* = 7 Hz, 3H); 1.74 (m, 2H); 2.55 (m, 2H); 3.45 (m, 1H); 4.31 (q, *J* = 7 Hz, 2H); 4.85 (s, 2H); 7.39 (m, 5H); 8.47 (s, 1H); 10.2 (s, 1H).

2-(1-(benzyloxyamino)-1-oxoheptan-3-yl)oxazole-4-carboxylic acid (7): the ethyl ester **6** (320 mg) was saponified under mild conditions at 0°C with LiOH (4 equiv, 0.1 M) in dioxane:water (1:1). After 1 h, water was added. The aqueous phase was washed with diethyl

ether then acidified to pH 4 with HCl 1N and extracted with AcOEt before being dried over Na_2SO_4, filtered and concentrated to give a white solid in quantitative yield (296 mg). IR (neat, ν cm^{-1}): 3262, 2953, 2932, 1715, 1654, 1581. ^1H NMR (250 MHz, acetone--*d6*) δ (ppm): 0.88 (t, *J* = 7 Hz, 3H); 1.29 (m, 4H); 1.75 (q, *J* = 7Hz, 2H); 2.55 (m, 2H); 3.47 (m, 1H); 4.85 (s, 2H); 7.38 (m, 5H); 8.47 (s, 1H); 10.2 (s, 1H).

Methyl 2-(2-(1-(benzyloxyamino)-1-oxoheptan-3-yl)oxazole-4-carboxamido)-3-methyl butanoate (8) To 2-(1-(benzyloxyamino)-1-oxoheptan-3-yl)oxazole-4-carboxylic acid **(7)** (155 mg, 0.45 mmol) dissolved in CH_2Cl_2 (15 mL) were successively added HOBt (60 mg, 0.45 mmol) and methyl-*L*-valinate hydrochloride (75 mg, 0.45 mmol) previously neutralized in 5 mL CH_2Cl_2 with NMM (98 µL, 0.45 mmol), then finally EDCI (86 mg, 0.45 mmol). After stirring for 15 h, 15 mL CH_2Cl_2 were added and the mixture was successively washed with saturated aqueous $NaHCO_3$, water, HCl 1N and brine before being dried over Na_2SO_4, filtered and concentrated to give a colourless oil which could be used without further purification. Yield: 95 % (200 mg). ^1H NMR (500MHz, $CDCl_3$) δ (ppm): 0.89 (t, *J* = 7,5 Hz, 3H); 0.99 (d, *J* = 7 Hz, 3H); 1.01 (d, *J* = 7 Hz, 3H); 1.29 (m, 4H); 1.74 (m, 2H); 2.26 (dh, *J* = 5.2 and 7 Hz, 1H); 2.52 (m, 2H); 3.49 (q, *J* = 7 Hz, 1H); 3.77 (s, 3H); 4.69 (dd, *J* = 5.3 Hz, 1H); 4.89 (s, 2H); 7.37 (m, 5H); 8.08 (s, 1H); 8.23 (s, 2H)

2.2 General procedure for amide synthesis: *iso*-Butylchloroformate (51 µl, 0.39 mmol) was added to a stirred solution of acid (0.35 mmol) and *N*-methylmorpholine (43 µl, 0.39 mmol) in THF (10 mL) at 0°C. The mixture was stirred for 1 h, then ammonia gas was *bubbled* for 30 min. After 1h of additional stirring, the mixture was concentrated, diluted in AcOEt, washed with brine, dried (Na_2SO_4) and concentrated. The slurry was triturated in diethyl ether and filtered to provide a white solid in quantitative yield.

2-(1-(benzyloxyamino)-1-oxoheptan-3-yl)oxazole-4-carboxamide (9) (m 120 mg) IR (neat, ν cm^{-1}): 3210, 2958, 2932, 1692, 1660, 1610, 1110. ^1H NMR (500 MHz, acetone-*d6*) δ (ppm):

0.88 (t, J = 7.2 Hz, 3H); 1.29 (m, 4H); 1.74 (m, 2H); 2.54 (m, 2H); 3.44 (m, 1H); 4.84 (s, 2H); 6.66 (s, 1H); 7.11 (s, 1H); 7.37 (m, 5H); 8.25 (s, 1H); 10.16 (s, 1H). ^{13}C NMR (125.7 MHz, acetone-D6) δ (ppm):14.7, 23.6, 30.3, 34.2, 37.1, 37.4, 78.8, 129.7, 130.3, 137.6, 142.8, 163.9, 167.9, 169.2.

N-(1-amino-3-methyl-1-oxobutan-2-yl)-2-(1-(benzyloxyamino)-1-oxoheptan-3-yl)oxazole-4-carboxamide (10) (m 155 mg) IR (neat, ν cm^{-1}) : 3192, 29662, 2933, 1730, 1649, 1600, 1515, 1109. ^{1}H NMR (500 MHz, acetone-*d6*) δ (ppm): 0.88(t, J = 7.1Hz, 3H); 0.95(d, J = 7Hz, 3H); 0.99(d, J = 7Hz, 3H); 1.3 (m, 4H); 1.75 (m, 2H); 2.18 (m, 1H); 2.55 (m, 2H); 3.46 (m, 1H); 4.5 (m, 1H); 4.84 (s, 2H); 6.56 (s, 1H); 7.12 (s, 1H); 7.38 (m, 5H); 7.51 (d, J = 8.2Hz, 1H); 8.27 (s, 1H); 10.21 (s, 1H).

***tert*-butyl (*R*)-3-(4-phenyloxazol-2-yl)heptanoate 4** obtained from **3a** (1.2 mmol 276 mg) in 40 % yield (m 158 mg) IR (neat, ν cm^{-1}) : 1732, 1682. ^{1}H NMR (250 MHz,$CDCl_3$) δ (ppm): 0.83 (t, J = 6.5 Hz, 3H); 1.23 (m, 4H); 1.31 (s, 9H); 1.69 (m, 2H); 2.53 (dd, J = 15.5 and 6.5, 1H); 2.75 (dd, J = 15.5 and 6.5 Hz, 1H); 3.32 (m, 1H); 7.23 (t, J = 7, 1H); 7.32 (d, t = 7, 2H); 7.66 (d, J = 7, 2H); 7.75 (s, 1H). ESI$^+$ MS *m/z*: [M + H]$^+$ 330.2, [M + Na]$^+$ 352.1.

General procedure for hydroxamic acid synthesis via hydrogenation: hydrogenolysis was performed on 5% Pd/$BaSO_4$ in EtOH as previously reported[5] 5% Pd / $BaSO_4$ (0.1equiv) was added to the benzyloxyamino product (1equiv) in EtOH. The mixture was stirred for 4 h under H_2 atmosphere, then filtered over celite, concentrated and precipitated from CH_2Cl_2 into Et_2O (CH_2Cl_2:Et_2O 1:10) to give a white powder in quantitative yield.

(*R*)-*N*-hydroxy-3-(4-phenyloxazol-2-yl)heptanamide (AT003) : 138 mg obtained from **4** (0.48 mmol 158 mg). ^{1}H NMR (500 MHz, acetone-*d6*) δ (ppm): 0.88 (t, J = 6.5, 3H); 1.31 (m, 4H); 1.77 (m, 2H); 2.53 (dd, J = 14.5 and 7 Hz, 1H); 2.70 (dd, J = 14.5 and 7 Hz, 1H); 3.45 (m, 1H); 7.38 (m, 3H); 7.82 (d, J = 7.5, 2H); 8.28 (s, 1H); 10.07 (s, 1H). El. Anal.: calcd for $C_{16}H_{20}N_2O_3$•0.25Et_2O : C 66.31; H 8.17; N 7.97; found C 66.49: H8.01; N 7.96.

Ethyl 2-{1-[2-(hydroxyamino)-2-oxoethyl]pentyl}-1,3-oxazole-4-carboxylate (AT004) : 75 mg obtained from **6** (100 mg, 0.27 mmol). IR (neat, ν cm^{-1}): 3216, 2958, 2932, 2872, 1726, 1658, 1581, 1109. ^{1}H NMR (500 MHz, acetone-*d6*) δ (ppm): 0.87 (t, *J* = 7.2 Hz, 3H); 1.28 (m, 4H); 1.33 (t, *J* = 7 Hz, 3H); 1.74 (m, 2H); 2.51 (dd, *J* = 7 and 14.5 Hz, 1H); 2.64 (dd, *J* = 7 and 14.5 Hz, 1H); 3.44 (q, *J* = 7.1 Hz, 1H); 4.31 (q, *J* = 7.1 Hz, 2H); 8.02 (s, 1H), 8.44 (s, 1H); 10.02 (s, 1H). ^{13}C NMR (125.7 MHz, acetone-*d6*), δ (ppm): 14.6, 15.1, 30.6, 30.7, 34.2, 37, 61.7, 134.7, 145.6, 162.2, 168.8. ESI^{+} MS, *m/z*: [M+H]$^{+}$ 284.9. El. anal.: calcd for $C_{13}H_{20}N_2O_5$: C, 54.91; H, 7.09; N, 9.85; found: C, 55.18; H, 7.11; N, 9.25.

2-{1-[2-(hydroxyamino)-2-oxoethyl]pentyl}-1,3-oxazole-4-carboxamide (AT007) : 74.4 mg from **9** (100 mg, 0.29 mmol). IR (neat, ν cm^{-1}): 3412, 3200, 3111, 2958, 2929, 2863, 1646, 1609, 1124, 1112. ^{1}H NMR (500 MHz, acetone-*d6*) δ (ppm): 0.87(t, *J* = 7.1 Hz, 3H); 1.29 (m, 4H); 1.75 (m, 2H); 2.50 (dd, *J* = 6.2 and 14.6 Hz, 1H); 2.64 (dd, *J* = 6.2 and 14.6 Hz, 1H); 3.44 (q, *J* = 7 Hz, 1H); 6.78 (s, 1H), 7.18 (s, 1H); 8.26 (s, 1H). ^{13}C NMR (125.7 MHz, acetone-*d6*) δ (ppm): 14.6, 23.6, 34.2, 37.1, 137.7, 142.5, 163.5, 167.8, 169. ESI^{+} HRMS calcd for [M+Na]$^{+}$ $C_{11}H_{17}N_3O_4Na$ 278.1117, found 278.1118. El. Anal.: calcd for $C_{11}H_{17}N_2O_4$: C, 51.76; H, 6.71; N, 16.46; found: C, 51.71; H, 6.78; N, 15.75.

Methyl *N*-[(2-{1-[2-(hydroxyamino)-2-oxoethyl]pentyl}-1,3-oxazol-4-yl)carbonyl]valinate (AT009) : m 80 mg from **8** (100 mg, 0.217 mmol). IR (neat, ν cm^{-1}): 3245, 2963, 2933, 2875, 1784, 1742, 1653, 1204, 1166. ^{1}H NMR (500 MHz, acetone-*d6*) δ (ppm): 0.86 (m, 3H); 0.97 (d, *J* = 6 Hz, 6H); 1.28 (m, 4H); 1.74 (m, 2H); 2.23 (m, 1H); 2.52 (m, 1H); 2.63 (m, 1H); 3.43 (m, 1H); 3.74 (s, 3H); 4.54 (m, 1H); 7.34 (s, 1H); 7.38 (s, 1H); 7.49 (d, *J* = 6 Hz, 1H); 8.31 (s, 1H). ^{13}C NMR (125.7 MHz, acetone-*d6*) δ (ppm): 14.7, 18.9, 19.9, 23.6, 30.6, 30.8, 32.4, 34, 37.1, 52.9, 58.3, 137.1, 142.6, 161.5, 168.2, 169.1, 173.1. ESI^{+} HRMS: calcd for [M+Na]$^{+}$ $C_{17}H_{27}N_3O_6Na$ 392.1798; found 392.1801.

***N*²-[(2-{1-[2-(hydroxyamino)-2-oxoethyl]pentyl}-1,3-oxazol-4-yl)carbonyl]valinamide (AT010):** 79.7 mg from **10** (100 mg 0.22 mmol). IR (neat, ν cm^{-1}): 3198, 2961, 2932, 2873, 1655, 1599, 1513, 1108. ^{1}H NMR (500 MHz, acetone-*d6*) δ (ppm): 0.87 (m, 3H); 0.99 (m, 6H); 1.28 (m, 4H); 1.75 (m, 2H); 2.19 (h, *J* = 7.1 Hz, 1H); 2.52 (m, 1H); 2.66 (m, 1H); 3.44 (m, 1H); 4.52 (m, 1H); 6.73 (d, *J* = 28.7 Hz, 1H); 7.29 (d, *J* = 28.7 Hz, 1H); 7.37 (m, 1H); 7.61 (m, 1H); 8.30 (s, 1H). ^{13}C NMR (125.7 MHz, acetone-*d6*) δ (ppm): 14.7, 18.8, 20.3, 23.6, 30.3, 32.9, 33, 34.1, 37.1, 137.5, 142.3, 161.4, 168.2, 169, 174.3. ESI$^+$ HRMS: calcd for [M+Na]$^+$ $C_{16}H_{26}N_4O_5Na$ 377.1801; found 377.1808.

Oxadiazole synthesis

General procedure for synthesis of amidoximes

Syntheses of **11a**,[10] **11b**,[7] **11c**,[10] **11d**,[7] **11f**,[7] and **11l**[11] were previously reported. The other amidoximes were prepared following the procedure described.10

(1*Z*)-*N*'-hydroxy-2-[4-(trifluoromethoxy)phenyl]ethanimidamide (11g) : product isolated 238 mg (41% yield) from the nitrile (2.48 mmol, 500 mg) after chromatography (SiO_2, cyclohexane:AcOEt, 1:1). ^{1}H NMR (250 MHz, DMSO-*d6*) δ (ppm): 3.28 (s, 2H); 5.46 (s, 2H); 7.34 (m, 4H); 8.93 (s, 1H). ^{13}C NMR (125.7 MHz, DMSO-*d6*) δ (ppm): 36.4, 120.1 (q, *J* = 263,9 Hz), 120.7, 130.4, 137.6, 146.8, 151.6. ESI$^+$ MS (MeOH) *m/z*: 235.1 [M+H]$^+$

(1*Z*)-2-biphenyl-4-yl-*N*'-hydroxyethanimidamide (11h) : product isolated 370.6 mg (63% yield) from the nitrile (2.6 mmol, 500 mg) after chromatography (SiO_2, cyclohexane:AcOEt, 1:1). ^{1}H NMR (250 MHz, DMSO-*d6*): 3,32 (m, 2H); 5,42(s, 2H); 7,52(m, 9H); 8,90 (s, 1H). ^{13}C NMR (125.7 MHz, DMSO-D6): 36.8; 126.4; 126.5; 127.2; 128.9; 129.2; 137.3; 138.2; 140.1; 151.9. ESI$^+$ MS (MeOH) *m/z*: 227.1 [M+H]$^+$.

(1*Z*)-2-(1-benzofuran-2-yl)-*N*'-hydroxyethanimidamide (11i) : product isolated 332.6 mg (55% yield) from the nitrile (3.18 mmol, 500 mg) after chromatography (SiO_2, cyclohexane:AcOEt, 1:1).^{1}H NMR (250 MHz, DMSO-*d6*) δ (ppm): 3.37 (s, 2H); 5.44 (s, 2H);

7.55 (m, 5H); 8.95 (s, 1H). ^{13}C NMR (125.7 MHz, DMSO-*d6*) δ (ppm): 25.6, 111.1, 116.1, 120.2, 122.3, 124.1, 127.7, 142.9, 150.7, 154.5. ESI^{+} MS (MeOH) *m/z*: 191.1[M+H]$^{+}$.

(1*Z*)-2-(1,3-benzodioxol-5-yl)-*N*'-hydroxyethanimidamide (11j) : product isolated 1.379 g (71 % yield) from the nitrile (10 mmol, 1.611 g) after chromatography (SiO_2, cyclohexane:AcOEt, 1:1). ^{1}H NMR (250 MHz, DMSO-*d6*) δ (ppm): 3.15 (s, 2H); 5.35 (s, 2H); 5.95 (s, 2H); 6.74 (m, 3H); 8.89 (s, 1H). ^{13}C NMR (125.7 MHz, DMSO-*d6*) δ (ppm): 36.8, 100.7, 107.9, 109.1, 121.6, 131.7, 145.6, 147.0, 152.1. ESI^{+} MS (MeOH) *m/z*: 195 [M+H]$^{+}$.

(1*Z*)-2-(1,3-benzothiazol-2-yl)-*N*'-hydroxyethanimidamide (11k) : product isolated 210.4 mg (35 % yield) from the nitrile (2.9 mmol, 500 mg) after chromatography (SiO_2, cyclohexane:AcOEt, 1:1).^{1}H NMR (250 MHz, DMSO-*d6*) δ (ppm): 3.82 (s, 2H); 5.69 (s, 2H); 7.44 (m, 2H); 7.99 (m, 2H); 9.18 (s, 1H). ^{13}C NMR (125.7 MHz, DMSO-*d6*) δ (ppm): 36.1, 121.9, 122.1, 124.8, 125.9, 135.2, 149.5, 152.5, 168.0. ESI^{+} MS (MeOH): 208 [M+H]$^{+}$

General procedure for 1,2,4-oxadiazoles (12a-k) synthesis

Two-step procedure: 1,2,4-oxadiazoles were prepared as described[6] with some modifications.

1a: *Preparation of N-acyloxy amidines*

To a dry THF solution of succinic acid mono *tert*-butyl ester derivative **3a** or **3b** (1 equiv) cooled to 0°C under argon, were added Et_3N (1.1 equiv) and *iso*-butylchloroformate (3.61 mmol, 1.05 equiv). After stirring for 30 min the solution was filtered and added to a THF solution of amidoxime (0.87 equiv). The mixture was stirred overnight under reflux. After removing the solvent in vacuo, the residue was dissolved in CH_2Cl_2 and washed with water and brine. The organic layer was then dried ($MgSO_4$), filtered and evaporated yielding the desired product as a yellow oil that was used in the next step without further purification.

1b: *cyclisation*

A xylene solution of *N*-acyloxy amidine was heated to reflux under argon overnight. The reaction mixture was cooled to rt, then the solvent was codistilled in vacuo with toluene. After

classical workup, the residue dissolved in CH_2Cl_2 was first filtered on silica gel, then purifed by chromatography on silica gel (cyclohexane:AcOEt) to yield oxadiazole as a yellow oil.

B-2: *One-step procedure*: Oxadiazoles were prepared as previously described.[8]

To an AcOEt solution of carboxylic acid **3a** or **3b** (1equiv) and amidoxime **11x** (1equiv) were added Et_3N (3 equiv), then dropwise T3P® (2.5 equiv) at rt. After 15 min, the mixture was heated to 80°C for 4 to 15 h (TLC control). The mixture was cooled to rt, poured onto ice-water and extracted with AcOEt. The combined organic phase was washed with saturated aqueous $NaHCO_3$ and brine before being dried over $MgSO_4$ and concentrated. Crude product (black oil) was purified by chromatography on silica gel (CH_2Cl_2) to provide oxadiazole as a yellow oil.

***tert*-butyl 3-[3-(4-nitrophenyl)-1,2,4-oxadiazol-5-yl]heptanoate (12a)**

Procedure B1 product isolated (766 mg, 65% yield) from **3a** (3.12 mmol) after chromatography (SiO_2 cyclohexane:AcOEt : 80:20). ^{1}H NMR (250 MHz, $CDCl_3$) δ (ppm): 0.92 (t, *J* = 6.7 Hz, 3H); 1.33 (m, 4H); 1.41 (s, 9H); 1.83 (m, 2H); 2.75 (dd, *J* = 16.5 and 5.7 Hz, 1H); 2.96 (dd, *J* = 16.5 and 8.7 Hz, 1H); 3.57 (m, 1H); 8.30 (d, *J* = 9 Hz, 2H); 8.36 (d, *J* = 9 Hz, 2H).

***tert*-butyl 3-[3-(4-fluorobenzyl)-1,2,4-oxadiazol-5-yl]heptanoate (12b)**

Procedure B1 product isolated (573 mg, 46% yield) from **3a** (3.44 mmol) after chromatography (SiO_2 cyclohexane:AcOEt, 80:20). ^{1}H NMR (250 MHz, $CDCl_3$) δ (ppm) : 0.83 (t, *J* = 6.8 Hz, 3H); 1.23 (m, 4H); 1.29 (s, 9H); 1.68 (m, 2H); 2.57 (m, 1H); 2.75 (m, 1H); 3.39 (m, 1H); 3.98 (s, 2H); 6.95 (m, 2H); 7.25 (m, 2H). ^{13}C NMR (125.7 MHz, $CDCl_3$) δ (ppm): 13.7, 22.3, 27.8, 28.9, 31.4, 32.8, 34.7, 38.5, 81, 115.4 (d, *J* = 21.7 Hz), 130.5 (d, *J* = 7.7 Hz), 131.37, 161.9 (d, ^1J = 245 Hz), 169, 170, 182.1. ESI$^+$ MS (/MeOH) *m/z*: 362.9 $[M+H]^+$; 385.1 $[M+Na]^+$

***tert*-butyl 3-(3-phenyl-1,2,4-oxadiazol-5-yl)heptanoate (12c)**

Procedure B1: product isolated (450 mg, 60% yield) from **3a** (2.17 mmol) after chromatography on silica gel (cyclohexane:AcOEt, 80:20), yield 47%.^{1}H NMR (250 MHz, acetone-*d6*) δ

(ppm) : 0.91 (t, *J* = 7Hz, 3H); 1.33 (m, 4H); 1.40 (s, 9H); 1.86 (m, 2H); 2.79 (dd, *J* = 16.2 and 6 Hz, 1H); 2.95 (dd, *J* = 16.2 and 6 Hz, 1H); 3.56 (m, 1H); 7.56 (m, 3H); 8.11 (m, 2H).

***tert*-butyl 3-{3-[4-(trifluoromethyl)benzyl]-1,2,4-oxadiazol-5-yl}heptanoate (12d)**

Procedure B1 – product isolated (110mg, 39% yield) from **3a** (0.69 mmol) after chromatography on silica gel (cyclohexane:AcOEt, 90:10). ^{1}H NMR (250 MHz, $CDCl_3$) δ (ppm) : 0.89 (t, *J* = 6.9 Hz, 3H); 1.32 (m, 13H); 1.74 (m, 2H); 2.71 (m, 2H); 3.45 (m, 1H); 4.14 (s, 2H); 7.45 (d, *J* = 8 Hz, 2H); 7.59 (d, *J* = 8 Hz, 2H). ^{13}C NMR (125.7 MHz, $CDCl_3$) δ (ppm): 15.2, 23.7, 29.2, 30.3, 33.5, 34.3, 36.1, 39.9, 82.5, 127 (q, *J* = 3.7 Hz); 130.7, 141, 169.9, 171.4, 183.8. ESI$^+$ MS (MeOH) *m/z*: 412.7 [M+H]$^+$; 435 [M+Na]$^+$

***tert*-butyl 3-{3-[4-(trifluoromethyl)phenyl]-1,2,4-oxadiazol-5-yl}heptanoate (12e)**

Procedure B1 product isolated (245 mg, 22% yield) from **3a** (2.8 mmol) after chromatography on silica gel (cyclohexane:AcOEt, 80:20). ^{1}H NMR (250 MHz, $CDCl_3$) δ (ppm): 0.86 (t, *J* = 6.7 Hz, 3H); 1.29 (m, 4H); 1.36 (s, 9H); 1.78 (m, 2H); 2.67 (m, 1H); 2.87 (m, 1H); 3.52 (m, 1H); 7.15 (m, 2H); 8.18 (m, 2H). ^{13}C NMR (125.7 MHz, $CDCl_3$) δ (ppm): 14.0, 22.6, 28.1, 29.2, 33.1, 35.0, 38.8, 81.4, 123.8 (q, *J* = 272 Hz), 126.0, 128.0, 130.6, 133.0 (q, *J* = 32.8 Hz), 167.4, 170.3, 182.7. ESI$^+$ MS (MeOH): 398.9 [M+H]$^+$

***tert*-butyl 3-[3-(4-methylbenzyl)-1,2,4-oxadiazol-5-yl]heptanoate (12f)**

Procedure B1: product isolated (657 mg, 47% yield) from **3a** (3.9 mmol) after chromatography (SiO_2, cyclohexane:AcOEt, 80:20). ^{1}H NMR (250 MHz, $CDCl_3$) δ (ppm): 0.83 (t, *J* = 6.9 Hz, 3H); 1.23 (m, 4H); 1.30 (s, 9H); 1.69 (m, 2H); 2.29 (s, 3H); 2.58 (m, 1H); 2.75 (m, 1H); 3.40 (m, 1H); 3.99 (s, 2H); 7.13 (m, 4H). ^{13}C NMR (125.7 MHz, $CDCl_3$) δ (ppm): 14.0, 21.2, 22.5, 28.1, 29.1, 32.1, 33.0, 34.8, 38.7, 81.2, 129.1, 129.5, 132.7, 136.7, 169.5, 170.3, 182.1. ESI$^+$ MS (MeOH) *m/z* : 358.9 [M+H]$^+$

***tert*-butyl 3-{3-[4-(trifluoromethoxy)benzyl]-1,2,4-oxadiazol-5-yl}heptanoate (12g)**

Procedure B1: product isolated (513 mg, 47% yield) from **3a** (2.55 mmol) after chromatography (SiO_2, cyclohexane:AcOEt, 80:20). ^{1}H NMR (250 MHz, $CDCl_3$) δ (ppm): 0.83 (t, *J* = 6.8 Hz, 3H); 1.23 (m, 4H); 1.27 (s, 9H); 1.69 (m, 2H); 2.58 (m, 1H); 2.75 (m, 1H); 3.39 (m, 1H); 4.27 (s, 2H); 7.22 (m, 4H). ^{13}C NMR (125.7 MHz, $CDCl_3$) δ (ppm): 14.0, 22.5, 28.0, 29.1, 31.8, 33.0, 34.9, 38.7, 81.3, 120.7 (q, *J* = 257 Hz), 121.3, 130.5, 134.5, 148.5, 168.9, 170.2, 182.5. ESI$^+$ MS (MeOH) *m/z*: 428.9 [M+H]$^+$

***tert*-butyl 3-[3-(biphenyl-4-ylmethyl)-1,2,4-oxadiazol-5-yl]heptanoate (12h)**

Procedure B1: product isolated (502 mg, 47% yield) from **3a** (2.54 mmol) after chromatography (SiO_2, cyclohexane:AcOEt, 80:20). ^{1}H NMR (250 MHz, $CDCl_3$) δ (ppm): 0.84 (t, *J* = 6.9 Hz, 3H); 1.23 (m, 4H); 1.29 (s, 9H); 1.7 (m, 2H); 2.59 (m, 1H); 2.77 (m, 1H); 3.41 (m, 1H); 4.07 (s, 2H); 7.4 (m, 9H). ^{13}C NMR (125.7 MHz, $CDCl_3$) δ (ppm) : 14.0, 22.5, 28.1, 29.2, 32.2, 33.1, 35.0, 38.8, 81.3, 127.3, 127.4, 127.6, 129.0, 129.6, 134.9, 140.2, 141.0, 169.4, 170.3, 182.3. ESI$^+$ MS (MeOH) *m/z*: 420.8 [M+H]$^+$.

***tert*-butyl 3-[3-(1-benzofuran-2-ylmethyl)-1,2,4-oxadiazol-5-yl]heptanoate (12i)**

Procedure B1: product isolated (707 mg, 47% yield) from **3a** (3.9 mmol) after chromatography (SiO_2, cyclohexane:AcOEt, 80:20). ^{1}H NMR (250 MHz, $CDCl_3$) δ (ppm): 0.82 (t, *J* = 6.7 Hz, 3H); 1.24 (m, 13H); 1.69 (m, 2H); 2.76 (m, 1H); 2.59 (m, 1H); 3.41 (m, 2H); 4.10 (s, 2H); 7.42 (m, 5H). ^{13}C NMR (125.7 MHz, $CDCl_3$) δ (ppm): 14.0, 21.4, 22.5, 28.1, 29.1, 33.1, 34.9, 38.8, 81.3, 111.7, 114.8, 120.0, 122.8, 124.7, 127.7, 142.9, 155.5, 168.4, 170.3, 182.4. ESI$^+$ MS (MeOH) *m/z*: 384.8 [M+H]$^+$.

***tert*-butyl 3-[3-(1,3-benzodioxol-5-ylmethyl)-1,2,4-oxadiazol-5-yl]heptanoate (12j)**

Procedure B1: product isolated (174.5 mg, 49% yield) from **3a** (1.025 mmol) after chromatography (SiO_2, cyclohexane:AcOEt, 80:20). ^{1}H NMR (250 MHz, $CDCl_3$) δ (ppm): 0.84 (t, *J* = 7 Hz, 3H), 1.23 (m, 4H), 1.31 (s, 9H), 1.69 (m, 2H), 2.58 (m, 1H), 2.75 (m, 1H), 3.4 (m, 1H), 3.94 (s, 2H), 5.90 (s, 2H), 6.74 (m, 3H). ^{13}C NMR (125.7 MHz, $CDCl_3$) δ (ppm): 14.0,

22.5, 28.0, 29.1, 32.1, 33.0, 34.9, 38.7, 81.3, 101.2, 108.5, 109.6, 122.2, 129.4, 146.8, 148.0, 169.5, 170.3, 182.2. ESI$^+$ MS (MeOH) *m/z*: 388.8 $[M+H]^+$.

***tert*-butyl 3-[3-(1,3-benzothiazol-2-ylmethyl)-1,2,4-oxadiazol-5-yl]heptanoate (12k)**

Procedure B1: product isolated (203 mg, 13% yield) from **3a** (3.9 mmol) after chromatography (SiO_2, cyclohexane:AcOEt, 80:20). ^{1}H NMR (250 MHz, $CDCl_3$) δ (ppm) : 0.86 (t, *J* = 6.81 Hz, 3H), 1.31 (m, 4H), 1.4 (s, 9H), 1.71 (m, 2H), 2.60 (m, 1H), 2.79 (m, 1H), 3.45 (m, 1H), 4.57 (s, 2H), 7.40 (m, 2H), 7.91 (m, 2H). ^{13}C NMR (125.7 MHz, $CDCl_3$) δ (ppm) : 14.0, 22.5, 28.1, 29.1, 31.7, 33.1, 34.9, 38.7, 81.4, 121.7, 123.3, 125.4, 126.3, 136.0, 153.2, 164.5, 166.7, 170.2, 183.0. ESI$^+$ MS (MeOH) *m/z*: 401.9 $[M+H]^+$.

methyl 4-({5-[1-(2-*tert*-butoxy-2-oxoethyl)pentyl]-1,2,4-oxadiazol-3-yl}methyl)benzoate (12l)

Procedure B2: product isolated (385 mg, 44% yield) from **3a** (2.17 mmol) after Chromatography (SiO_2, CH_2Cl_2). ^{1}H NMR (500 MHz, acetone-*d6*) δ (ppm): 0.87 (t, *J* = 7.1 Hz, 3H), 1.3 (m, 4H), 1.31 (s, 9H), 1.74 (q, *J* = 7.5 Hz, 2H), 2.69 (dd, J = 16.4 and 5.6 Hz, 1H), 2.77 (dd, J = 16.4 and 9.2 Hz, 1H), 3.43 (m, 1H), 3.88 (s, 3H), 4.17 (s, 2H), 7.47 (d, J = 8.3 Hz, 2H), 7.97 (d, *J* = 8.3 Hz, 2H). ^{13}C NMR (125.7 MHz, acetone-*d6*) δ (ppm): 14.6, 23.5, 28.6, 30.1, 33, 34.1, 35.9, 39.5, 52.8, 81.6, 118.1, 130.5, 130.9, 143, 167.5, 170.1, 171.2, 183.7. ESI$^+$ MS (MeOH) *m/z*: 402.8 $[M+H]^+$; 425.1 $[M+Na]^+$, 347.1 [M+2H-*t*Bu]$^+$ (100%).

***tert*-butyl 3-[3-(1-benzofuran-2-ylmethyl)-1,2,4-oxadiazol-5-yl]-4-cyclopentylbutanoate (13i)**

Procedure B2: product isolated (448 mg, 42% yield) from **3b** (2.62 mmol) after Chromatography on silica gel (cyclohexane:AcOEt : 95:5). ^{1}H NMR (500 MHz, $CDCl_3$) δ (ppm): 1.02 (m, 1H); 1.1 (m, 1H); 1.32 (s, 9H); 1.48 (m, 2H); 1.59 (m, 2H); 1.66 (m, 3H); 1.83 (m, 2H); 2.63 (dd, *J* = 6 and 16.2 Hz, 1H); 2.78 (dd, *J* = 8.9 and 16.2 Hz, 1H); 3.49 (m, 1H), 4.14 (s, 2H), 7.24 (t, *J* = 7.5 Hz, 1H); 7.3 (dd, *J* = 8.2 and 7.5 Hz, 1H); 7.47 (d, *J* = 8.2 Hz, 1H);

7.61 (d, J = 7.5 Hz, 1H); 7.62 (s, 1H). ^{13}C NMR (125.7 MHz, $CDCl_3$) δ (ppm): 22.7, 26.4, 29.3, 33.8, 34.1, 35.7, 39.1, 40.5, 41.1, 82.5, 112.9, 116, 121.2, 124, 125.9, 128.9, 144.1, 156.8, 169.6, 171.4, 183.8. ESI$^+$ MS (MeOH) *m/z*: 410.9 $[M+H]^+$; 433.2 $[M+Na]^+$; 355 [M+2H-*t*Bu]$^+$ (100%). El.Anal.: calcd for $C_{24}H_{30}N_2O_4$: C, 70.22, H, 7.37, N, 6.82; found: C, 70.58, H, 7.5, N, 6.78.

***tert*-butyl 3-[3-(1,3-benzodioxol-5-ylmethyl)-1,2,4-oxadiazol-5-yl]-4-cyclopentylbutanoate (13j)**

Procedure B2: product isolated (410 mg, 33% yield) from **3b** (3.01 mmol) after chromatography on silica gel (cyclohexane:AcOEt, 95:5). ^1H NMR (500 MHz, $CDCl_3$) δ (ppm): 1.03 (m, 2H), 1.31 (s, 9H), 1.46 (m, 2H), 1.56 (m, 2H), 1.64 (m, 3H), 1.80 (m, 2H), 2.59 (dd, J = 6 and 16 Hz, 1H), 2.74 (dd, J = 8.9 and 16 Hz, 1H), 3.44 (m, 1H), 3.93 (d, J = 3 Hz, 2H), 5.87 (s, 2H), 6.71 (m, 2H), 6.76 (s, 1H). ^{13}C NMR (125.7 MHz, $CDCl_3$) δ (ppm): 26.4, 29.3, 33.4, 33.7, 34, 35.6, 39, 40.4, 41, 78.1, 78.5, 78.8, 82.4, 102.3, 109.6, 110.8 , 123.4 , 130.6 , 148 , 149.2 , 170.6 , 171.3 , 183.5. ESI+ MS (MeOH) *m/z*: 414.9 $[M+H]^+$, 437.1 $[M+Na]^+$, 850.9 $[2M+Na]^+$

C. General procedure for hydroxamic acid synthesis *via* reaction with NH_2OSiMe_2*t*Bu

After hydrolysis of the *tert*-butyl ester, hydroxamic acids were prepared as previously described.[9]

1: *Hydrolysis of the tert-Butyl esters*

A solution of oxadiazole (100 mg) in CH_2Cl_2 (2mL) and TFA (2mL) was stirred at rt under argon for 6 h. CH_2Cl_2 and excess TFA were removed in vacuo to give a colourless oil that was dried under high vacuum and used in the next step without purification.

2: *Preparation of hydroxamic acids* via reaction with NH_2OSiMe_2*t*Bu

To a THF (9 mL) solution of acid (0.98 mmol, 1 equiv) were added at 0°C under argon a solution of Et_3N (1.96 mmol, 2 equiv) and *iso*-butylchloroformate(1.07 mmol, 1.1 equiv). The mixture was stirred for 1 h and then filtered. *O*-(*tert*-butyldimethylsilyl)hydroxylamine (3.1

mmol, 3.2 equiv) was added and the reaction was allowed to warm to rt and stirred overnight. Then, a solution of TBAF (3.1 mmol, 3.2 equiv) was added and the reaction further stirred for 3 h. The solvent was evaporated to dryness and the residue was dissolved in AcOEt and successively washed with saturated aqueous $NaHCO_3$, HCl 0.1N and brine. The organic layer was then dried over $MgSO_4$, filtered and condensed. The residue was purified by chromatography on silica gel eluted with CH_2Cl_2/MeOH to give a yellow oil. Trituration of the oil in diethyl ether at 0°C led to precipitation of the final product as a white powder that was filtered and washed with pentane and dried.

***N*-hydroxy-3-[3-(4-nitrophenyl)-1,2,4-oxadiazol-5-yl]heptanamide (AT001)** : 100mg (35% yield) prepared from **12a** (0.84 mol, 315 mg) - chromatography (SiO_2, CH_2Cl_2/MeOH: 95/5). ^{1}H NMR (250 MHz, acetone-*d6*) δ (ppm): 0.87 (t, J = 6.5 Hz, 3H), 1.33 (m, 4H), 1.83 (m,2H), 2.72 (m, 2H), 3.65 (m, 1H), 8.31 (d, *J* = 9Hz, 2H), 8.41 (d, *J* = 9Hz, 2H), 10.20 (s, 1H). ^{13}C NMR (125.7 MHz, acetone-*d6*) δ (ppm): 184.7, 168.6, 168.0, 151.0, 134.3, 129.8, 125.6, 36.7, 36.1, 34.1, 31.7, 23.5, 14.6. ESI$^+$ MS (MeOH) *m/z*: 335.1 $[M+H]^+$. El. Anal.: calcd for $C_{15}H_{18}N_4O_5$ C,54.58, H,5.47, N,16.31; found: C,54.83, H,5.72, N,16.31.

3-[3-(4-fluorobenzyl)-1,2,4-oxadiazol-5-yl]-*N*-hydroxyheptanamide (AT002) : 137 mg (43.5% yield) prepared from **12b** (0.97 mmol, 350 mg) chromatography (SiO_2, CH_2Cl_2/MeOH: 95/5). ^{1}H NMR (500 MHz, acetone-*d6*) δ (ppm): 0.85 (t, *J* = 7 Hz, 3H), 1.26 (m, 4H), 1.73 (m, 2H), 2.57 (m, 1H), 2.64 (m, 1H), 3.52 (tt, *J* = 6.3 and 8 Hz, 1H), 4.07 (s, 2H), 7.09 (*t*, J = 8.7 Hz, 2H), 7.37 (m, 2H), 8.02 (s, 1H), 10.01 (s, 1H). ^{13}C NMR (125.7 MHz, acetone-*d6*) δ (ppm):14.1, 23.0, 29.6, 31.8, 33.6, 35.4, 36.2, 116 (d, J = 21.5 Hz),131.7 (d, J = 8.2Hz), 133.3, 162.8 (d, J = 242 Hz) , 168.0, 170.0, 183.0. El. Anal.: calcd for $C_{16}H_{20}N_3O_3F{\cdot}0.3H_2O$ C, 58.8, H, 6.35, N, 12.85; found: C, 59.25, H, 6.32, N, 12.72.

***N*-hydroxy-3-(3-phenyl-1,2,4-oxadiazol-5-yl)heptanamide (AT006)** : 80 mg (45% yield) prepared from **12c** (0.6 mmol, 198 mg) chromatography (SiO_2, CH_2Cl_2:MeOH: 95:5). ^{1}H NMR

(500 MHz, Acetone--*d6*) δ (ppm): 0.9 (t, *J* = 6.6 Hz, 3H), 1.34 (4H, m), 1.84 (2H, m), 2.65 (dd, *J* = 6.5 and 15.5 Hz, 1H), 2.77 (dd, *J* = 8.5 and 15.5 Hz, 1H), 3.64 (q, *J* = 7.5 Hz, 1H), 7.57 (m, 2H), 8.08 (m, 2H), 9.12 (s, 1H), 10.05 (s, 1H). ^{13}C NMR (125.7 MHz, acetone-*d6*) δ (ppm): 14.6, 23.5, 30.6, 34.1, 35.9, 36.8, 128.5, 130.3, 132.5, 168.5, 169.3, 178.2, 183.7. ESI$^+$ HRMS: calcd for $[M+Na]^+$ $C_{15}H_{19}N_3O_4Na$ 312.1324; found 312.1313.

***N*-hydroxy-3-{3-[4-(trifluoromethyl)benzyl]-1,2,4-oxadiazol-5-yl}heptanamide (AT008)** : 63 mg (50% yield) prepared from **12d** (0.33 mmol, 140 mg) - chromatography (SiO_2, CH_2Cl_2/MeOH: 95/5). ^{1}H NMR (500 MHz, acetone-*d6*) δ (ppm) : 0.85 (t, *J* = 7.2 Hz, 3H), 1.27 (4H, m), 1.74 (2H, m), 2.57 (dd, *J* = 7 and 15 Hz, 1H), 2.66 (dd, *J* = 8 and 15 Hz, 1H), 3.54 (m, 1H), 4.21 (s, 2H), 7.58 (d, *J* = 7.7 Hz, 2H), 7.7 (d, *J* = 7.7 Hz, 2H), 8.37 (s, 1H), 10.06 (s, 1H). ^{13}C NMR (125.7 MHz, acetone-*d6*) δ (ppm): 14.6, 23.5, 30.1, 32.8, 34, 35.8, 36.7, 125.9 (q, *J* = 270 Hz), 126.7 (q, *J* = 4.2 Hz), 130 (q, *J* = 31.8 Hz), 131, 142.4, 168.6, 169.9, 183.7. ESI$^+$ HRMS: calcd for $[M+Na]^+$ $C_{17}H_{20}N_3O_3NaF_3$ 394.1354; found 394.1361.

***N*-hydroxy-3-{3-[4-(trifluoromethyl)phenyl]-1,2,4-oxadiazol-5-yl}heptanamide (AT011)** : 100 mg (48% yield) prepared from **12e** (0.58 mmol, 233 mg) - chromatography (SiO_2, CH_2Cl_2:MeOH, 95:5), yield 48%. ^{1}H NMR (250 MHz, Acetone-*d6*) δ (ppm): 0.87 (t, *J* = 6.7 Hz, 3H), 1,32 (m, 4H), 1.83 (m, 2H), 2.69 (m, 2H), 3.65 (m, 1H), 7.92 (m, 2H), 7.99 (s, 1H), 8,29 (m, 2H), 10 (s, 1H). ^{13}C NMR (125.7 MHz, acetone--*d6*) δ (ppm): 14.2, 23.0, 29.7, 33.6, 35.6, 36.3, 125.0 (q, *J* = 273 Hz), 126.9, 128.8, 131.8, 133.1 (q, *J* = 32.3Hz), 167.9, 168.4, 183.8. ESI$^+$ HRMS: calcd for $[M+H]^+$ $C_{16}H_{17}N_3O_3F_3$ 356.1222; found 356.1236

***N*-hydroxy-3-[3-(4-methylbenzyl)-1,2,4-oxadiazol-5-yl]heptanamide (AT012)** : 202 mg (54% yield) prepared from **12f** (1.18 mmol, 423 mg) - chromatography (SiO_2, CH_2Cl_2/MeOH, 95/5). ^{1}H NMR (250 MHz, acetone-*d6*) δ (ppm): 0.83 (t, *J* = 6.7 Hz, 3H), 1.23 (m, 4H), 1.71 (m, 2H), 2.28 (s, 3H), 2.56 (m, 2H), 3.50 (m, 1H), 3.98 (s, 2H), 7.15 (m, 4H), 7.96 (s, 1H), 9.99 (s, 1H). ^{13}C NMR (125.7 MHz, acetone-D6) δ (ppm): 14.2, 21.1, 23.0, 29.7, 32.2, 33.5,

35.5, 36.2, 129.6, 130.0, 134.1, 137.1, 170.2, 182.8. ESI$^+$ HRMS: calcd for $[M+H]^+$ $C_{17}H_{24}N_3O_3$ 318.1818; found 318.1825.

***N*-hydroxy-3-{3-[4-(trifluoromethoxy)benzyl]-1,2,4-oxadiazol-5-yl}heptanamide (AT013)** : 140 mg (49% yield) prepared from **12g** (0.74 mmol, 317 mg) - chromatography (SiO_2, CH_2Cl_2:MeOH, 97:3). ^{1}H NMR (500 MHz, acetone-*d6*): 0.83(t, J = 6.7 Hz, 3H), 1.23(m, 4H), 1.71 (m, 2H), 2.54 (m, 2H), 3.51 (m, 1H), 4.11 (s, 2H), 7.37 (m, 4H), 7.98 (s, 1H), 10.0 (s, 1H) ^{13}C NMR (125.7 MHz, acetone-*d6*) δ (ppm): 14.1, 23.0, 29.7, 31.9, 33.5, 35.4, 36.3, 121.5 (q, *J* = 254 Hz), 122.0, 131.6, 136.6, 148.9, 168.2, 169.8, 183.1. ESI$^+$ HRMS: calcd for $[M+H]^+$ $C_{17}H_{21}N_3O_4F_3$ 388.1484, found 388.1502.

3-[3-(biphenyl-4-ylmethyl)-1,2,4-oxadiazol-5-yl]-*N*-hydroxyheptanamide (AT014): 167 mg (45% yield) prepared from **12h** (0.987 mmol, 415 mg) - chromatography (SiO_2, CH_2Cl_2:MeOH, 95:5). ^{1}H NMR (250 MHz, acetone-*d6*) δ (ppm): 0.84 (t, *J* = 6.9 Hz, 3H), 1.26 (m, 4H), 1.74 (m, 2H), 2.59 (m, 2H), 3.54 (m, 1H), 4.10 (s, 2H), 7.53 (m, 9H), 7.96 (s, 1H), 10.01 (s, 1H). ^{13}C NMR (125.7 MHz, acetone-*d6*) δ (ppm) : 14.1, 23.0, 29.7, 32.2, 33.5, 35.4, 36.2, 127.6, 127.9, 128.0, 129.6, 130.2, 136.2, 140.4, 141.4, 168.5, 170.0, 182.8. ESI$^+$ HRMS: calcd for $[M+H]^+$ $C_{22}H_{26}N_3O_4$ 380.1974; found 380.1985.

3-[3-(1-benzofuran-2-ylmethyl)-1,2,4-oxadiazol-5-yl]-*N*-hydroxyheptanamide (AT015): 254 mg (63% yield) prepared from **12i** (1.17 mmol, 452 mg) - chromatography (SiO_2, CH_2Cl_2:MeOH, 97:3). ^{1}H NMR (250 MHz, acetone-*d6*) δ (ppm): 0.81 (t, *J* = 6 Hz, 3H), 1.26 (m, 4H), 1.73 (m, 2H), 2.60 (m, 2H), 3.52 (m, 1H), 4.17 (s, 2H), 7.50 (m, 5H), 7.96 (s, 1H), 10.01 (s, 1H). ^{13}C NMR (125.7 MHz, acetone-*d6*) δ (ppm): 14.1, 21.5, 23.0, 29.6, 33.5, 35.4, 36.2, 112.1, 116.1, 120.9, 123.5, 125.4, 128.5, 143.9, 156.2, 168.5, 169.2, 183.0. ESI$^+$ HRMS: calcd for $[M+H]^+$ $C_{18}H_{22}N_3O_4$ 344.1610; found 344.1608.

3-[3-(1,3-benzodioxol-5-ylmethyl)-1,2,4-oxadiazol-5-yl]-*N*-hydroxyheptanamide (AT016) : 174.5 mg (49% yield) prepared from **12j** (1.025 mmol, 398 mg) - chromatography (SiO_2,

CH_2Cl_2:MeOH, 97:3). ^{1}H NMR (250 MHz, acetone-*d6*) δ (ppm): 0.84(t, *J* = 6.7 Hz, 3H), 1.26 (m, 4H), 1.71 (m, 2H), 2.57 (m, 2H), 3.51 (m, 1H), 3.96 (s, 2H), 5.97 (s, 2H), 6.8 (m, 3H), 7.97 (s, 1H), 10.0 (s, 1H). ^{13}C NMR (125.7 MHz, acetone-*d6*) δ (ppm): 14.1, 23.0, 29.5, 32.2, 33.4, 35.3, 36.2, 102, 108.9, 110.1, 122.8, 130.6, 147.5, 148.7, 168.6, 170.1, 182.7. ESI$^+$ HRMS: calcd for $[M+H]^+$ $C_{17}H_{22}N_3O_5$ 348.1559, found 348.1550.

3-[3-(1,3-benzothiazol-2-ylmethyl)-1,2,4-oxadiazol-5-yl]-*N*-hydroxyheptanamide (AT017) : 25 mg (17% yield) prepared from **12k** (0.4 mmol, 140 mg) - chromatography (SiO_2, CH_2Cl_2:MeOH, 95:5). ^{1}H NMR (250 MHz, acetone-*d6*) δ (ppm): 0.83 (t, *J* = 6.8 Hz, 3H), 1.26 (m, 4H), 1.75 (m, 2H), 2.62 (m, 2H), 3.56 (m, 1H), 4.62 (s, 2H), 7.47 (m, 2H), 7.99 (m, 2H), 7.96 (s, 1H), 10.01 (s, 1H). ^{13}C NMR (125.7 MHz, acetone- *d6*) δ (ppm): 14.2, 23.0, 29.7, 31.9, 33.6, 35.4, 36.3, 122.8, 123.7, 126.2, 127.0, 136.9, 154.1, 165.9, 167.7, 168.0, 183.6. ESI$^+$ HRMS: calcd for $[M+H]^+$ $C_{17}H_{21}N_4O_3S$ 361.1334; found 361.1351

Methyl-4-[(5-{1-[2-(hydroxyamino)-2-oxoethyl]pentyl}-1,2,4-oxadiazol-3-yl)methyl]benzoate (AT018) : 75 mg (17% yield) prepared from **12l** (1.21 mmol, 490 mg) - chromatography (SiO_2, CH_2Cl_2:MeOH, 98:4), yield 16%. ^{1}H NMR (500 MHz, acetone-*d6*) δ (ppm): 0.83 (t, *J* = 7.2 Hz, 3H), 1.24-1.28 (m, 4H), 1.72 (m, 2H), 2.59 (m, 2H), 3.52 (m, 1H), 3.86 (s, 3H), 4.15 (s, 2H), 7.45 (d, *J* = 8.2 Hz, 2H), 7.95 (d, *J* = 8.2 Hz, 2H), 8.29 (s, 1H), 10.08 (s, 1H). ^{13}C NMR (125.7 MHz, acetone-D6) δ (ppm): 13.1, 22, 28.7, 31.5, 32.5, 34.4, 35.2, 51.4, 128.9, 129, 129.5, 141.6, 166.1, 167.1, 168.5, 182.1. ESI$^+$ HRMS: calcd for $[M+Na]^+$ $C_{18}H_{23}N_3O_5Na$ 384.1535; found 384.1525.

3-[3-(1-benzofuran-2-ylmethyl)-1,2,4-oxadiazol-5-yl]-4-cyclopentyl-*N*-hydroxy butanamide (AT019) : 290 mg (72% yield) prepared from **13i** (1.09 mmol, 450 mg) - chromatography (SiO_2, CH_2Cl_2:MeOH, 98:2). IR (neat, ν □cm^{-1}): 3211, 2948, 2867, 1655, 1577. ^{1}H NMR (500 MHz, acetone-*d6*) δ (ppm): 0.99 (m, 1H), 1.09 (m, 1H), 1.43 (m, 2H), 1.62 (m, 5H), 1.79 (m, 2H), 2.58 (dd, *J* = 7 and 14.7 Hz, 1H), 2.67 (dd, *J* = 8 and 14.7 Hz, 1H),

3.58 (m, 1H), 4.18 (s, 2H), 7.24 (t, *J* = 7.4 Hz, 1H), 7.33 (dd, *J* = 8.2 and 7.4 Hz, 1H), 7.51 (d, *J* = 8.2 Hz, 1H), 7.65 (d, *J* = 7.4 Hz, 1H), 7.78 (s, 1H), 8.5 (s, 1H), 10.15 (s, 1H). ^{13}C NMR (125.7 MHz, acetone-*d6*) δ (ppm): 22.1, 26.1, 26.2, 33.3, 33.9, 35.5, 37.3, 39.1, 40.9, 112.6, 116.7, 121.4, 124, 125.9, 129, 144.5, 156.7, 168.8, 169.8, 183.7. ESI$^+$ MS (MeOH) *m/z*: 370 $[M+H]^+$, 738.7 $[2M+H]^+$, 1130.1 $[3M+Na]^+$. El. Anal.: calcd for $C_{20}H_{23}N_3O_4$ C, 65.03, H, 6.28, N, 11.37; found: C,64.33, H,6.37, N,11.22.

3-[3-(1,3-benzodioxol-5-ylmethyl)-1,2,4-oxadiazol-5-yl]-4-cyclopentyl-*N*-hydroxy butanamide (AT020) : 310 mg (61% yield) prepared from **13j** (1.37 mmol, 569 mg) – chromatography (SiO_2, CH_2Cl_2:MeOH, 98:2). IR (neat, ν □cm^{-1}): 3217, 2950, 2869, 1659, 1575. ^{1}H NMR (500 MHz, acetone-*d6*) δ (ppm): 1.02 (m, 1H), 1.12 (m, 1H), 1.48 (m, 2H), 1.64 (m, 5H), 1.80 (m, 2H), 2.55 (dd, *J* = 7, 15 Hz, 2H), 2.64 (dd, *J* = 7.8 and 15 Hz, 1H), 3.57 (m, 1H), 3.98 (s, 2H), 5.98 (s, 2H), 6.79 (m, 2H), 6.84 (s, 1H), 8.09 (s, 1H), 10.03 (s, 1H). ^{13}C NMR (125.7 MHz, acetone-*d6*) δ (ppm): 26.1, 26.2, 32.7, 33.4, 33.9, 35.4, 37.2, 39.1, 40.9, 102.5, 109.4, 110.6, 123.3, 131.3, 148, 149.3, 168.5, 170.7, 183.5. ESI$^+$ MS (MeOH) *m/z*: 374 $[M+H]^+$, 1142 $[3M+Na]^+$. El. Anal.: calcd for $C_{19}H_{23}N_3O_5$: C,61.11, H,6.21, N,11.25; found: C,60.86, H,6.47, N,11.03.

4-[(5-{1-[2-(hydroxyamino)-2-oxoethyl]pentyl}-1,2,4-oxadiazol-3-yl)methyl]benzoic acid (AT021)

140 mg (33% yield) prepared from **12l** (1.21 mmol, 490 mg) – chromatography (SiO_2, CH_2Cl_2:MeOH: 98:4). IR (neat, ν □cm^{-1}): 3240, 3206, 2957, 2928, 2868, 1682, 1633, 1581.^{1}H NMR (500 MHz, acetone-*d6*) δ (ppm): 0.85 (t, J=7.2Hz, 3H), 1.17-1.34 (m, 4H), 1.74 (m, 2H), 2.60 (m, 2H), 3.53 (tt, *J* = 6.3 and 8 Hz, 1H), 4.16 (s, 2H), 7.45 (d, *J* = 8.2 Hz, 2H), 7.93 (d, *J* = 8.3 Hz, 2H), 10.05 (s, 1H). ^{13}C NMR (125.7 MHz, acetone-*d6*) δ (ppm): 14.6, 23.5, 30.6, 33, 34, 35.8, 36.7, 130.4, 130.6, 131.2, 142.9, 168, 168.5, 170, 183.6. ESI$^+$ MS (MeOH) *m/z*: 347.9

$[M+H]^+$, 694.8 $[2M+H]^+$, 716.9 $[2M+Na]^+$. ESI^+ HRMS: calcd for $[M+H]^+$ $C_{17}H_{22}N_3O_5$ 348.1559; found: 348.1551.

References

1. Evans, D. A.; Britton, T. C.; Ellman, J. A. Contrasteric carboximide hydrolysis with lithium hydroperoxide. *Tetrahedron Lett* **1987,** 28.

2. Levy, D. E.; Lapierre, F.; Liang, W.; Ye, W.; Lange, C. W.; Li, X.; Grobelny, D.; Casabonne, M.; Tyrrell, D.; Holme, K.; Nadzan, A.; Galardy, R. E. Matrix metalloproteinase inhibitors: a structure-activity study. *J Med Chem* **1998,** 41, 199-223.

3. Hilpert, H. Practical approaches to the matrix metalloproteinase inhibitor Trocade® (Ro 32-3555) and to the TNF-α converting enzyme inhibitor Ro 32-7315. . *Tetrahedron* **2001,** 57, 7675–7683.

4. Phillips, A. J.; Uto, Y.; Wipf, P.; Reno, M. J.; Williams, D. R. Synthesis of functionalized oxazolines and oxazoles with DAST and Deoxo-Fluor. *Org Lett* **2000,** 2, 1165-8.

5. Bailey, S.; Fish, P. V.; Billotte, S.; Bordner, J.; Greiling, D.; James, K.; McElroy, A.; Mills, J. E.; Reed, C.; Webster, R. Succinyl hydroxamates as potent and selective non-peptidic inhibitors of procollagen C-proteinase: design, synthesis, and evaluation as topically applied, dermal anti-scarring agents. *Bioorg Med Chem Lett* **2008,** 18, 6562-7.

6. Fish, P. V.; Allan, G. A.; Bailey, S.; Blagg, J.; Butt, R.; Collis, M. G.; Greiling, D.; James, K.; Kendall, J.; McElroy, A.; McCleverty, D.; Reed, C.; Webster, R.; Whitlock, G. A. Potent and selective nonpeptidic inhibitors of procollagen C-proteinase. *J Med Chem* **2007,** 50, 3442-56.

7. La Motta, C.; Sartini, S.; Salerno, S.; Simorini, F.; Taliani, S.; Marini, A. M.; Da Settimo, F.; Marinelli, L.; Limongelli, V.; Novellino, E. Acetic acid aldose reductase inhibitors bearing a five-membered heterocyclic core with potent topical activity in a visual impairment rat model. *J Med Chem* **2008,** 51, 3182-93.

8. Augustine, J. K.; Vairaperumal, V.; Narasimhan, S.; Alagarsamy, P.; Radhakrishnan, A. Propylphosphonic anhydride (T3P®): an efficient reagent for the one-pot synthesis of 1,2,4-oxadiazoles, 1,3,4-oxadiazoles, and 1,3,4-thiadiazoles. *Tetrahedron* **2009,** 65, 9989–9996.

9. Huguet, F.; Melet, A.; Alves de Sousa, R.; Lieutaud, A.; Chevalier, J.; Maigre, L.; Deschamps, P.; Tomas, A.; Leulliot, N.; Pages, J. M.; Artaud, I. Hydroxamic acids as potent inhibitors of Fe(II) and Mn(II) E. coli methionine aminopeptidase: biological activities and X-ray structures of oxazole hydroxamate-EcMetAP-Mn complexes. *ChemMedChem* **2012,** 7, 1020-30.

10. Leite, L. F.; Ramos, M. N.; da Silva, J. B.; Miranda, A. L.; Fraga, C. A.; Barreiro, E. J. Synthesis and analgesic profile of novel N-containing heterocycle derivatives: arylidene 3-phenyl-1,2,4-oxadiazole-5-carbohydrazide. *Farmaco* **1999,** 54, 747-57.

11. Kitamura, S.; Fukushi, H.; Miyawaki, T.; Kawamura, M.; Konishi, N.; Terashita, Z.; Naka, T. Potent dibasic GPIIb/IIIa antagonists with reduced prolongation of bleeding time: synthesis and pharmacological evaluation of 2-oxopiperazine derivatives. *J Med Chem* **2001,** 44, 2438-50.

NMR spectra of all new compounds

AT002

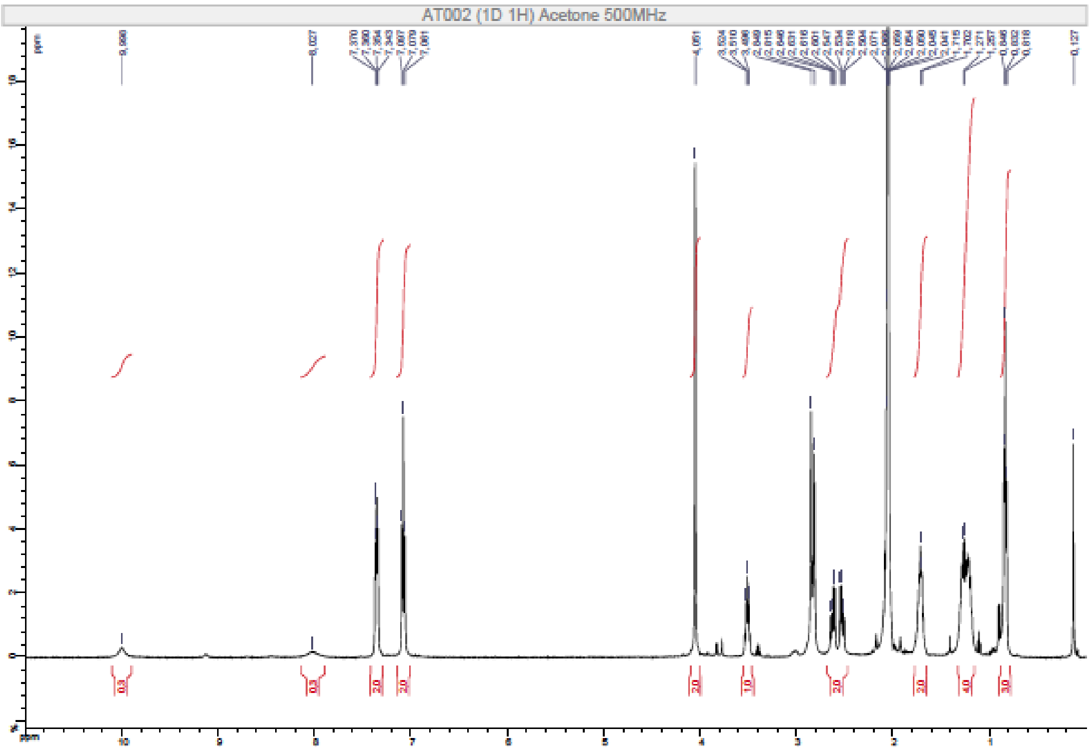

AT003

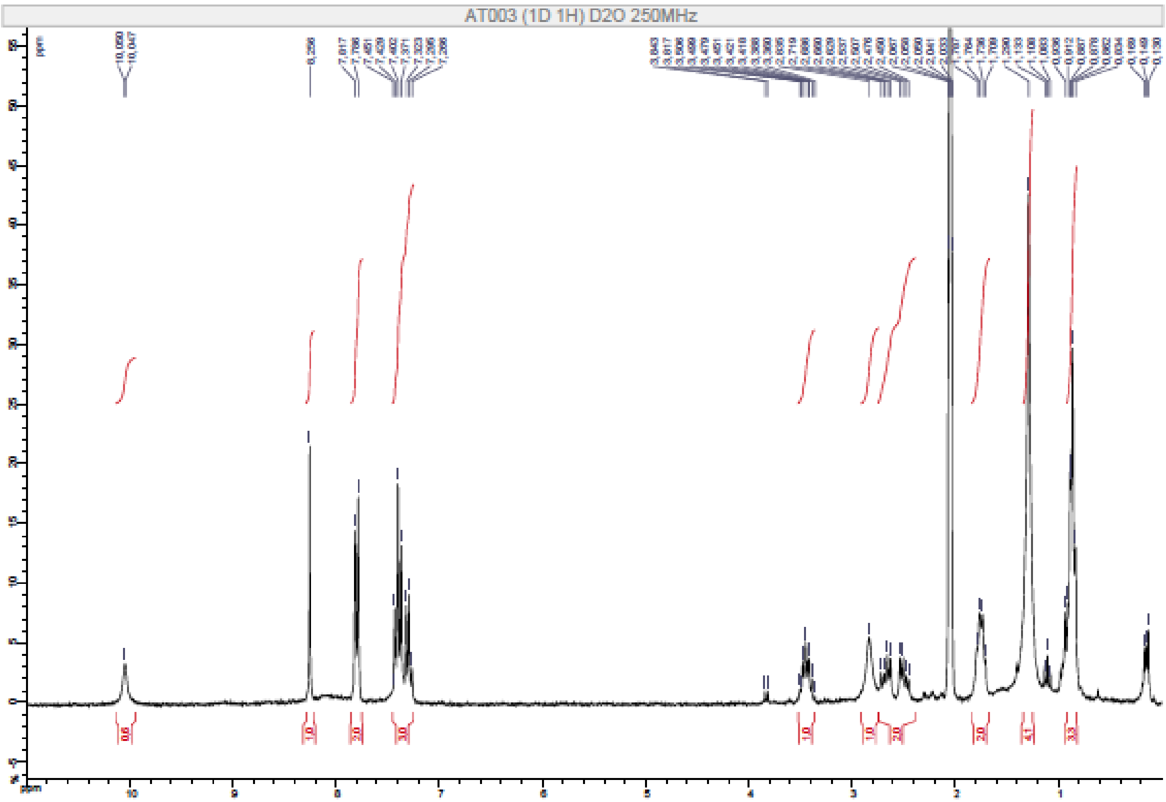

AT004

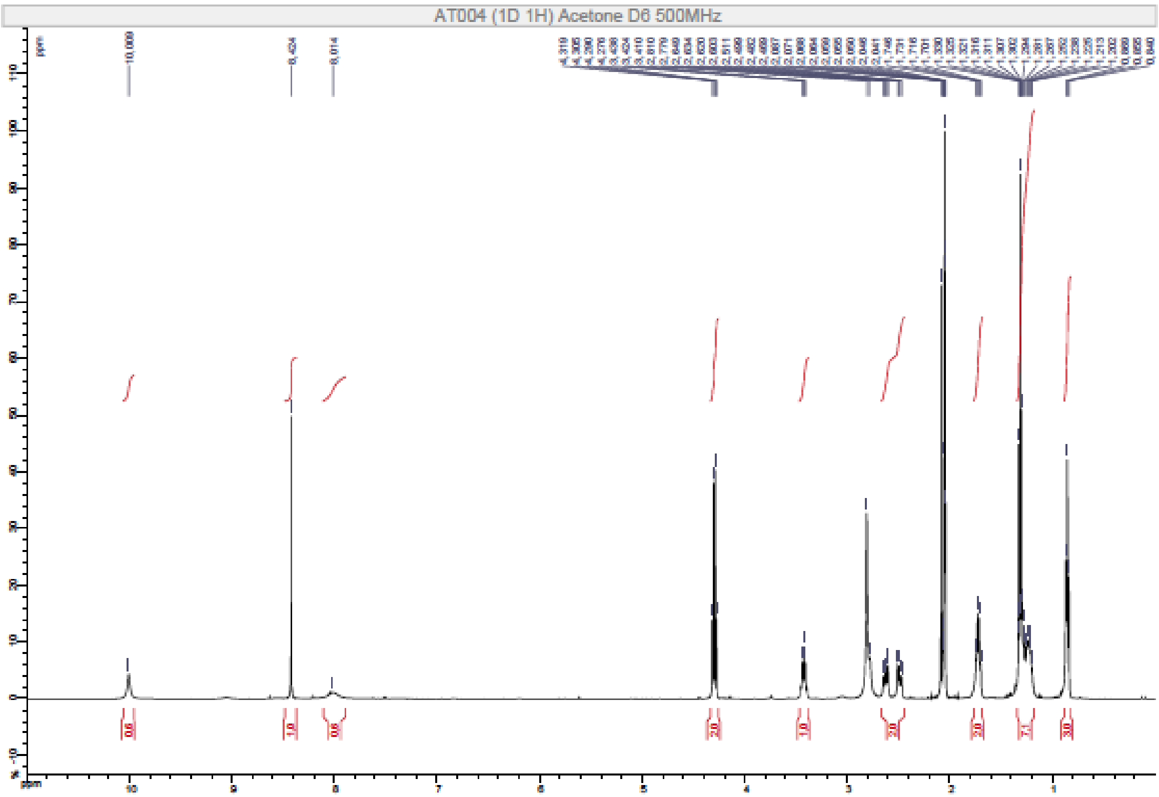

AT007

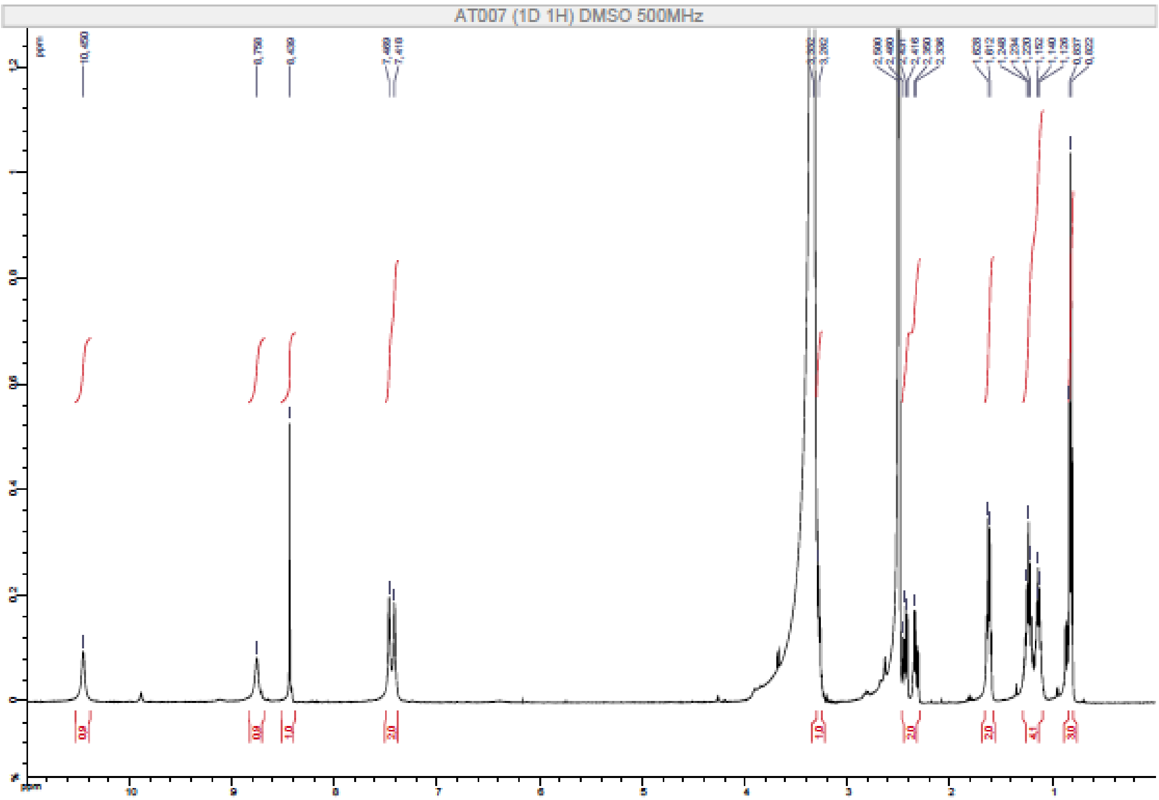

AT008

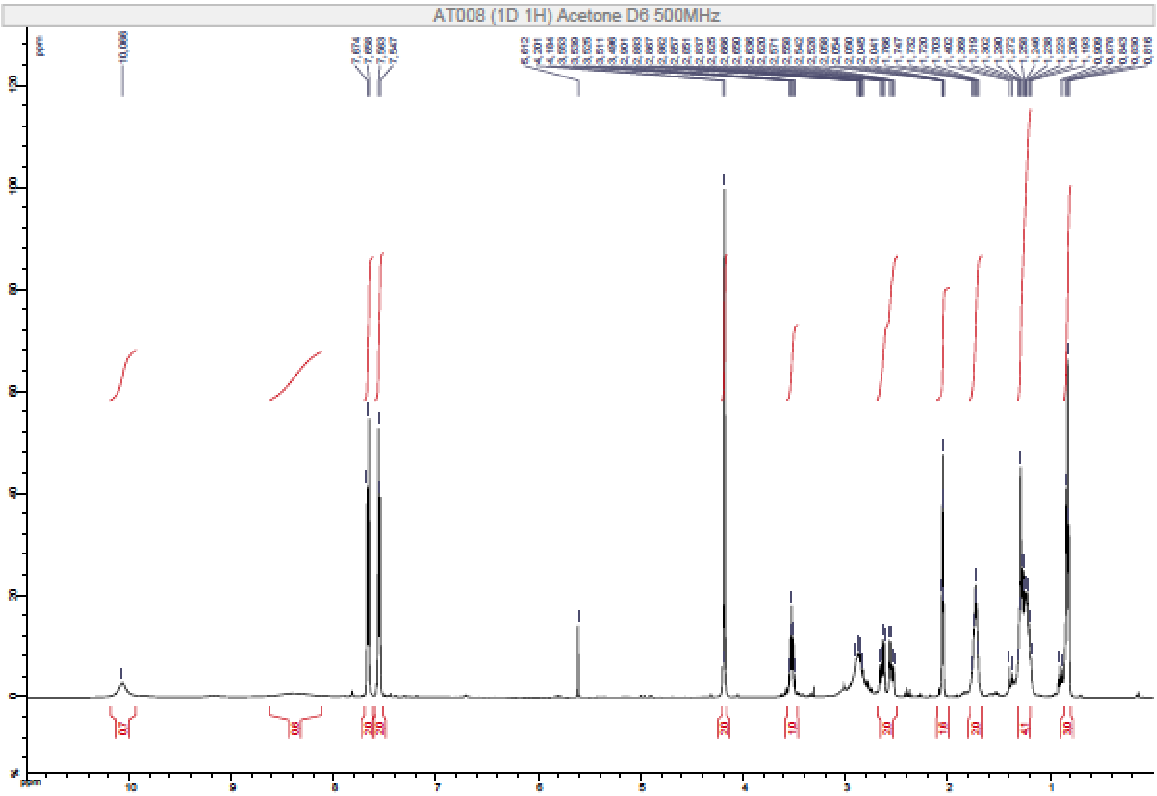

AT009

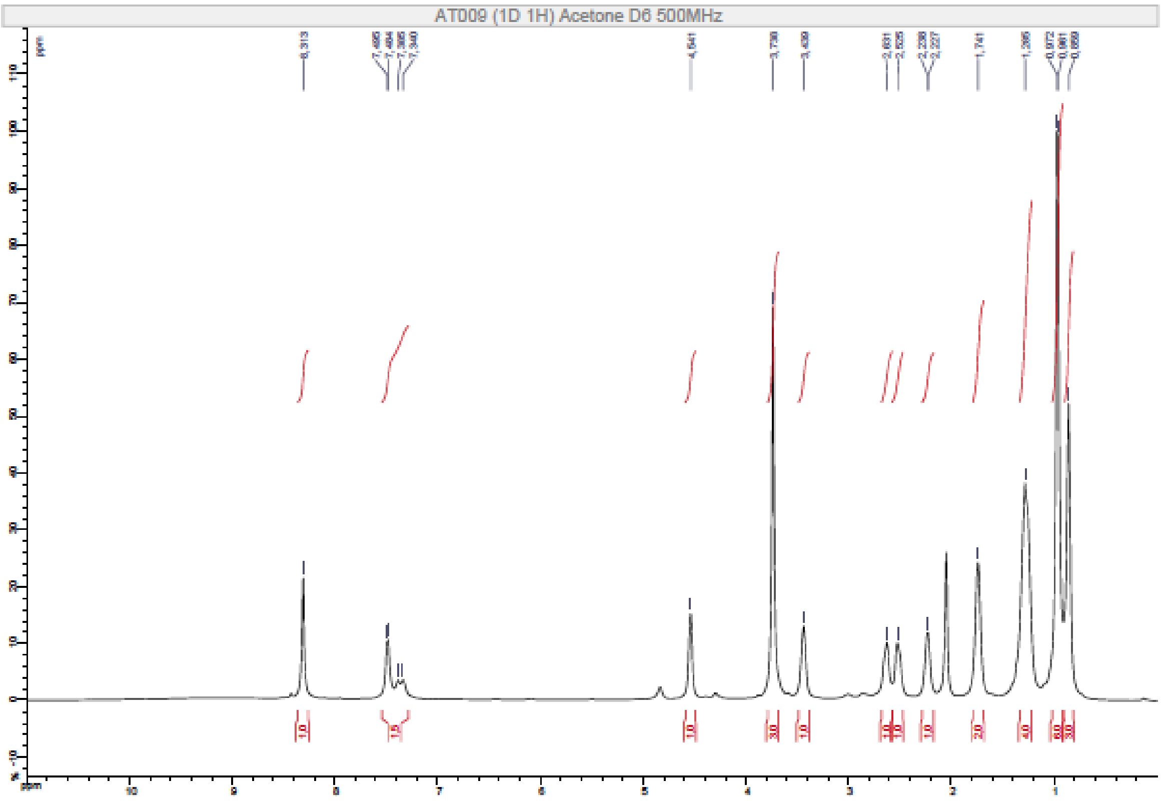

AT010

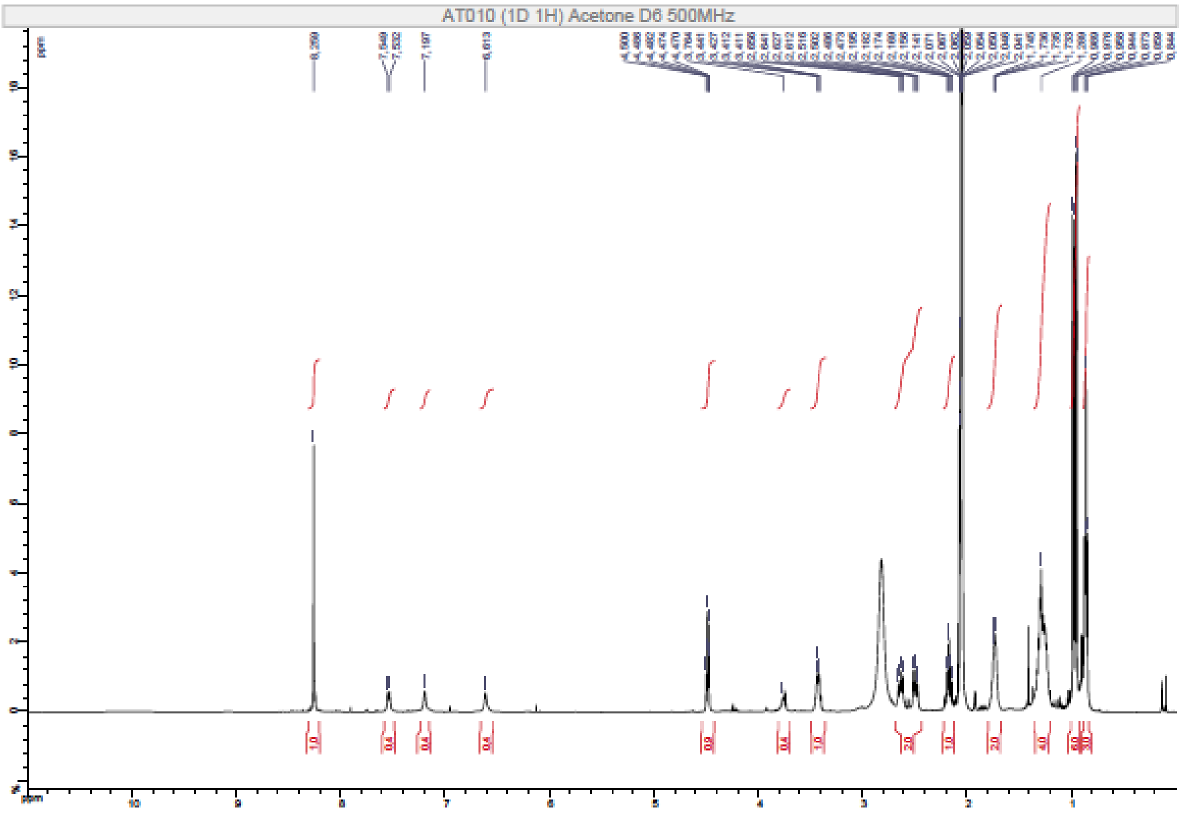

AT011

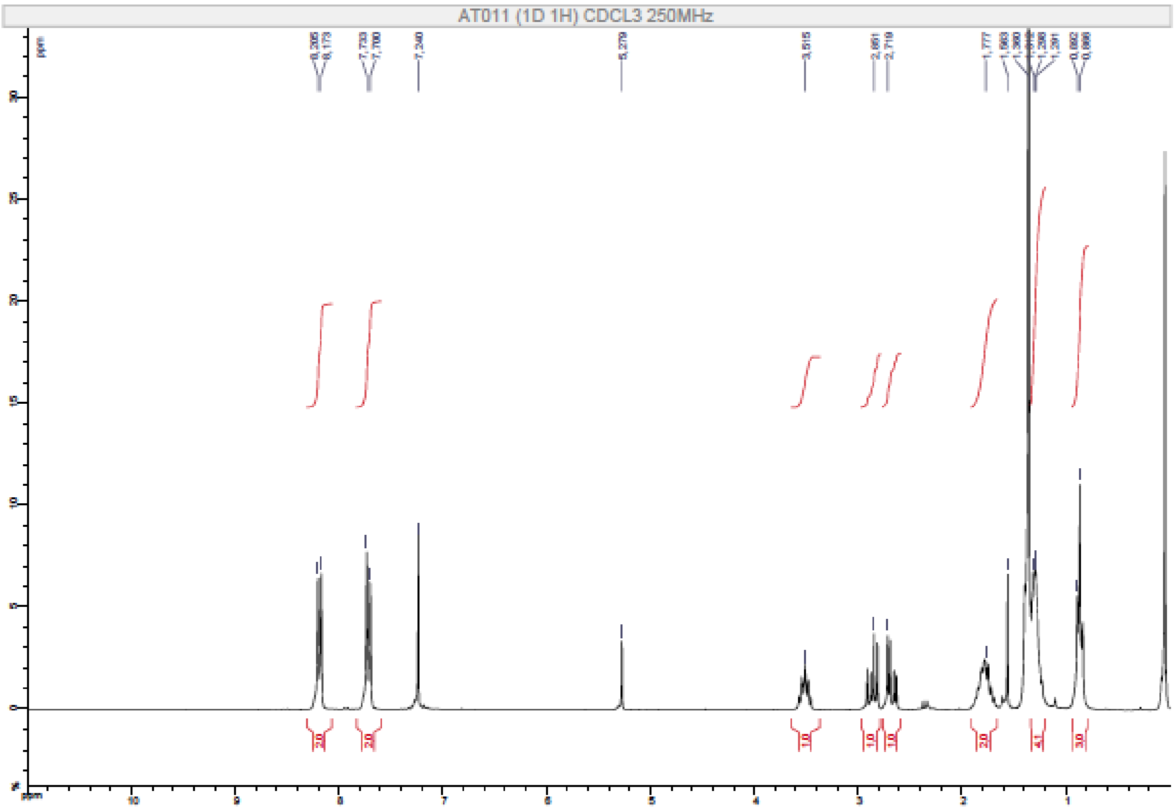

AT012

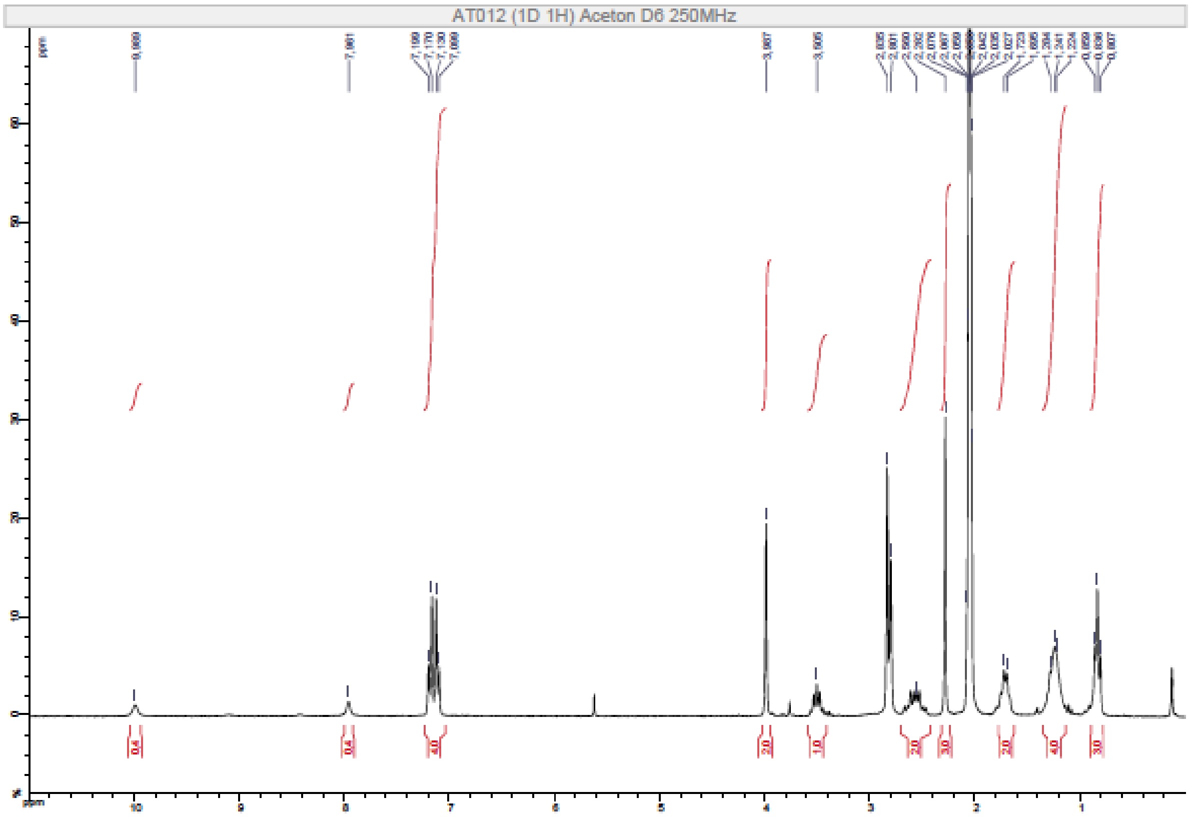

AT013

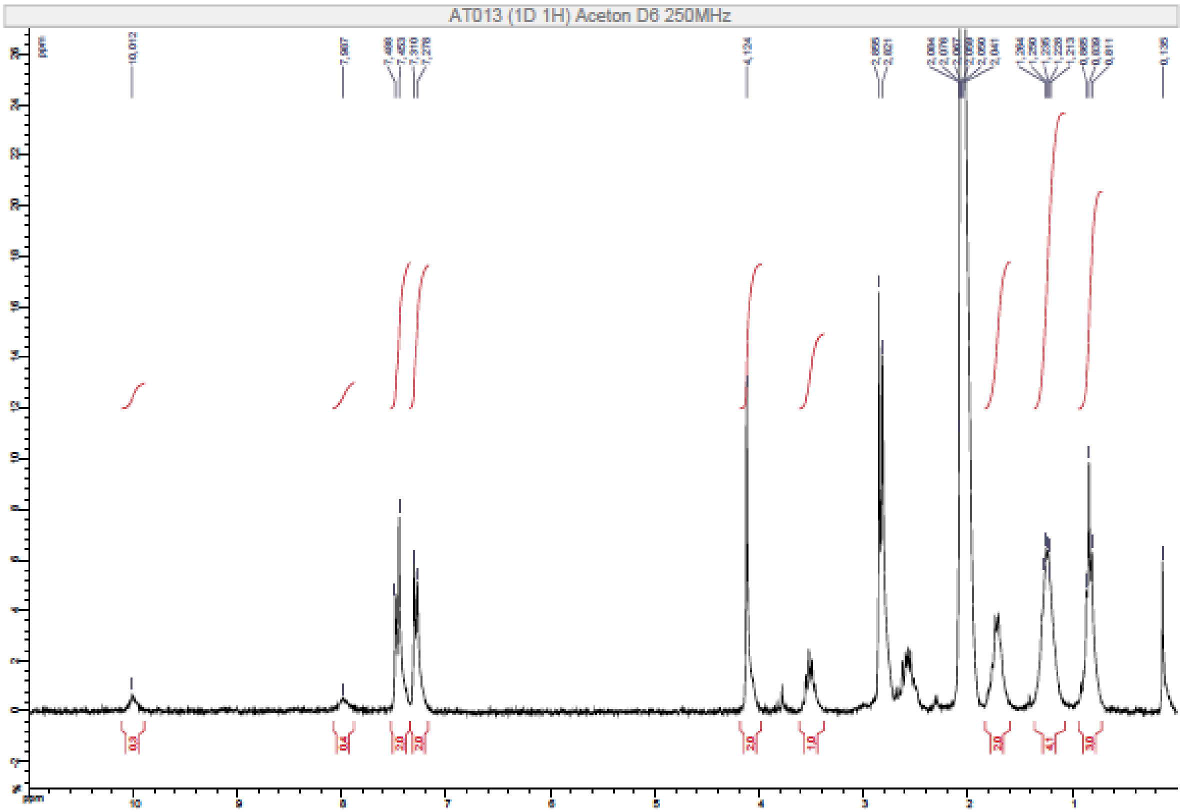

AT014

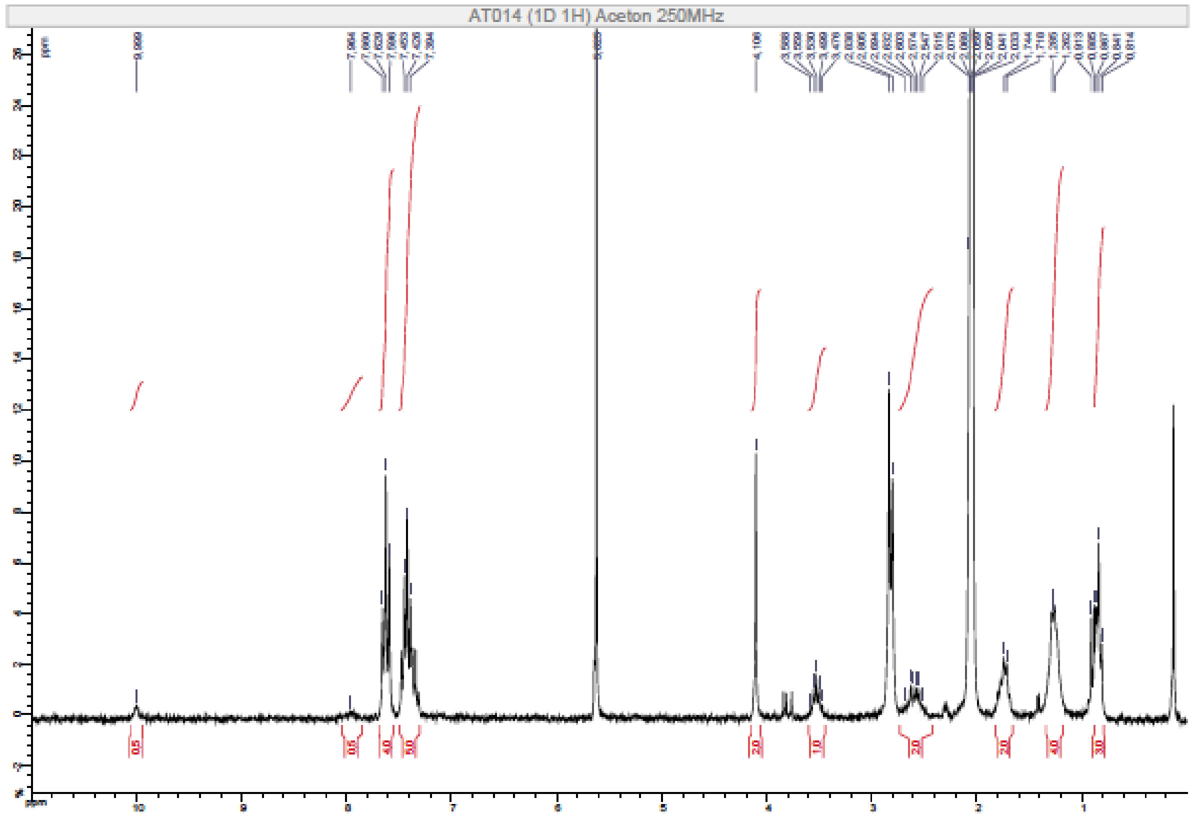

AT015

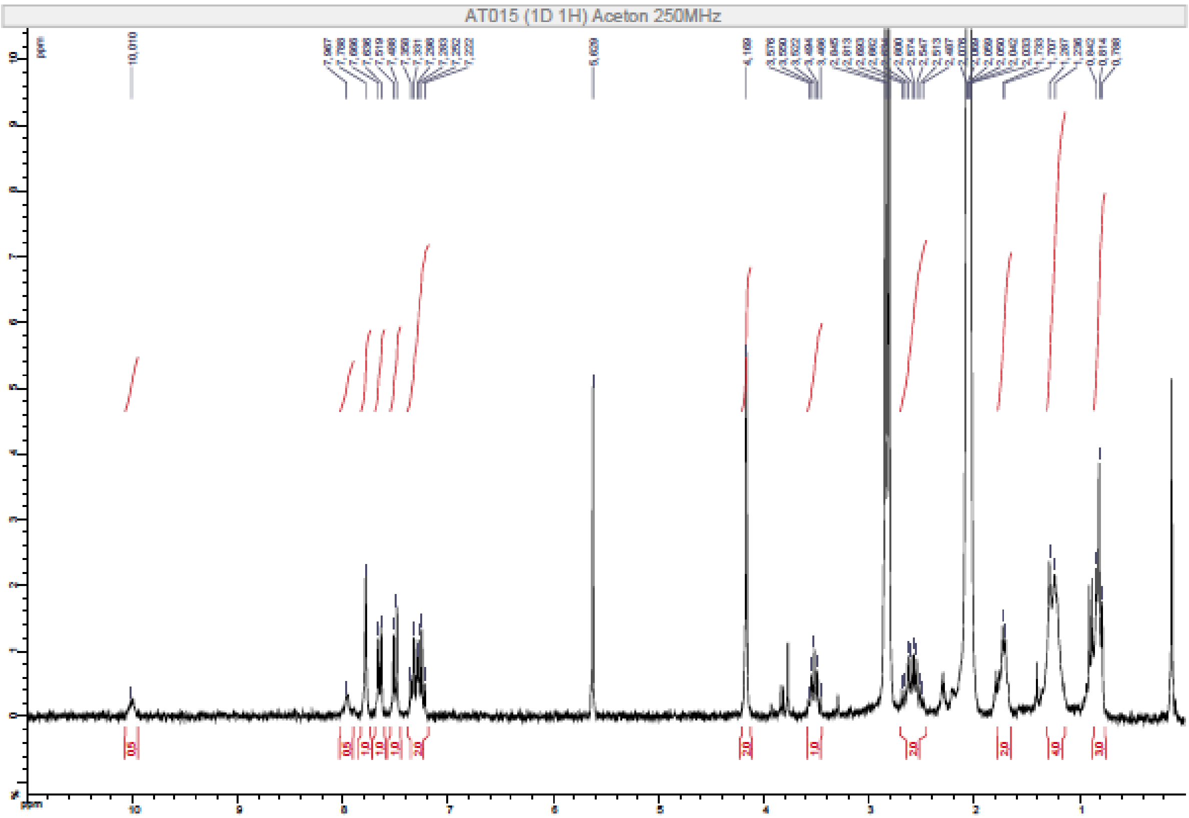

AT016

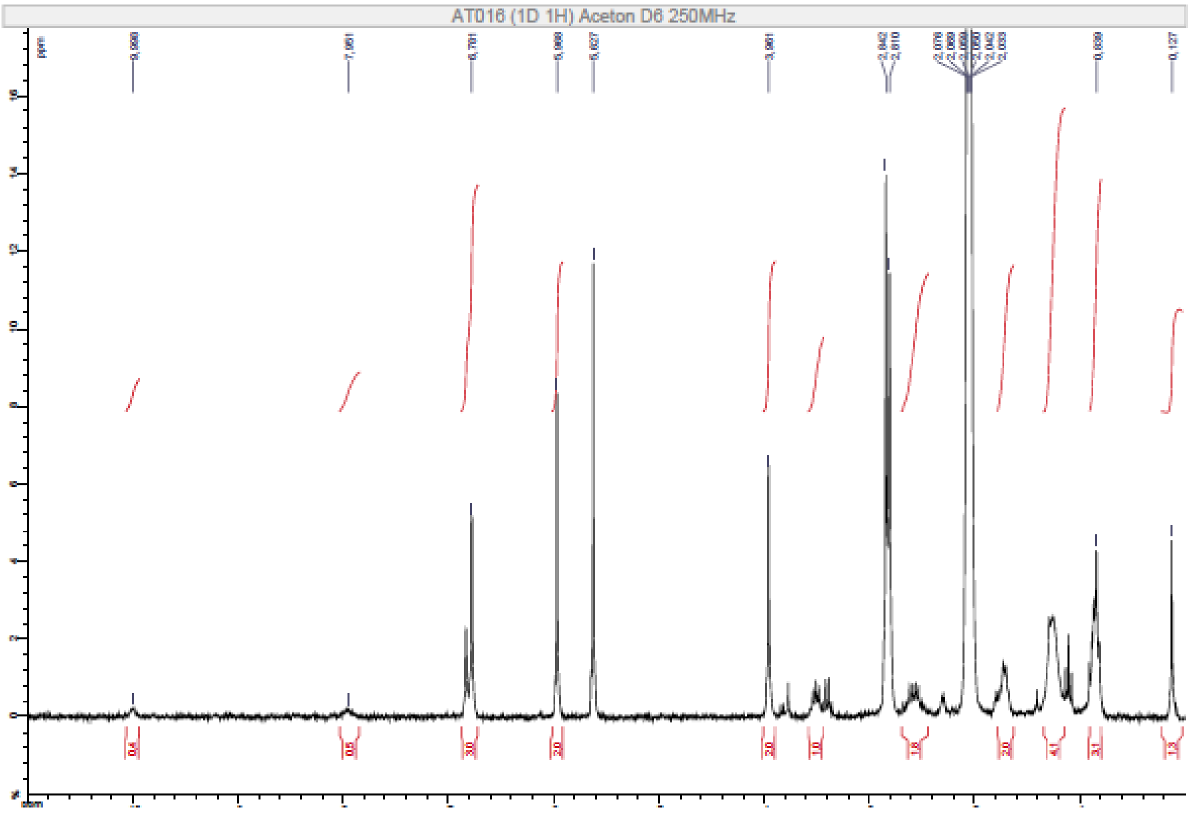

AT017

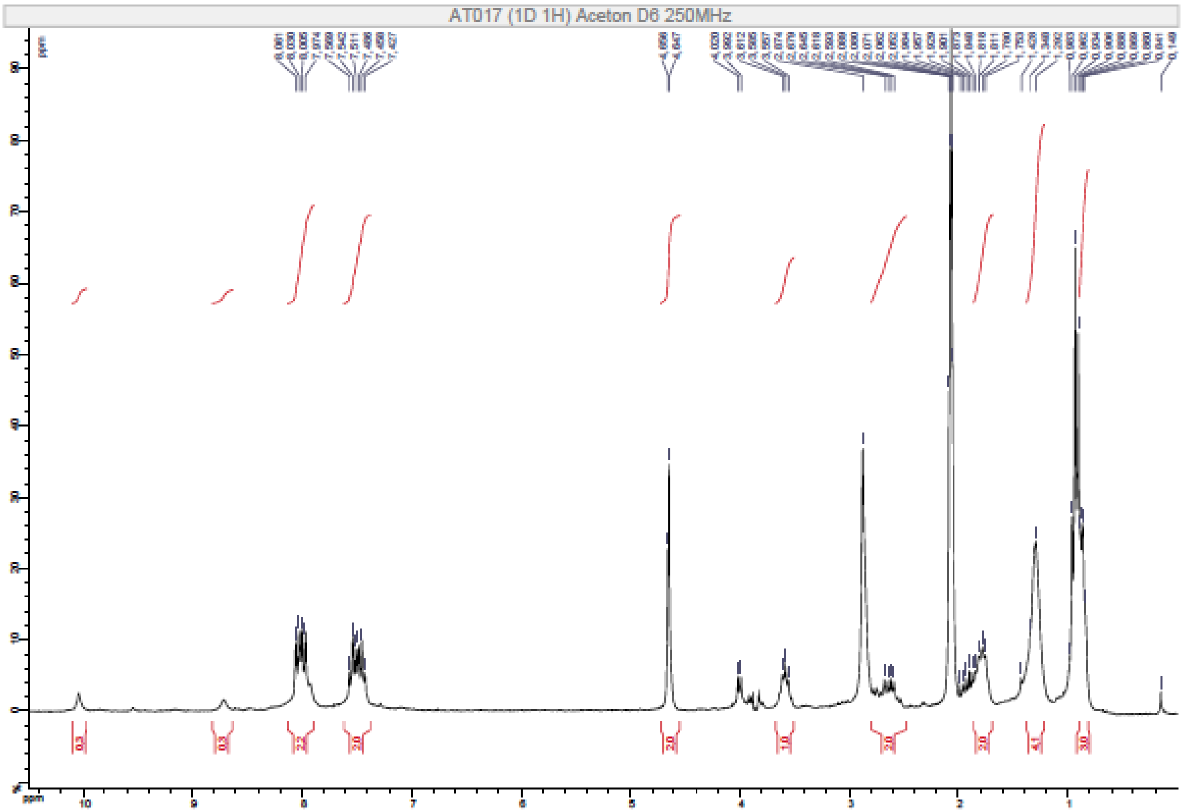

AT018

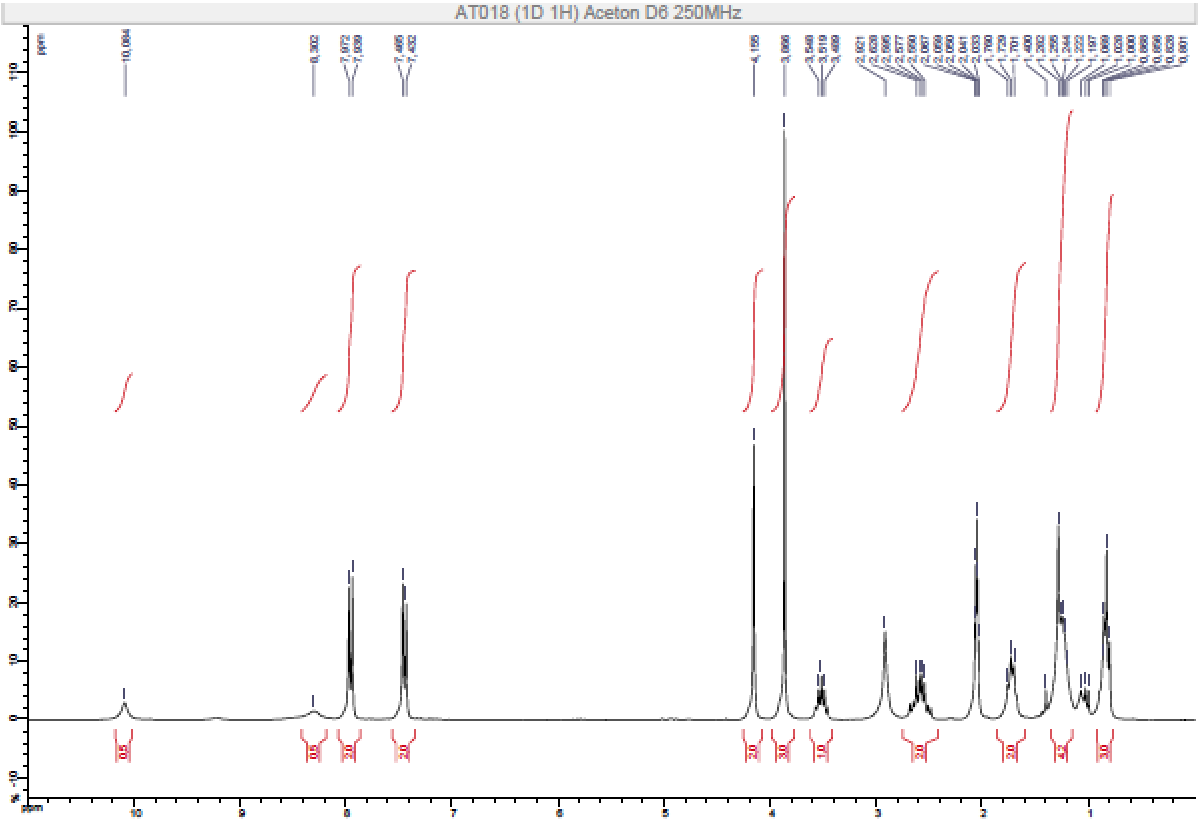

AT019

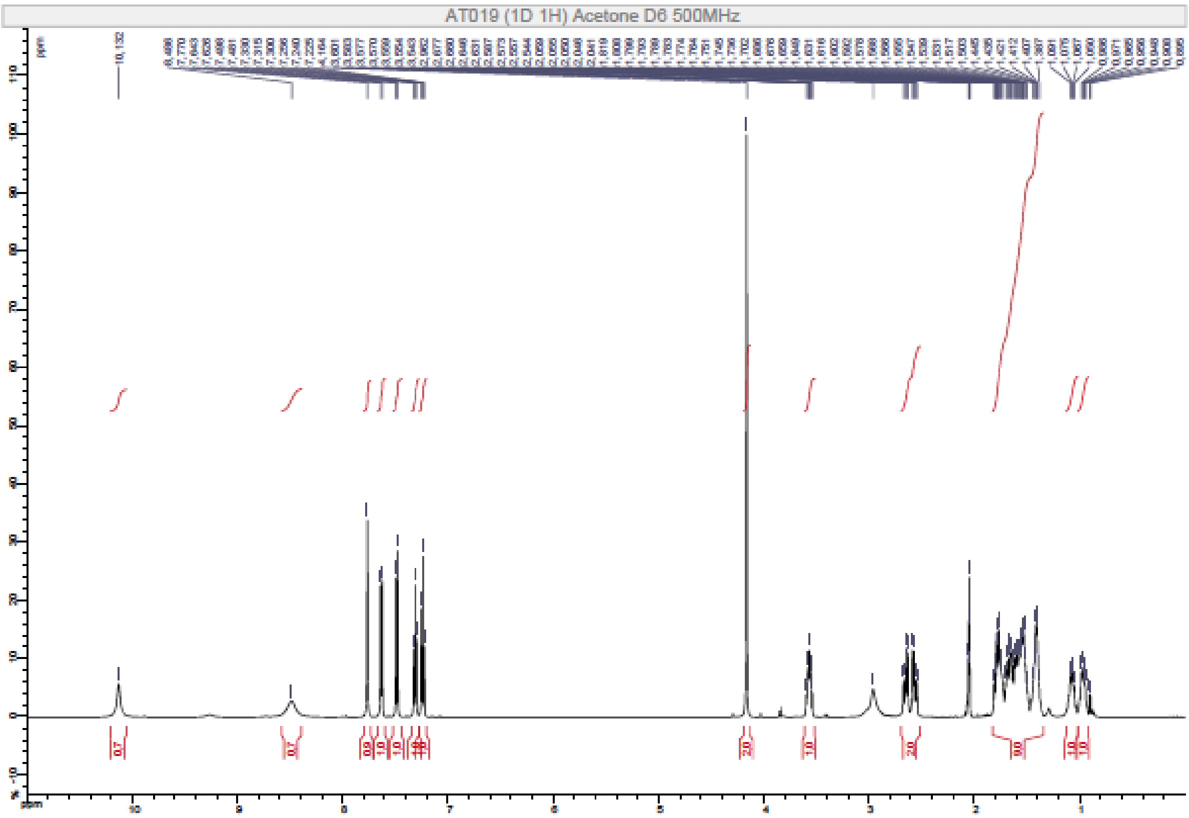

AT020

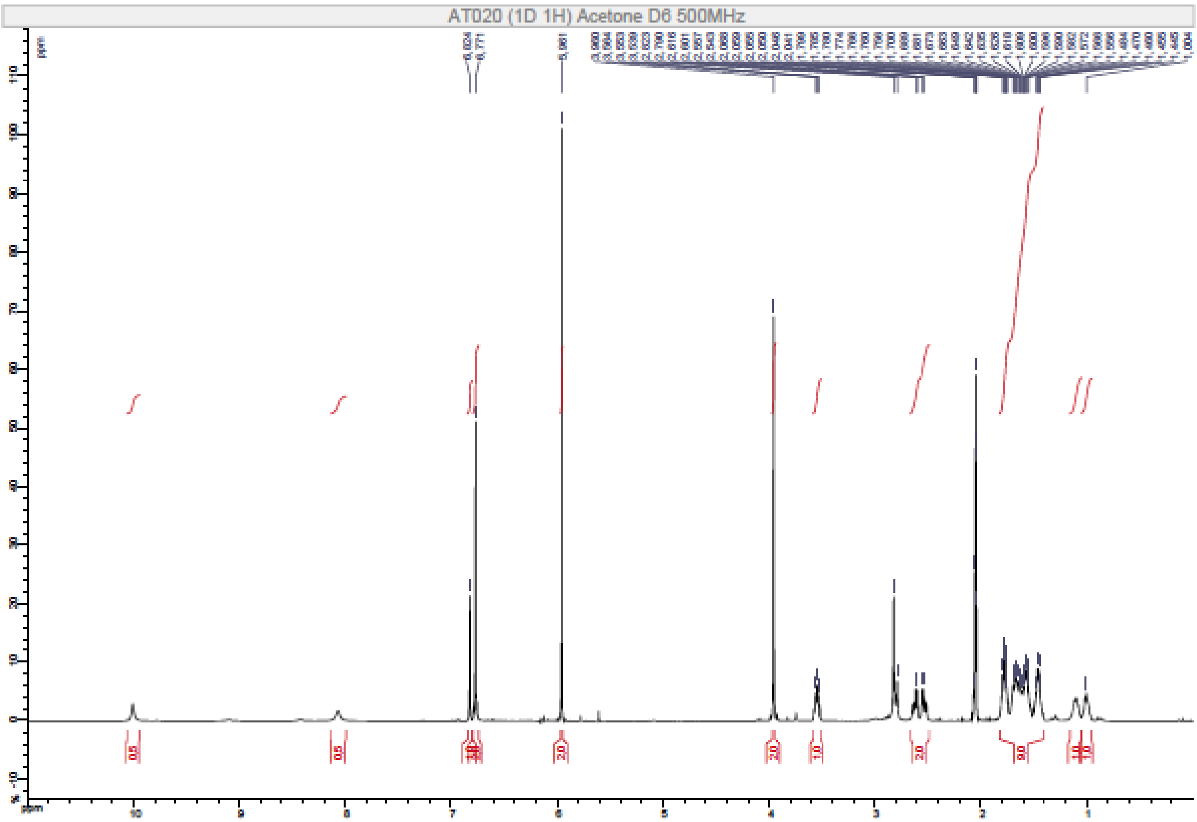

AT021

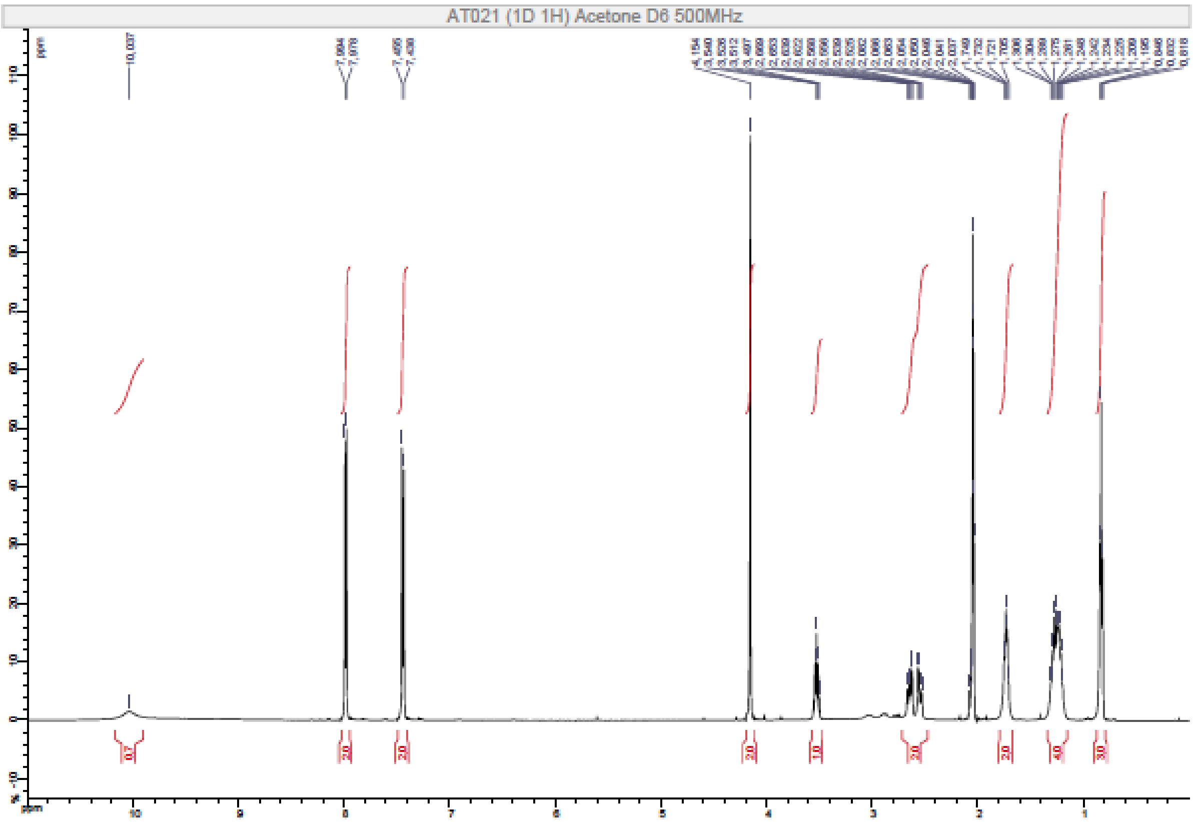

Supplement: Supplementary Information [file srep35429-s1.pdf]
